# Zymograph profiling reveals a divergent evolution of sirtuin that may originate from class III enzymes

**DOI:** 10.1016/j.jbc.2023.105339

**Published:** 2023-10-12

**Authors:** Yujiao Yang, Siwei Zou, Kezhu Cai, Ningning Li, Zhongyue Li, Wei Tan, Wei Lin, Guo-Ping Zhao, Wei Zhao

**Affiliations:** 1 CAS Key Laboratory of Synthetic Biology, CAS Center for Excellence in Molecular Plant Sciences, Shanghai Institute of Plant Physiology and Ecology, Chinese Academy of Sciences, Shanghai, China; 2 CAS Key Laboratory of Quantitative Engineering Biology, Shenzhen Institute of Synthetic Biology, Shenzhen Institutes of Advanced Technology, Chinese Academy of Sciences, Shenzhen, China; 3 University of Chinese Academy of Sciences, Beijing, China; 4 Department of Materials Science and Engineering, School of Engineering, Southern University of Science and Technology, Shenzhen, China; 5 Department of Pathogen Biology, School of Medicine & Holistic Integrative Medicine, Nanjing University of Chinese Medicine, Nanjing, China

**Keywords:** sirtuins, deacylase, activity profiling, zymograph, molecular evolution

## Abstract

Sirtuins are a group of NAD^+^-dependent deacylases that conserved in three domains of life and comprehensively involved in the regulation of gene transcription, chromosome segregation, RNA splicing, apoptosis, and aging. Previous studies in mammalian cells have revealed that sirtuins not only exist as multiple copies, but also show distinct deacylase activities in addition to deacetylation. However, the understanding of sirtuin zymographs in other organisms with respect to molecular evolution remains at an early stage. Here, we systematically analyze the sirtuin activities in representative species from archaea, bacteria, and eukaryotes, using both the HPLC assay and a 7-amino-4-methylcoumarin-based fluorogenic method. Global profiling suggests that the deacylase activities of sirtuins could be divided into three categories and reveals undifferentiated zymographs of class III sirtuins, especially for those from bacteria and archaea. Nevertheless, initial differentiation of enzymatic activity was also observed for the class III sirtuins at both paralog and ortholog levels. Further phylogenetic analyses support a divergent evolution of sirtuin that may originate from class III sirtuins. Together, this work demonstrates a comprehensive panorama of sirtuin zymographs and provides new insights into the cellular specific regulation and molecular evolution of sirtuins.

Sirtuins are a family of NAD^+^-dependent deacylases with widespread distribution throughout the three domains of life, catalyzing the removal of acyl groups from the *ε*-amino moiety of lysine residues in diverse proteins (1, 2, 3) (Fig. 1*A*). Sirtuin activity was first characterized as a deacetylase, and new enzymatic activities, such as desuccinylase (4), depalmitoylase (5), decrotonylase (6), and delactylase (7), have been continuously revealed in the past few years. Sirtuins with new activities have been demonstrated in the regulation of glucose metabolism, germ cell differentiation, tumor necrosis factor-α secretion, and aging (4, 5, 6, 7). Generally, mammalian cells contain seven copies of sirtuin paralogs. These sirtuins localize at distinct cellular compartments and show specific enzyme activities, corresponding to the regulation of different signaling pathways (8, 9). In contrast, much less is known about the function of sirtuins in prokaryotes. Whether or not prokaryotic sirtuins possess the newly discovered activities remains unclear, and if so, it is unknown whether their activities have been differentiated.

**Figure 1:**
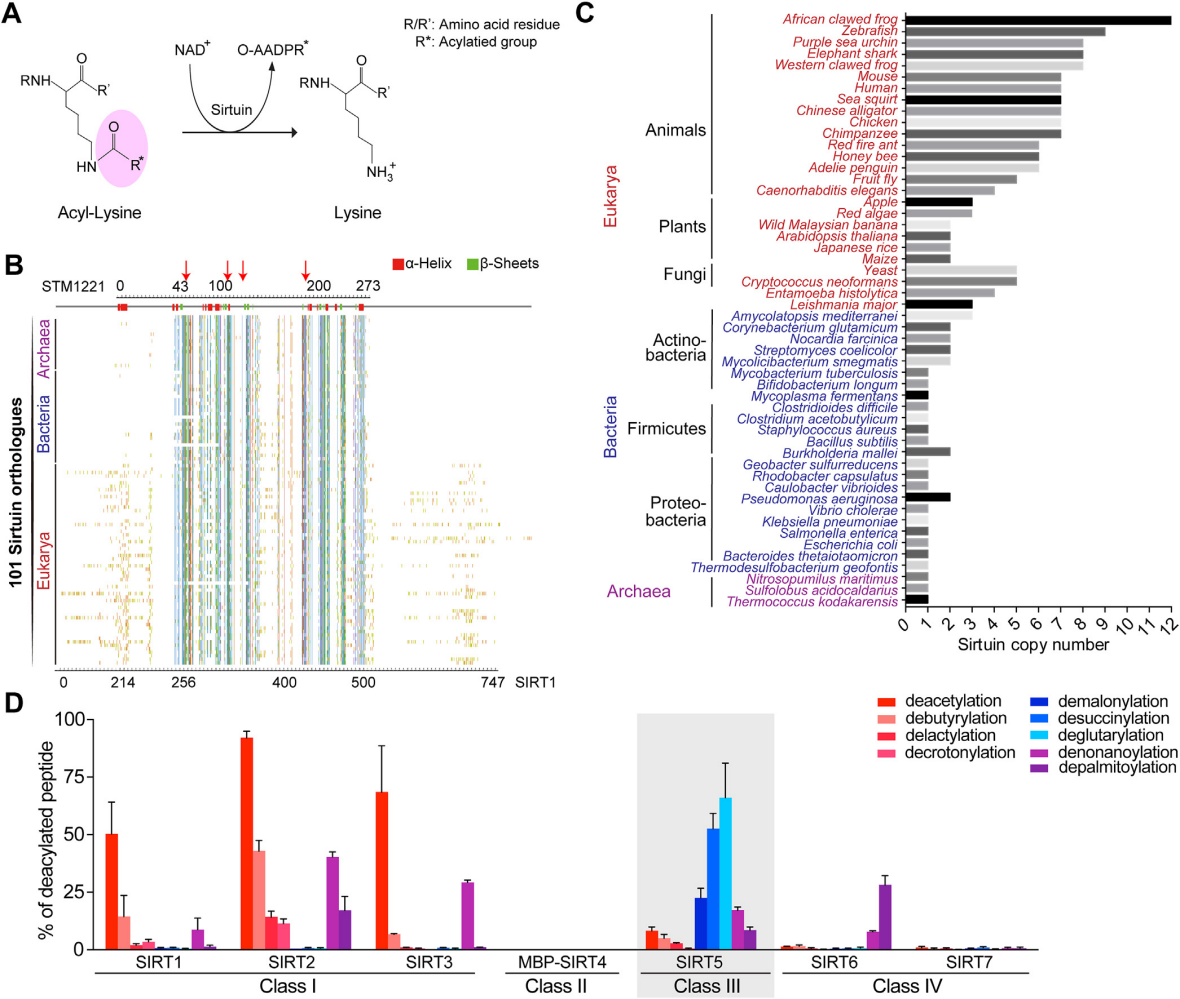
**Sirtuins are conserved in sequences but vary in activities and copy numbers.***A*, a schematic of the deacylation reaction catalyzed by sirtuin. *B*, sirtuins are conserved at the level of amino acid sequences. A total of 101 sirtuin proteins (see Table S6) were selected from the representative species in three domains of life. Multiple sequence alignment of these sirtuins highlights the catalytic core region, displayed by Jalview (39) using the Clustal X color scheme (40). The approximate positions of α-helices and β-sheets were predicted with Jpred 3 (41). *C*, sirtuins vary in copy numbers in three domains of life. The copy numbers were calculated in model organisms and the results indicate they were roughly increased from archaea and bacteria to eukaryotes. *D*, the human sirtuins (SIRT1-7) possess varied activities. The enzyme activities were determined by HPLC using nine different modified H3K27 peptides (ATKAARK∗SAPATG). The reaction mixture contained 5 μM sirtuin, 1 mM peptide, and 1 mM NAD^+^, and was incubated at 37 °C for 2 h. The deacylation activity of sirtuin was determined by calculating the ratio of deacylated peptides. Data are represented as mean ± SEM (n = 3). See Experimental procedures for details.

Archaea and bacteria usually possess only one or two copies of sirtuins (10, 11). The most studied prokaryotic sirtuin is CobB from *Escherichia coli* and it has been reported to catalyze multiple deacylation reactions, including deacetylation, desuccinylation, depropionylation, and delactylation (12, 13, 14). Meanwhile, a study in *Archaeoglobus fulgidus* found two copies of sirtuins, Sir2Af1 and Sir2Af2, preferentially removed succinyl and myristoyl groups, respectively (11). Our previous studies have also demonstrated that two sirtuin proteins possess different enzyme activities and are involved in regulating distinct metabolic pathways in *Streptomyces coelicolor* (15, 16). These observations indicate that the sirtuin proteins may have started to differentiate in prokaryotes, but the range and extent of differentiation remain unknown.

A systematic understanding of sirtuin activities is necessary because these enzymes are closely related to the specific regulation of cell physiology (17, 18). Without fully understanding zymograph, sirtuin with ascribed activity may play physiological roles through other deacylation events. For example, the human SIRT5 was originally identified as a deacetylase, but was later shown to be a main desuccinylase, regulating the activity of carbamyl phosphate synthase *in vivo* (4). For these undifferentiated or incipient differentiated sirtuins, it is particularly necessary to understand the cellular substrates and functions that are regulated by specific activities.

A systematic understanding of sirtuin activities would also shed significant light on the molecular evolution of sirtuins (2, 19, 20). Based on the conservation of a ∼250 amino acid core region, sirtuins have been assigned into five classes, including classes I-IV and an intermediate class U (21). Mammalian SIRT1-3 belong to class I while SIRT4 belongs to class II. SIRT5 is a class III sirtuin and SIRT6-7 are class IV enzymes. Frye (21) suggests that the sirtuin proteins may have undergone a molecular evolution that resulted from an α-proteobacterium engulfment by an archaeon, creating the first eukaryotic cell that received its class III sirtuin (SIRT5 ortholog) from the archaeon parent and its class II sirtuin (SIRT4 ortholog) and class U sirtuin from the α-proteobacterium parent. Recently, new sirtuin family members have been reported in vertebrates by evolutionary analyses, belonging to class I and class IV enzymes, respectively (19, 20). Nevertheless, how the sirtuin evolved into multiple copies and the underlying mechanisms of enzymatic differentiation remain elusive.

In the current study, we developed a high-throughput method to delineate sirtuin zymographs by adopting a 7-amino-4-methylcoumarin (AMC)-based fluorogenic assay (22). The sirtuins were collected from representative species of archaea, bacteria, and eukaryotes, and their deacylation activities were determined in a heterogeneous expression system. The enzyme activities were further verified by a conventional HPLC method. Our results not only provide a comprehensive panorama of sirtuin zymographs, but also reveal an undifferentiated zymograph of class III sirtuins. Phylogenetic analysis and enzyme kinetic determination suggest a gradual differentiation of sirtuin in deacylase activities from prokaryotes to eukaryotes, which is possibly derived from class III enzymes.

## Results

### The copy number and enzyme activity of sirtuins vary in different organisms

As a group of deacylases, sirtuins are highly conserved at the level of amino acid sequence (1). However, the variations of sirtuins in different organisms are barely known. To examine this, a total of 101 sirtuins were selected from 44 representative organisms to perform a multiple sequence alignment. The results revealed a conserved core region (∼230 amino acids) flanked by two variable regions at the N terminus and the C terminus, especially for eukaryotic sirtuins (Fig. 1*B*). In addition, the copy number of sirtuins in representative species varied from 1 to 3 in prokaryotes to more than ten in eukaryotes (Fig. 1*C*). Interestingly, although plants encode the most genes in nature, the sirtuin copy number was not the highest (only 2–3 sirtuins per genome), implying sirtuin variations are closely related to physiological and environmental regulation.

Sirtuins have been shown to be able to catalyze an increasing number of deacylations (23, 24). A previous study used a TLC assay demonstrated that human SIRT1-6 had diverse but overlapping specificity for different acylated peptides (25). In the current study, we applied an HPLC method to quantify nine distinct deacylation activities for all seven human sirtuins. These deacylation activities include the newly discovered delactylation, denonanoylation, and depalmitoylation. Similar to previous observations (25), the human sirtuins displayed varied and differentiated zymographs (Fig. 1*D*). Specifically, SIRT1-3 showed the main deacetylase activity, SIRT5 showed the main desuccinylase activity, and SIRT6 exhibited more eﬃcient defatty-acylase activity. No significant deacylase activity was detected for SIRT4 or SIRT7, though they were successfully purified from *E. coli* using soluble tags. Significantly, nearly all tested activities were observed in SIRT5, indicating this enzyme has a relatively undifferentiated zymograph, compared to other human sirtuins (Fig. 1*D*).

### The sole sirtuin in *Salmonella* has an undifferentiated zymograph

**Figure 2:**
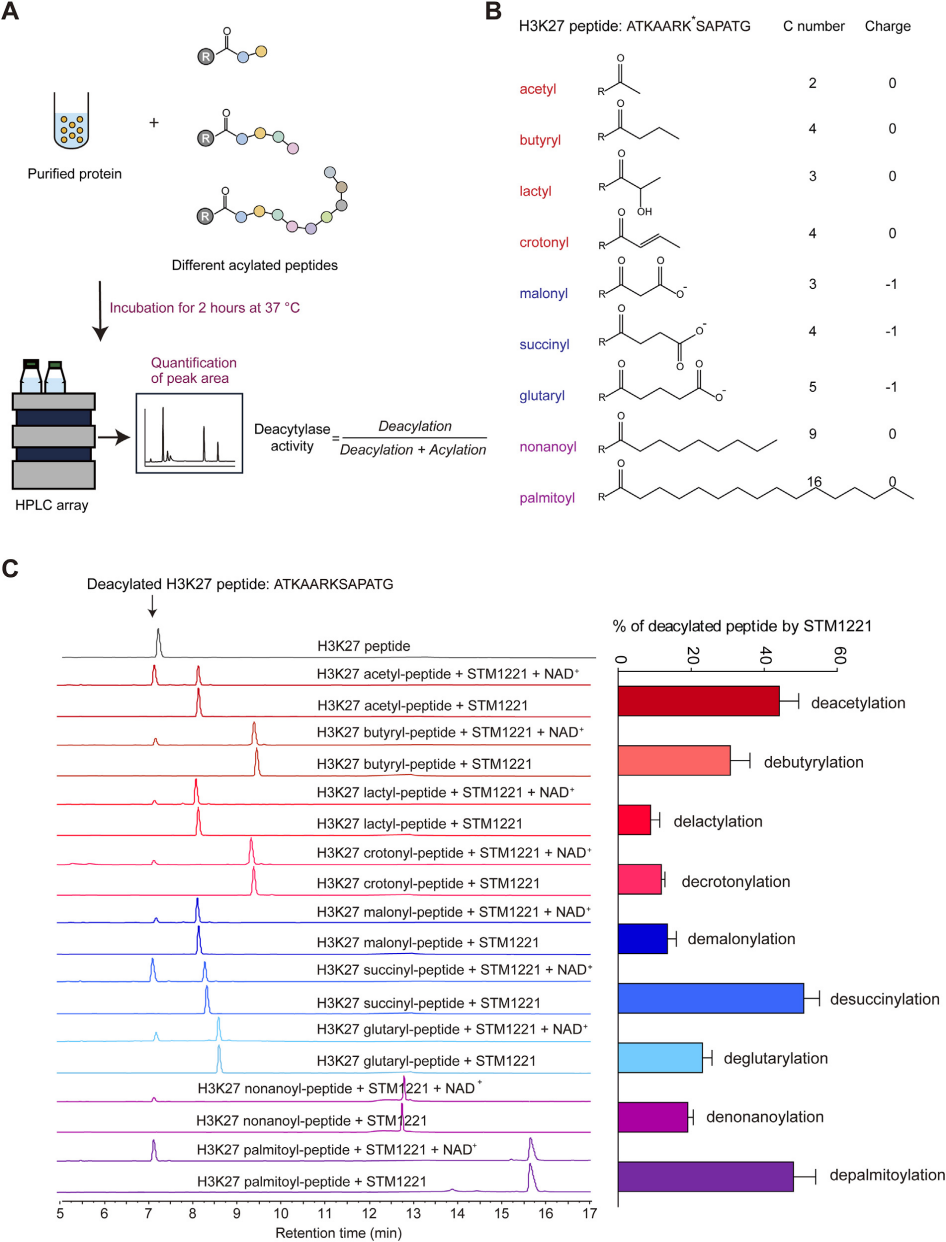
**The sirtuin from *Salmonella* displays an undifferentiated zymograph.***A*, schematic of activity profiling for STM1221 using the HPLC method. STM1221 was purified and incubated with different acylated peptides and the peak areas of reactants and products were measured after a 2-h incubation. *B*, the synthesis of nine acylated H3K27 peptides. These peptides can be divided into three categories with different carbon lengths and charge states, as indicated by distinct colors. *C*, STM1221 shows an undifferentiated deacylation zymograph. The reaction mixture containing 5 μM STM1221, 1 mM peptide, and 1 mM NAD^+^ was incubated at 37 °C for 2 h, and the enzymatic activity was evaluated by HPLC as described in panel (*A*). The *top gray line* represents the standard unmodified H3K27 peptide. The reactions without NAD^+^ were used as the negative controls. Quantitative analyses of STM1221 activities are shown on the *right panel*. Data are represented as mean ± SEM (n = 3).

Human SIRT5 belongs to class III sirtuins, and bacterial *Salmonella enterica* also has a class III sirtuin named STM1221 (26). Previous reports have suggested that STM1221 possesses multiple deacylation activities, including deacetylation (26, 27) and de-2-hydroxyisobutyrylation (28). To investigate whether the bacterial sirtuin has any other deacylase activities, we purified STM1221 and examined its zymograph by HPLC (Fig. 2*A*).

A series of histone H3 peptides with various acylations, ranging in carbon lengths, charge states, and saturations were synthesized and tested as substrates of STM1221 (Fig. 2*B*). The corresponding enzyme activities were quantified by calculating the conversion ratio of acylated peptides (Fig. 2*A*). STM1221 was able to remove all the tested acyl groups from the *ε*-amino moiety of lysine residues, albeit with different deacylase activities. Besides the known deacetylase and desuccinylase activities, STM1221 exhibited a relatively high long-chain deacylase activity, with conversion ratios of 19.06% and 48.02% for denonanoylation and depalmitoylation in 2 h, respectively (Fig. 2*C*). In addition, STM1221 also showed a newly discovered activity of delactylation (conversion ratio: 8.89%), which is similar to the recent report for its ortholog, CobB, from *E. coli* (14) (Fig. 2*C*). Collectively, these results suggest an undifferentiated zymograph for the bacterial sirtuin STM1221.

Based on the analyses of STM1221 and human sirtuins (Figs. 1*D* and 2), we divided the sirtuin enzyme activities into three categories for simplicity: the short-chain deacylases, such as deacetylation and debutyrylation; the long-chain deacylases, such as demyristoylation and depalmitoylation; and the negatively charged-chain deacylases, such as demalonylation and desuccinylation.

### Activity profiling reveals an undifferentiated zymograph of class III sirtuins

**Figure 3:**
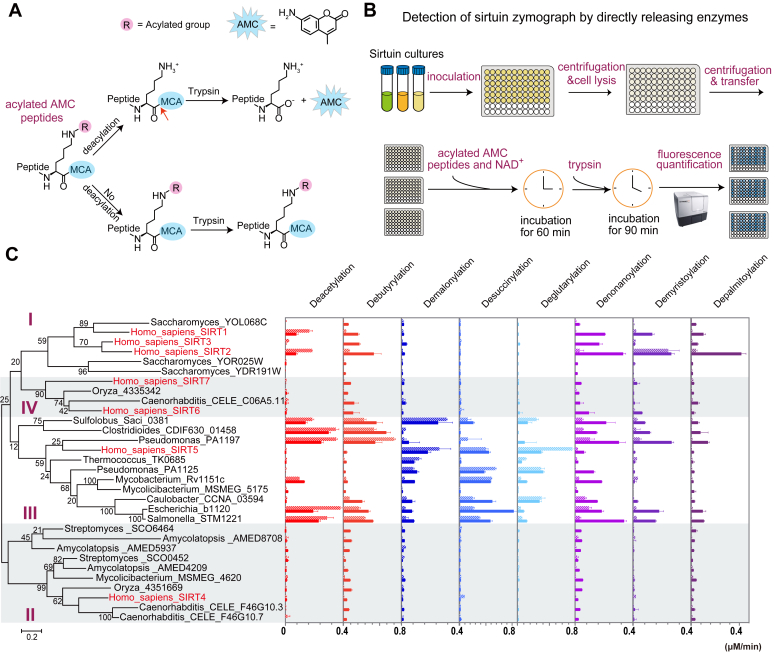
**Global profiling reveals undifferentiated zymographs ofclass III sirtuins.***A*, schematic overview of the AMC-based fluorogenic assay for sirtuin activity evaluation. Briefly, if a specific deacylase activity of sirtuin was detected, the corresponding acyl group (R) could be removed from the peptide substrate (22). Without the protection of the acyl group, the peptide could be recognized by trypsin and a fluorescent AMC molecule would be released by the trypsin digestion. Otherwise, the acylated peptide would not be recognized by trypsin and no AMC signal would be observed. Fluorescence measurements were executed at λ_ex_ = 360 nm and λ_em_ = 460 nm. The *red arrow* indicates the cleavage site of trypsin. *B*, schematic of zymograph detection by directly releasing sirtuin enzymes combined with fluorogenic quantification. *Escherichia coli* Δ*cobB* cells expressing sirtuin enzymes were induced in a 96-well plate, and then lysed *in situ* by lysis buffer containing 1% (m/v) NP-40, 25 mM Tris–HCl (pH 8.0), and 150 mM NaCl at 37 °C for 15 min with gentle stirring. After centrifugation, the supernatants were transferred to a new plate and reacted with 1 mM NAD^+^ and 200 μM acylated AMC peptides. After incubation at 37 °C for 60 min, the supernatants were treated with 2.5 mg/ml trypsin at 25 °C for another 90 min followed by fluorescence quantification. *C*, global activity profiling reveals undifferentiated zymographs of class III sirtuins. Phylogenetic analysis of sirtuins and the quantification of corresponding deacylase activities are shown. The phylogenetic tree was built with the maximum likelihood method using the amino acid sequences of 31 sirtuins, which were selected from the model organisms of three domains of life. The deacylation reactions were performed using 2 distinct peptides (LGK∗, striped; ARK∗, monochromatic) with eight different acylated modifications. The fluorescent results were normalized by the expression level of each sirtuin and are shown as the production rates of AMC molecules by 10 μM of sirtuins. Data are represented as mean ± SEM (n = 3). AMC, 7-amino-4-methylcoumarin.

Phylogenetic analysis of the 101 sirtuins revealed they were grouped into four major classes, and the previous reported class U and class I (21) were integrated into the new class I (Fig. S1). In this phylogenetic tree, nearly all archaeal sirtuins, most bacterial sirtuins, and the mammalian SIRT5 were grouped as class III (Fig. S1). We asked whether all sirtuins from class III had undifferentiated zymographs as that of STM1221. To answer this question, a high-throughput fluorogenic detection method (22) was used to analyze the deacylase activities of sirtuins (Fig. 3*A*). In principle, if the tested sirtuin had a specific deacylase activity, the corresponding peptide could be deacylated and then recognized by trypsin, which would result in the release of a highly fluorescent AMC molecule after trypsin digestion. Otherwise, the acylated peptide would not be the substrate of trypsin and no AMC signal could be detected (Fig. 3*A*). The fluorescence signals increased in direct proportion to the amount of deacylated peptide substrates (Fig. S2*A*).

We adopted this method by combining it with *in-situ* lysis and then detected the sirtuin activities in a 96-well plate (Fig. 3*B*). Eight different acylations on two distinct peptide sequences with a 4-methylcoumarin-7-amide moiety at the C terminal of each peptide were synthesized. A total of 31 representative sirtuins including 11 class III enzymes were successfully expressed in *E. coli* Δ*cobB* cells (27) (Fig. 3*C* and Table S6). After adding a specific 4-methylcoumarin-7-amide -modified peptide and an NAD^+^ cofactor, the corresponding enzyme activity of sirtuin was quantified in *E. coli* culture by fluorospectrophotometry. The results were further normalized by protein expression levels using the ELISA (Fig. S2*B*). In total, 1488 fluorescence read results with three independent biological repetitions were obtained in about 5 h. The resulting 31 zymographs were classified into four groups, which corresponded well with the four sirtuin classes (Fig. 3*C*). In brief, the group of class I sirtuins showed zymographs of both short- and long-chain deacylase activities, while the group of class II sirtuins displayed a tendency to remove long-chain acyl moieties, and the group of class IV sirtuins had no significant activity for any of the acylated peptides. Interestingly, the group of class III sirtuins showed deacylase activities for all eight acyl moieties, indicating undifferentiated zymographs for these sirtuins (Fig. 3*C*). Although catalytic preferences were observed when using different peptide substrates, the patterns of 31 sirtuin zymographs were generally the same for the two peptides LGK∗ and ARK∗ (∗ refers to any kind of acylation) (Fig. 3*C*).

To validate the sirtuin zymographs, an HPLC assay was used to quantify the deacylase activities of the sirtuins for the H3K27 peptide (ATKAARK^27^SAPATG). This peptide was modified at K27 by nine different acylations, including short chains, long chains, and negatively charged chains (Fig. S3). In total, 17 enzymes were successfully purified among the 31 sirtuins mentioned above, and then incubated with different acylated H3K27 peptides. The obtained zymograph patterns were consistent with those measured by the AMC-based fluorogenic method (Fig. 3*C*), and the undifferentiated zymographs of class III sirtuins were observed as well (Fig. S3). Nevertheless, the sirtuins appeared to have higher deacylation activities when assayed by the HPLC-based method, possibly because this method used relatively longer peptide substrates that would bind to sirtuins more efficiently (25). Collectively, these data confirmed that sirtuin activities could be divided into three categories and revealed an undifferentiated zymograph of class III sirtuins. Moreover, the results suggest a tight relationship between deacylation activity and the classification of sirtuins.

### Identification of initial enzymatic differentiation in class III sirtuins

We next focused on the class III zymographs and found that initial enzymatic differentiation already occurred in some sirtuins. A subgroup of sirtuins, typified by *Sulfolobus* Saci_0381, had relatively higher short- and long-chain deacylase activities. Another subgroup of sirtuins, typified by *Pseudomonas* PA1125, displayed a higher tendency to remove negatively charged chain acyl moieties, while the other subgroup of sirtuins, typified by *Salmonella* STM1221, displayed balanced (all high) deacylase activities (Figs. 3*C* and S3).

**Figure 4:**
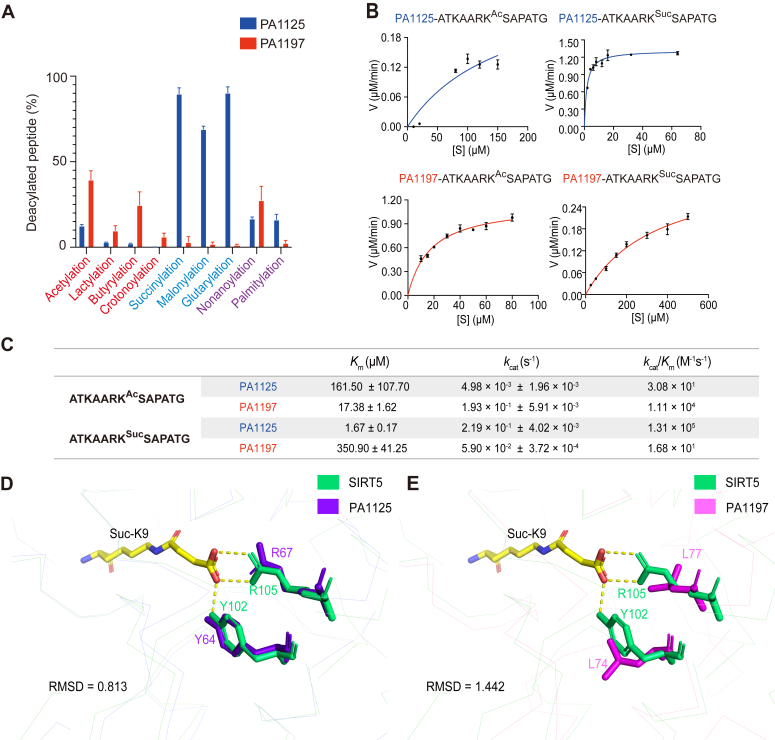
**Identification of initial enzymatic differentiation in class III sirtuin orthologs.***A*, PA1125 shows a higher tendency to remove negatively charged chain acylations while PA1197 displays a higher tendency to remove short-chain acylations. The enzymatic activities were determined by HPLC assay. The reaction mixtures containing 5 μM sirtuin and 1 mM peptide were incubated at 37 °C for 2 h, and the deacylation activity was determined by calculating the ratio of deacylated peptides. Data are represented as mean ± SEM (n = 3). *B*, the activity discrepancy between PA1125 and PA1127 was confirmed by enzyme kinetic analyses. Michaelis–Menten plots for PA1125 and PA1197 against acetylated or succinylated H3K27 peptide substrates are shown. Data are represented as mean ± SEM (n = 3). *C*, steady-state parameters of PA1125 and PA1197 against modified H3K27 substrates. The quantitative results are related to the curves presented in Figure 4*B*. *D* and *E*, structural analyses suggest PA1125 has a better overlap with the human SIRT5 than that of PA1197. The structures of PA1125 (*D*) and PA1197 (*E*) were modeled by Alphafold2 and overlapped with SIRT5 (PDB entry: 3RIG). Root-mean-square-deviation (RMSD) was calculated to measure their similarities. Two sites of PA1125 (Y64 and R67) are predicted to interact with the succinylated H3K9 peptide, corresponding to Y102 and R105 in human SIRT5, respectively. PDB, Protein Data Bank.

**Figure 5:**
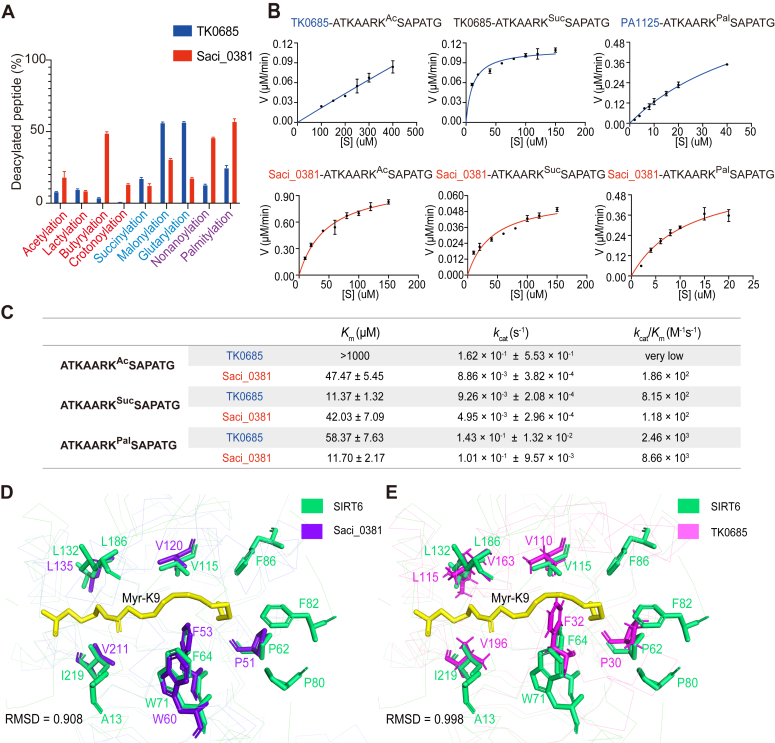
**Identification of initial enzymatic differentiation in class III sirtuin paralogs.***A*, TK0685 shows a higher tendency to remove negatively charged chain acyl moieties, whereas Saci_0381 displays a better ability to remove short- and long-chain acyl moieties. The enzymatic differentiation between TK0685 and Saci_0381 was determined by HPLC. The reaction mixtures containing 5 μM sirtuin and 1 mM peptide were incubated at 37 °C for 2 h, and the deacylation activity was determined by calculating the ratio of the deacylated peptide. Data are represented as mean ± SEM (n = 3). *B*, the activity discrepancy between TK0685 and Saci_0381 was confirmed by enzyme kinetic analyses. Michaelis–Menten plots for TK0685 and Saci_0381 against acetylated, succinylated, and palmitoylated H3K27 peptide substrates are shown. Data are represented as mean ± SEM (n = 3). *C*, steady-state parameters of TK0685 and Saci_0381 against modified H3K27 substrates. The quantitative results are related to the curves presented in Figure 5*B*. *D* and *E*, structural analyses suggest Saci_0381 has a better overlap with human SIRT6 than that of TK0685. The structures of Saci_0381 (*D*) and TK0685 (*E*) were modeled by Alphafold2 and overlapped with SIRT6 (PDB entry: 3ZG6). RMSD was calculated to measure their similarities. Similar to that of SIRT6, residues in Saci_0381 form a hydrophobic pocket, which could accommodate the long myristoylated peptide. However, the corresponding residue of F32 in TK0685 protrudes in the pocket and may hinder the loading of peptides with long-chain acylations. PDB, Protein Data Bank.

Two sirtuins named PA1125 and PA1197 are encoded in *Pseudomonas aeruginosa*, and both of them belong to class III sirtuins. Specifically, PA1125 showed a higher tendency to remove negatively charged chain acylations while PA1197 displayed a higher tendency to remove short-chain acylations, a result which suggests enzymatic activity differentiated at the paralog protein level (Fig. 4*A*). Meanwhile, two archaeal sirtuin orthologs (TK0685 and Saci_0381) were encoded in *Thermococcus* and *Sulfolobus*, which also belong to class III sirtuins. TK0685 showed more efficient removal of negatively charged chain acyl moieties, whereas Saci_0381 was better at removing short- and long-chain acyl moieties (Fig. 5*A*). Together, these results demonstrate that initial differentiation occurred at both paralog and ortholog levels for class III sirtuins. The discrepancies in their activities were further verified by enzyme kinetic analyses. Generally, higher substrate binding affinities (*K*_m_) and faster turnovers (*k*_cat_) were shown for the preferred substrates (Figs. 4, *B* and *C* and 5, *B* and *C*). However, a larger discrepancy was observed between PA1125 and PA1197 (1000–10,000-fold difference in *k*_cat_/*K*_m_) than between TK0685 and Saci_0381 (4-8-fold difference in *k*_cat_/*K*_m_), suggesting a higher differentiation between the sirtuin paralogs in *P. aeruginosa.*

Structural analysis using AlphaFold2 revealed that PA1125, instead of PA1197, had a better overlap with the structure of human SIRT5, especially around the typical active centers. The catalytic Y64 and R67 residues of PA1125 matched perfectly with the Y102 and R105 residues of SIRT5, the latter of which were demonstrated as the key sites for desuccinylation (4) (Fig. 4*D*). On the other hand, the corresponding residues in PA1197 were L74 and L77. Obviously, these two sites would not preferentially interact with the negatively charged succinyl moiety (Fig. 4*E*). The conclusion was further supported by site-directed mutagenesis of PA1125 into PA1125 (Y64L and R67L), which abolished the desuccinylase activity of PA1125 (Fig. S4, *A* and *B*). On the other hand, the desuccinylase activity of PA1197 was not increased when mutated to PA1197 (L74Y, L77R) (Fig. S4*A*), suggesting more sites were involved in the regulation of desuccinylase activity of sirtuins.

In addition, the structural analysis revealed that Saci_0381 developed a large active center similar to that of defatty-acylase SIRT6 (5, 29). The residues P51, F53, W60, V211, L135, and V120 formed a large hydrophobic pocket in Saci_0381, which may have allowed it to accommodate a relatively long-chain acyl moiety (Fig. 5*D*). In contrast, a smaller catalytic pocket was observed in TK0685 by structure simulation. In particular, one of the residues around the active center, F32, tended to shrink the hydrophobic pocket and may have hindered the loading of peptides with long-chain acylations (Fig. 5*E*). Consistent with this hypothesis, mutation of F32 to a smaller alanine (F32A) in TK0685 increased its denonanoylase activity (Fig. S5). Together, these observations demonstrate that the underlying mechanisms of enzymatic differentiation are driven by the primary sequences around the catalytic active center of sirtuins, though other sites may also be involved in the regulation.

### Systematic analyses indicate a divergent evolution that may originate from class III sirtuins

A previous report (21) suggested that the sirtuin proteins likely evolved from a class II/U sirtuin parent (α-proteobacterium origin) and a class III sirtuin parent (archaea origin). However, all current sirtuins in α-proteobacteria are classified as class III enzymes rather than class II or class U enzymes. Moreover, a significant number of eukaryotes such as yeast and worms do not possess any class III sirtuins. Therefore, the evolution of sirtuins may be more complicated than expected (19, 20).

**Figure 6:**
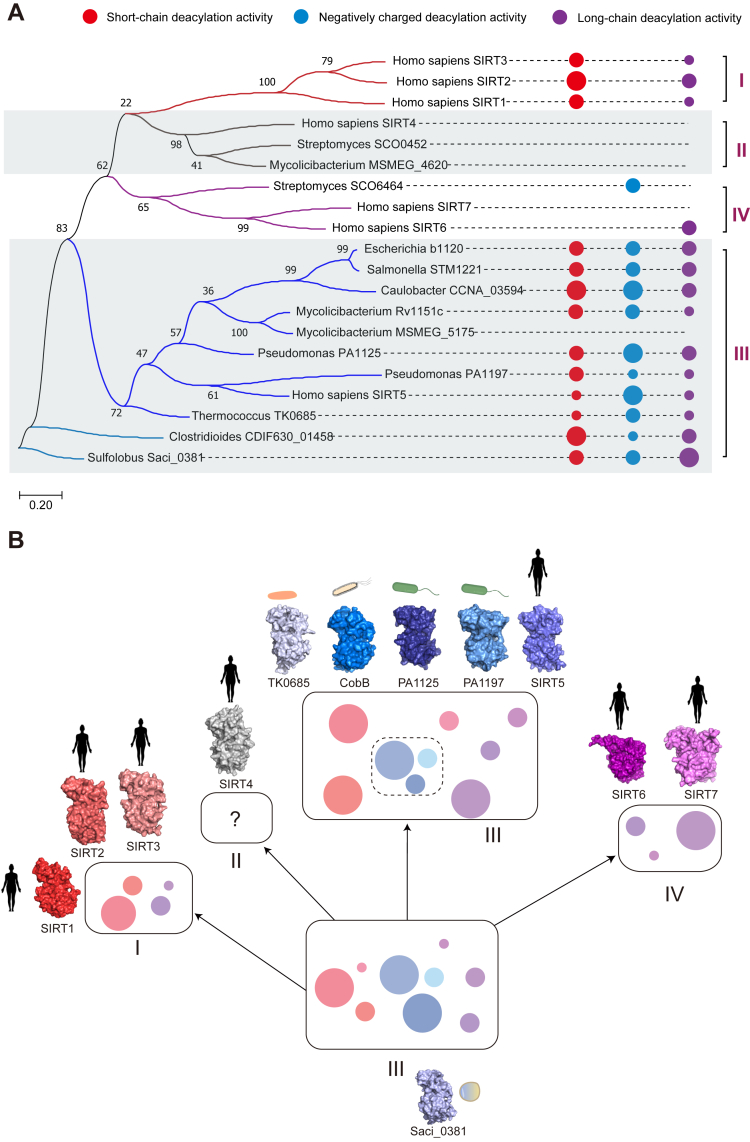
**Systematic analyses support a divergent evolution of sirtuin.***A*, the enzymatic differentiation matches well with the phylogenetic evolution of sirtuin molecules. A phylogenetic tree was generated by MEGA X (35) using the maximum likelihood method with the core regions (∼230 aa) of the 31 amino acid sequences of sirtuins. The *circles* with different colors represent different activity categories of sirtuins, and the circle sizes indicate the deacylation capacities. For simplicity, the *small-sized circles* represent deacylase activity of 2 to 10%, the *medium-sized* circles represent deacylase activity of 10 to 50%, and *large-sized circles* represent the deacylase activity of more than 50%, as determined by the HPLC method in the current study. The classification of sirtuins based on phylogenetics was indicated on the right. *B*, a proposed model for sirtuin differentiation and evolution. The sirtuin molecules evolved from the ancestral class III to the descendant classes I-IV, accompanied by enzymatic differentiation and copy number increase. *Circles* with different colors and sizes represent the differences in enzyme activity categories and capacities, respectively. The *red color* indicates the short-chain deacylase activity, the *violet color* indicates the long-chain deacylase activity, and the *blue color* indicates the negatively charged-chain deacylase activity. The *dashed box* indicates that certain sirtuins initially differentiated among class III enzymes. The displayed structures of sirtuins (SIRT4, TK0685, Saci_0381, PA1125, PA1197, and SIRT7) were generated by Alphafold2 modeling (36). SIRT1: 4KXQ; SIRT2: 5G4C; SIRT3: 4BN4; CobB: 1S5P; SIRT5: 5rjs; SIRT6: 3ZG6. All structures are represented by surface patterns using PyMOL (https://pymol.org).

To delineate the intrinsic relationship between functional differentiation and molecular evolution of sirtuins, we mapped the sirtuin zymographs onto a phylogenetic tree built by primary sequences using the maximum likelihood method. The results show that all α-proteobacterial and most archaeal sirtuins were grouped as class III (Figs. 6*A* and S1). Moreover, an evolutionary path from the ancestral class III sirtuins to the descendant classes I-IV sirtuins could be observed when using archaeal Saci_0381 as the tree root. Along with this, the enzymatic activity of sirtuins differentiated from the full zymography to the loss of negatively charged chain or short-chain deacylase activity, or both. Moreover, the copy number of sirtuin increased either by cell engulfment before speciation or by gene duplication after speciation (Fig. 6, *A* and *B*). The hypothesis was further supported when the initial enzymatic differentiation of class III sirtuins in the current species was observed (Figure 4, Figure 5, Figure 6*B* and S6). Collectively, these data support a divergent evolution of sirtuin that may all originate from class III enzymes.

## Discussions

With the development of proteomic technologies, more acylations on lysine residues have been identified and will be constantly updated (1, 24). The corresponding sirtuin(s) responsible for these novel activities need to be comprehensively understood. Moreover, previously assumed activities of sirtuins may not always be correct, and their corresponding zymographs need to be adjusted. In the current study, the activities of sirtuins from archaea, bacteria, and eukaryotes were comprehensively analyzed by both HPLC- and AMC-based methods using distinct acylated peptides. The sirtuin(s) responsible for specific activities in each species were characterized, and their zymographs were profiled accordingly. Moreover, an undifferentiated zymograph of class III sirtuins was revealed. Based on enzyme kinetic and phylogenetic analyses, a divergent evolution of sirtuin that originates from class III enzymes was further hypothesized.

There has been conjecture for years that sirtuin proteins underwent molecular evolution with activity differentiation and copy number increase (2, 21). However, the data presented here may represent the first direct evidence to support this claim. Our experimental results in combination with phylogenetic analyses support an evolutionary path from the ancestral class III sirtuins to the descendant sirtuin groups and indicate an enzymatic differentiation possibly caused by loss of function. The hypothesis refined the existing cell engulfment model and suggests that class III enzymes could be the evolutionary origin for all types of sirtuins.

A full and detailed understanding of sirtuin zymographs and evolution has critical physiological implications. Firstly, it will facilitate the study of the real functions of sirtuins *in vivo* (1, 30). Previous reports about the regulation of sirtuins have been heavily dependent on the application of pan-antibodies and small inhibitors such as nicotinamide (31). However, the phenotypes obtained in these studies could be the result of a combined effect of multiple sirtuins in cells due to the nonspecificity of these methods. In the case of prokaryotic sirtuins, the situation becomes more complex due to their typically undifferentiated zymographs. The claim of regulation by specific activities might be incorrect due to limited evidence by simple KO or overexpression of the sirtuin genes. On the other hand, an understanding of sirtuin zymographs and evolution will facilitate the research and development of precise medicines (32, 33). Sirtuin regulation has been shown to be closely related to various diseases and the specific activities correspond to distinct disease models (1). The identification of target sites that are responsible for enzymatic differentiation is pivotal for drug discovery and may guide future molecular therapy.

Nevertheless, novel methods are still needed to develop fast, accurate, and comprehensive detection of sirtuin activity. Although the zymograph patterns were roughly the same between the AMC-based fluorogenic method and the HPLC assay (Figs. 3*C* and S3), variations in activities were observed for peptides with either different lengths or distinct sequences. Moreover, zymographs for certain sirtuins were still lacking because the difficulties in expressing them in *E. coli* for the AMC-based fluorogenic assay, though more zymographs were obtained using this method than with the conventional HPLC assay. Hence, future directions will focus on the development of more sensitive methods, such as reporting systems based on NAD^+^ consumption or production, or the application of the AMC-based fluorogenic assay in other expression models such as yeast and mammalian cells, to comprehensively determine the sirtuin zymographs. In regards to the molecular evolution of sirtuin, the 3D structure of sirtuins (18) should also be involved to form a multidimensional evolutionary relationship in concert with the primary sequences. Big data analysis and integration of these possible investigation routes will significantly benefit future studies.

## Experimental procedures

### Bacterial strains, media, and materials

All plasmids, strains, and oligonucleotides used in this study are listed in Tables S1–S3. *E. coli* DH5α and *E. coli* BL21 cells were cultured in LB medium at 37 °C and were used as the host strains for standard plasmid manipulation and protein expression, respectively. The *cobB*-deficient strain, *E. coli* BL21(Δ*cobB*) (27), was used to express sirtuin proteins for the characterization of zymographs. The acylated and unmodified H3K27 (ATKAARKSAPATG) peptides were synthesized by Guoping Pharmaceutical with >98% purity. The acylated Ac-ARK∗-AMC and Ac-LGK∗-AMC peptides were synthesized by Guotai Pharmaceutical with >98% purity (Table S4). The AMC-based fluorogenic assay was performed in a black low-binding 384-well microplate (Greiner Bio-One). NAD^+^ (CAT. # N7004) and nicotinamide (CAT. # N0636) were purchased from Sigma-Aldrich, and standard AMC (CAT. # A600044) was purchased from Sangon Biotech.

### Gene synthesis and plasmids construction

The sirtuins used in this study were collected from the representative species of archaea, bacteria, and eukaryotes. All the sirtuins were selected in each representative species. The sirtuin genes *stm1221*, *sco0452*, *sco6464*, *amed4209*, *amed5937*, *amed8708*, *pa1125*, *pa1197*, *msmeg4620*, *msmeg5175*, *cobB*, and *ccna03594* were cloned from *S. enterica* G2466, *S. coelicolor* M145, *Amycolatopsis mediterranei* U32, *P. aeruginosa* PAO1, *Mycobacterium smegmatis* mc (2)155, *E. coli* MG1655, and *Caulobacter crescentus* NA1000, respectively (Table S5). The remaining sirtuin genes in Table S5 were codon optimized and synthesized by Sangon Biotech. Genes were cloned into the pET28b plasmid using a previously reported method (30). We added a maltose binding protein tag to the C terminus of SIRT4 and Saci_0381 to promote their solubility. Sirtuin mutants of PA1197, PA1125, and TK0685 were designed by using primers carrying specific nucleotides. The corresponding PCR products were recombined using the ClonExpress II One Step Cloning kit (Vazyme, CAT. # 200550).

### Expression and purification of sirtuins

The correct plasmids (Table S1) were confirmed by PCR and then transformed into *E. coli* BL21 cells for protein expression. A single colony was picked and transferred into a 5 ml LB medium containing kanamycin overnight, and then 1:100 diluted into 500-ml LB liquid medium at 37 °C. When the absorbance at 600 nm (*A*_600_) reached approximately 0.6, the cells were transferred and induced with 0.3 mM IPTG overnight at 16 °C. Cells were harvested by centrifugation at 6000 rpm for 10 min at 4 °C and then suspended in a buffer containing 25 mM Tris–HCl (pH 8.0), 150 mM NaCl, 1 mM DTT, 10% (v/v) glycerol, and 20 mM imidazole. After cell lysis with a disruptor (Litu), cellular debris was removed by centrifugation at 13,000*g* for 30 min at 4 °C. The collected supernatants were loaded onto a 5-ml nickel resin column (GE HealthCare). After washing with ten volumes of wash buffer (25 mM Tris–HCl, 150 mM NaCl, and 30 mM imidazole; pH 8.0), the proteins were eluted (elution buffer, 25 mM Tris–HCl, 150 mM NaCl, and 500 mM imidazole; pH 8.0). The proteins were quantified, aliquoted, and stored at −80 °C (Fig. S7).

### HPLC-based sirtuin zymograph assay

The *in vitro* deacylation systems contained 50 mM Tris buffer (pH 8.0), 50 mM NaCl, 6.0 mM MgCl_2_, 1.0 mM NAD^+^, 1.0 mM acylated peptide, and 5 μM purified sirtuin. Reactions were carried out at 37 °C for 2 h in a 20-μl volume and quenched with one volume of 10% (v/v) TFA (Aladdin. CAT. # T103294) for 10 min at room temperature. The mixture was centrifuged at 13,000*g* for 10 min, and the supernatant was subjected to HPLC (Agilent Technologies) using the Aeris peptide XB-C18 column (150 × 4.6 mm, 3.6 μm; Phenomenex). The mobile phase consisted of solvent A (0.1% formic acid in HPLC-grade water) and solvent B (0.1% formic acid in HPLC-grade acetonitrile). Samples analyzed using the HPLC method were differentiated according to the acyl R-group of peptides: acetylation, lactylation, butyrylation, crotonylation, malonylation, succinylation, and glutarylation modified peptides were gradient eluted with 1 to 100% solvent B (1 min of 1% B, 9 min of 1–20% B, 0.1 min of 20–100% B, 2 min of 100% B, 0.1 min of 100–1% B, and 1% B for 3 min) over 15 min; nonanoylation and palmitoylation peptides were gradient eluted with 1 to 100% solvent B (1 min of 1% B, 19 min of 1–60% B, 0.1 min of 60–100% B, 2 min of 100% B, 0.1 min of 100–1% B, and 1% B for 3 min) over 25 min. To display a full spectrum of all modified peptides used in Figure 2*C*, peptides were gradient eluted with 1 to 100% solvent B (1 min of 1% B, 9 min of 1–20% B, 1 min of 20–40% B, 9 min of 40–60% B, 2 min of 60–100% B, 2 min of 100% B, 4 min of 100–1% B, and 1% B for 2 min) over 30 min. The column temperature was set at 25 °C, and the peptides were monitored by UV light at a wavelength of 215 nm. The deacylation activities of sirtuins were determined by calculating the peak area of the peptides as follows:

$$\text{Deacylation}\;\text{percentage}=\frac{\text{Deacylation}}{\text{Deacylation}+\text{Acylation}}\;*\;100\%$$

### AMC-based fluorogenic detection of sirtuin zymographs

The plasmids of pET28b-sirtuins were transformed into the *E. coli* BL21(Δ*cobB*) strain. At least three single colonies were picked for each sirtuin and transferred into 1 ml LB medium containing kanamycin overnight in a 96-well plate. The cultures were then 1:100 diluted into 2-ml LB liquid medium at 37 °C. When the *A*_600_ reached about 0.6, the cells were transferred to a 25 °C shaker and induced with 0.3 mM IPTG overnight. Cells were harvested by centrifugation at 4000*g* for 15 min at 4 °C and then suspended in 150 μl lysis buffer containing 25 mM Tris–HCl (pH 8.0), 1% (m/v) NP-40, and 150 mM NaCl at 37 °C for 15 min with gentle stirring. Then, the cell lysis containing sirtuin proteins was obtained by centrifugation at 4000*g* for 15 min.

The deacylation system (10 μl) containing 100 mM Tris buffer (pH 8.0), 274 mM NaCl, 2 mM MgCl_2_, 5.4 mM KCl, 2.0 mM NAD^+^, and 200 μM acylated peptide was added to 10 μl cell lysis directly. Reactions were carried out at 37 °C in a shaker (300 rpm) for 1 h. Then, an equal volume of reaction II buffer containing 2.5 mg/ml trypsin with 4 mM nicotinamide was added and left standing at 25 °C for another 1.5 h. The fluorescence intensity was detected directly at λ_ex_ = 360 nm and λ_em_ = 460 nm using a microplate reader (Synergy H1, Biotek). The deacylase activity of sirtuin was calculated by determining the production of AMC molecules, which was quantified through a standard AMC curve (built by 1 nM to 1 mM reagent grade AMC).

### Enzyme kinetics assay

Michaelis–Menten parameters were obtained by measuring conversion rates of acylated peptides using the HPLC assay in a discontinuous manner. Reactions were performed in Tris buffer (pH 8.0), where the peptide substrate (ranging from 500 μM to 2 μM) and the enzyme (100 nM) were incubated in triplicate at 37 °C in a final volume of 30 μl in microcentrifuge tubes. Then, TFA [10% (v/v)] was added to quench the reactions after 5-, 10-, and 20-min incubation times. For enzymes with high conversion rates, such as the PA1125 reaction with suc-peptides and the PA1197 reaction with ac-peptides, the incubation times were 1, 2, and 3 min. For enzymes with very low conversion rates, such as the PA1125 reaction with ac-peptides, the PA1197 reaction with suc-peptides, and the TK0685 reaction with ac-peptides, the incubation times were 30, 45, and 60 min. Samples were then centrifuged at 13,000*g* for 10 min, and the supernatants were subjected to HPLC analysis as described above. If the calculated values showed a linear trend, the obtained initial rates (ν_0_) were adjusted to the Michaelis–Menten equation to determine *K*_M_ and *k*_cat_ values for each substrate concentration using GraphPad Prism 9 (https://www.graphpad.com/).

### Data processing and database searches

A total of 101 sirtuin amino acid sequences from 44 organisms (Table S6) were retrieved from the KEGG database (https://www.kegg.jp/). The phylogenetic tree was built by using all 101 sirtuins or the 31 representative sirtuins with the maximum likelihood method and Jones-Taylor-Thornton matrix-based model (34). Initial tree(s) for the heuristic search were obtained automatically by applying Neighbor-Joining and BioNJ algorithms to a matrix of pairwise distances estimated using the Jones-Taylor-Thornton model, and then selecting the topology with superior log likelihood value. The tree is drawn to scale, with branch lengths measured in the number of substitutions per site. Evolutionary analyses were conducted in MEGA X (35).

The open-access AlphaFold2 Colab platform (36) was used to predict the protein structures of PA1125, PA1197, TK0685, Saci_0381, and their mutants. The platform is available at https://colab.research.google.com/github/sokrypton/ColabFold/blob/main/AlphaFold2.ipynb. The amino acid sequence of each sirtuin was used as input, and automatic homology alignment was done by MMseqs2 (37) and/or HHsearch (38).

### Statistics and reproducibility

All experiments were repeated at least three times independently. No statistical method was used to predetermine sample size and no data were excluded from the analyses. All statistical analyses were performed using the GraphPad Prism 5.0 program. Statistically significant differences were determined using Welch’s or Student’s *t* test. Data are presented as means ± standard error of the mean (SEM) with the number of experimental replicates (n) provided in the figures or corresponding figure legends. *p* <0.05 was considered significant.

## Data availability

Data supporting the findings of this study are available within the main manuscript and the Supplementary information. Relevant raw data are available from the corresponding author upon reasonable request.

## Supporting information

This article contains supporting information.

## Conflict of interest

The authors declare that they have no conflicts of interest with the contents of this article.
