# Supplementary material for: Zymograph profiling reveals a divergent evolution of sirtuin that may originate from class III enzymes
Source: J Biol Chem. 2023 Oct 12;299(11):105339. doi: 10.1016/j.jbc.2023.105339 (PMC10652111; doi:10.1016/j.jbc.2023.105339)
Supplement: Supporting Figures S1–S7 and Tables S1–S6 [file mmc1.pdf]

SUPPLEMENTARY INFORMATION

Zymograph profiling reveals a divergent evolution of sirtuin that may originate from Class III enzymes

Yujiao Yang, Siwei Zou, Kezhu Cai, Ningning Li, Zhongyue Li, Wei Tan, Wei Lin, Guo-Ping Zhao, Wei Zhao

Supplementary Figures 1-7

Supplementary Tables 1-6

SUPPLEMENTARY FIGURES

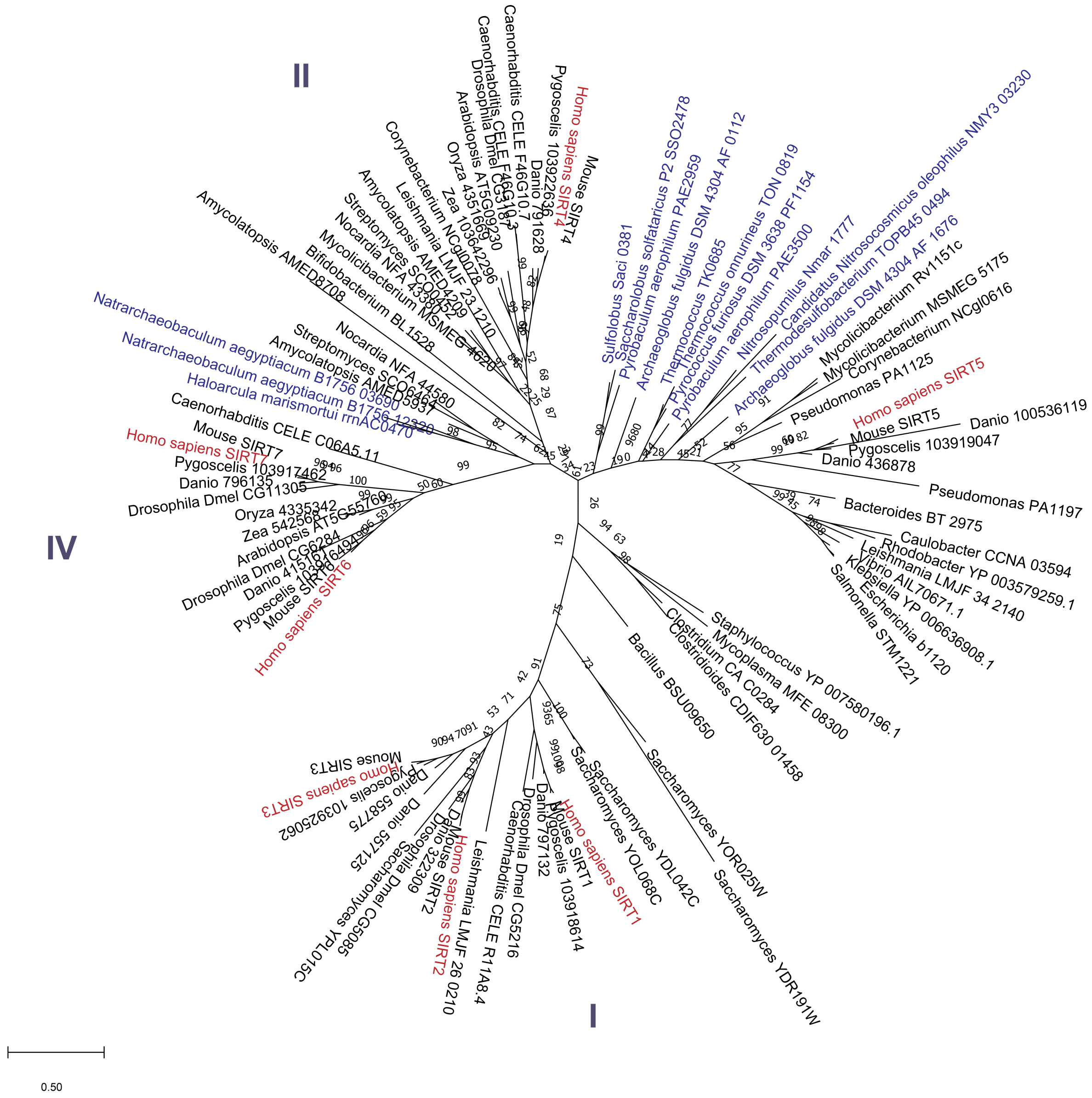

Supplementary Figure 1. Phylogenetic analysis suggests that sirtuins could be grouped into four major Classes. The phylogenetic tree was built by the amino acid sequences of 101 sirtuins that were selected from the representative species in three domains of life. The evolutionary history was inferred by using the Maximum Likelihood method and JTT matrix-based model. The tree with the highest log likelihood (-21169.52) is shown. Initial tree(s) for the heuristic search were obtained automatically by applying Neighbor-Join and BioNJ algorithms to a matrix of pairwise distances estimated using the JTT model and then selecting the topology

with superior log likelihood value. The tree is drawn to scale, with branch lengths measured in the number of substitutions per site. All positions with less than 95% site coverage were eliminated, *i.e.,* fewer than 5% alignment gaps, missing data, and ambiguous bases were allowed at any position (partial deletion option). The sirtuin amino acid sequences were retrieved from the KEGG database (https://www.kegg.jp/). Evolutionary analyses were conducted in MEGA X. Red, human sirtuins; blue, archaeal sirtuins.

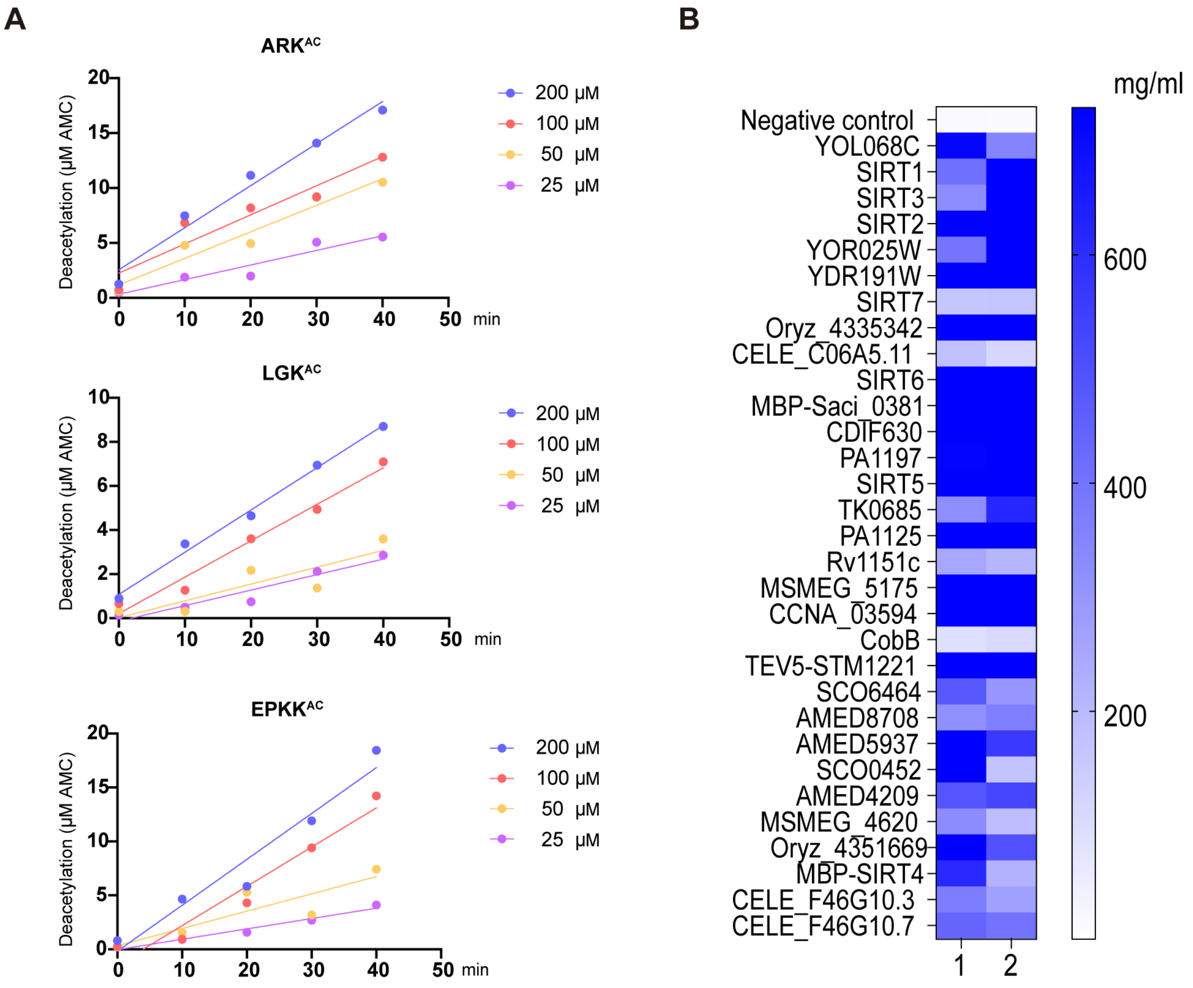

Supplementary Figure 2. Evaluation of the reliability of AMC-based fluorogenic detection method. A, The AMC fluorescence signal increases in direct proportion to the amount of deacylated peptide substrate. The deacylation reactions were performed using *Salmonella* sirtuin (STM1221) against the acetylated peptides using three different sequences (ARK^{AC}, LGK^{AC} and EPKK^{AC}), respectively. The reaction mixture contained 10 μM STM1221, 1 mM NAD^{+}, and different concentrations of acetylated peptides (25, 50, 100, 200 μM), respectively, was incubated at 37 °C for 2 hours. The results showed a significant correlation between the AMC fluorescence signal and the amounts of deacylated peptide substrates. **B,** The quantification of sirtuin expression levels in *E. coli* BL21(Δ*cobB*). The concentrations of His-tagged sirtuin proteins in cytoplasmic fractions were quantitatively analyzed twice using an

ELISA kit (L00436, Genscript) according to the standard method, with blank plasmid as the negative control and standard sample in the kit as the positive control. The standard curve was plotted using a Four Parameter Logistic (4PL) curve fitting model in GraphPad Prism 9.

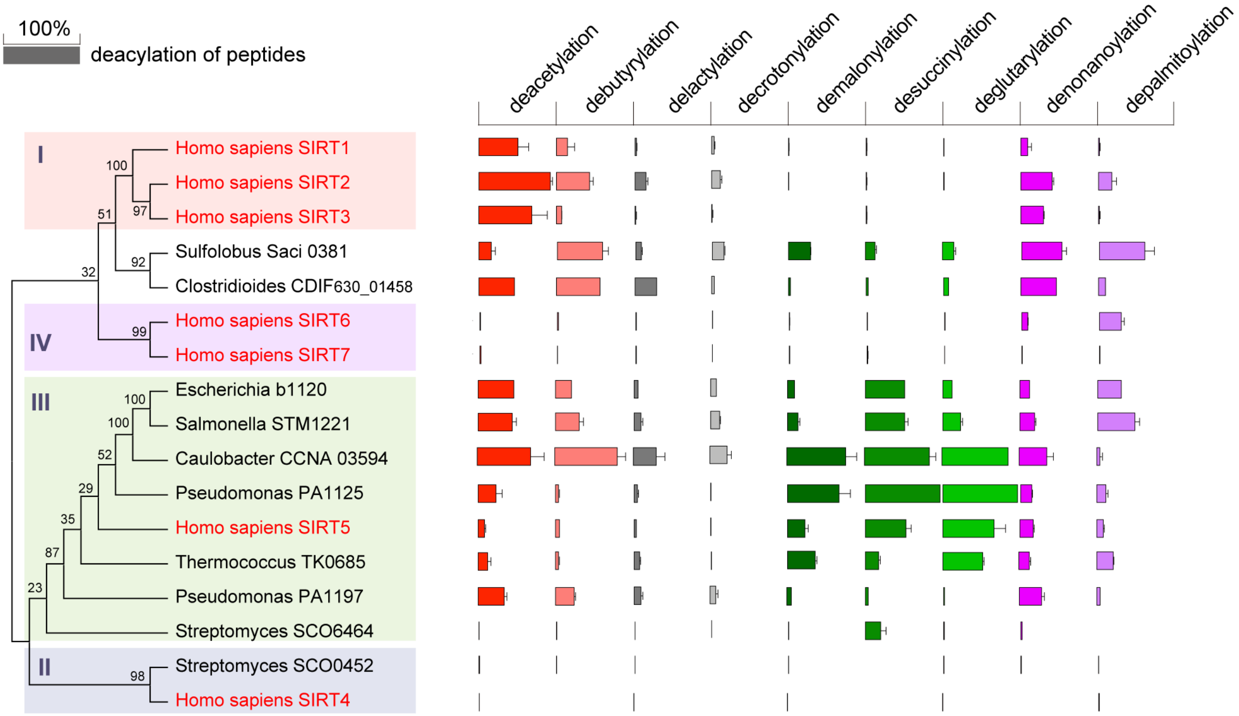

Supplementary Figure 3. Global activity profiling using HPLC confirms undifferentiated zymographs of Class III sirtuins. The phylogenetic tree (left panel) was built based on 17 amino acid sequences of model sirtuins. The corresponding zymographs of sirtuins were profiled on the right panel. The deacylation reactions were performed using 9 different acylated H3K27 (ATKAARK*SAPATG) peptides as described in *Experimental Procedures*. The reaction mixture containing 5 µM purified sirtuin, 1 mM peptide, and 1 mM NAD^+ was incubated at 37 °C for 2 hours, and the deacylation activity was determined by calculating the ratio of deacylated peptides. Data are represented as means ± SEM (n = 3).

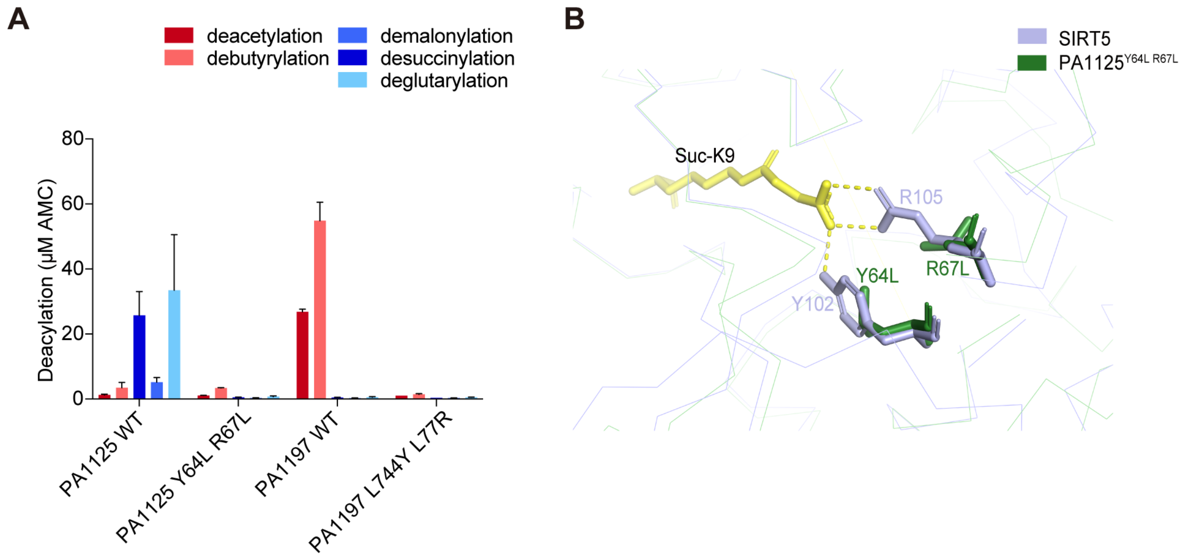

Supplementary Figure 4. Site-directed mutagenesis reveals the key sites that are involved in the regulation of *Pseudomonas* sirtuin differentiation. A, The determination of deacylase activities after site-directed mutagenesis of PA1125 and PA1197. The reaction mixture containing 10 μM sirtuin, 200 μM peptide, and 1 mM NAD^+ was incubated at 37 °C for 60 minutes, followed by the treatment using 2.5 mg/mL trypsin at 25 °C for another 90 minutes. The fluorescent results were shown as the production rates of AMC molecules. Data are represented as means ± SEM (n = 3). **B,** Structure analyses demonstrate the key sites that are involved in the regulation of desuccinylase activity in sirtuins. When the corresponding sites mutated to L64 and L67, the interactions of PA1125 with the negatively charged-chain peptides could be lost. The structure of PA1125 was modeled by Alphafold2, and was overlapped with SIRT5 (PDB entry 3RIG). Sirtuin mutations were performed using PyMOL.

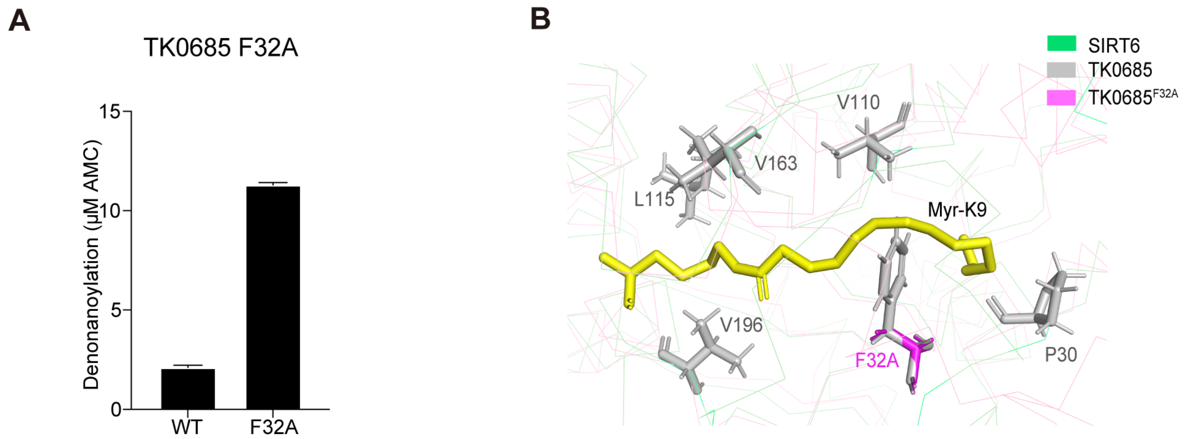

Supplementary Figure 5. Site-directed mutagenesis reveals the key sites that are involved in the regulation of archaeal sirtuin differentiation. A, The determination of denonanoylase activity after site-directed mutagenesis of TK0685. The reaction mixture containing 10 μM sirtuin, 200 μM peptide, and 1 mM NAD^+ was incubated at 37 °C for 60 minutes, followed by the treatment using 2.5 mg/mL trypsin at 25 °C for another 90 minutes. The fluorescent results were shown as the production rates of AMC molecules. Data are represented as means ± SEM (n = 3). **B.** Structure analyses demonstrate the key site that is involved in the regulation of denonanoylase activity in TK0685. The catalytic pocket of TK0685 may be enlarged after F32A mutation, thereby its denononylase activity could be enhanced. The structure of TK0685 was modeled by Alphafold2, and was overlapped with SIRT6 (PDB entry 3ZG6). Sirtuin mutation at a single site were performed using PyMOL.

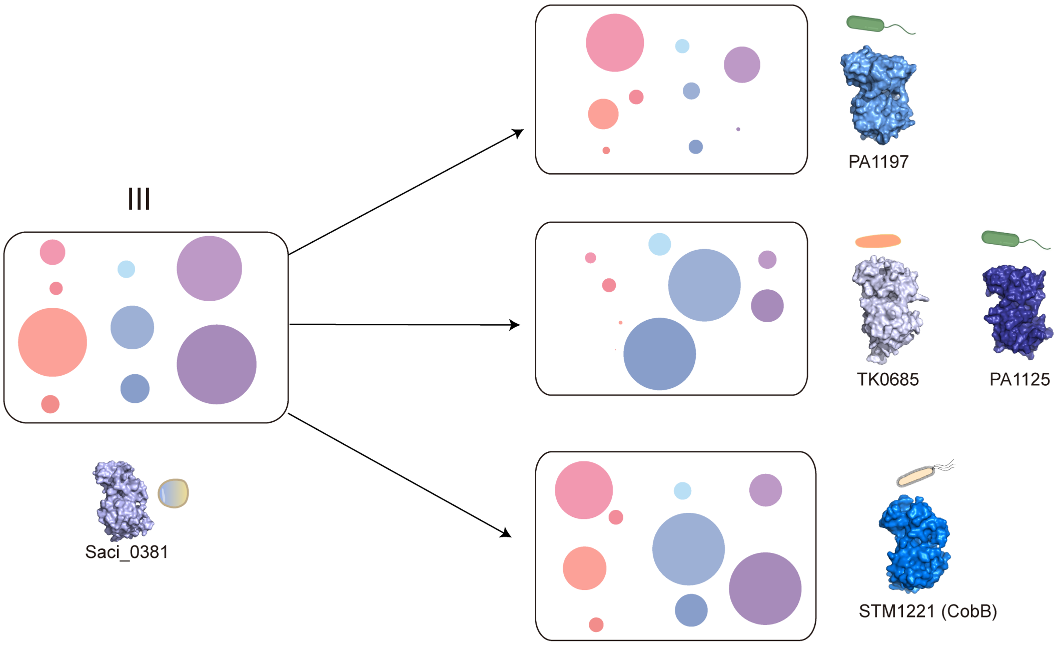

Supplementary Figure 6. The model of initially differentiations of Class III sirtuins. The sub-group of sirtuins with different zymographs were observed in Class III enzymes. Circles with different colors and sizes represent the differences in enzyme activity categories and capacities, respectively. The red color indicates the short-chain deacylase activity, the violet color indicates the long-chain deacylase activity, and the blue color indicates the negatively charged-chain deacylase activity. The size of circles is quantitated from data in figure 2, 4 and 5. The displayed structures of sirtuins (TK0685, Saci_0381, PA1125 and PA1197) were generated by Alphafold2 modeling. CobB: 1S5P. All structures are represented by surface patterns using PyMOL (https://pymol.org).

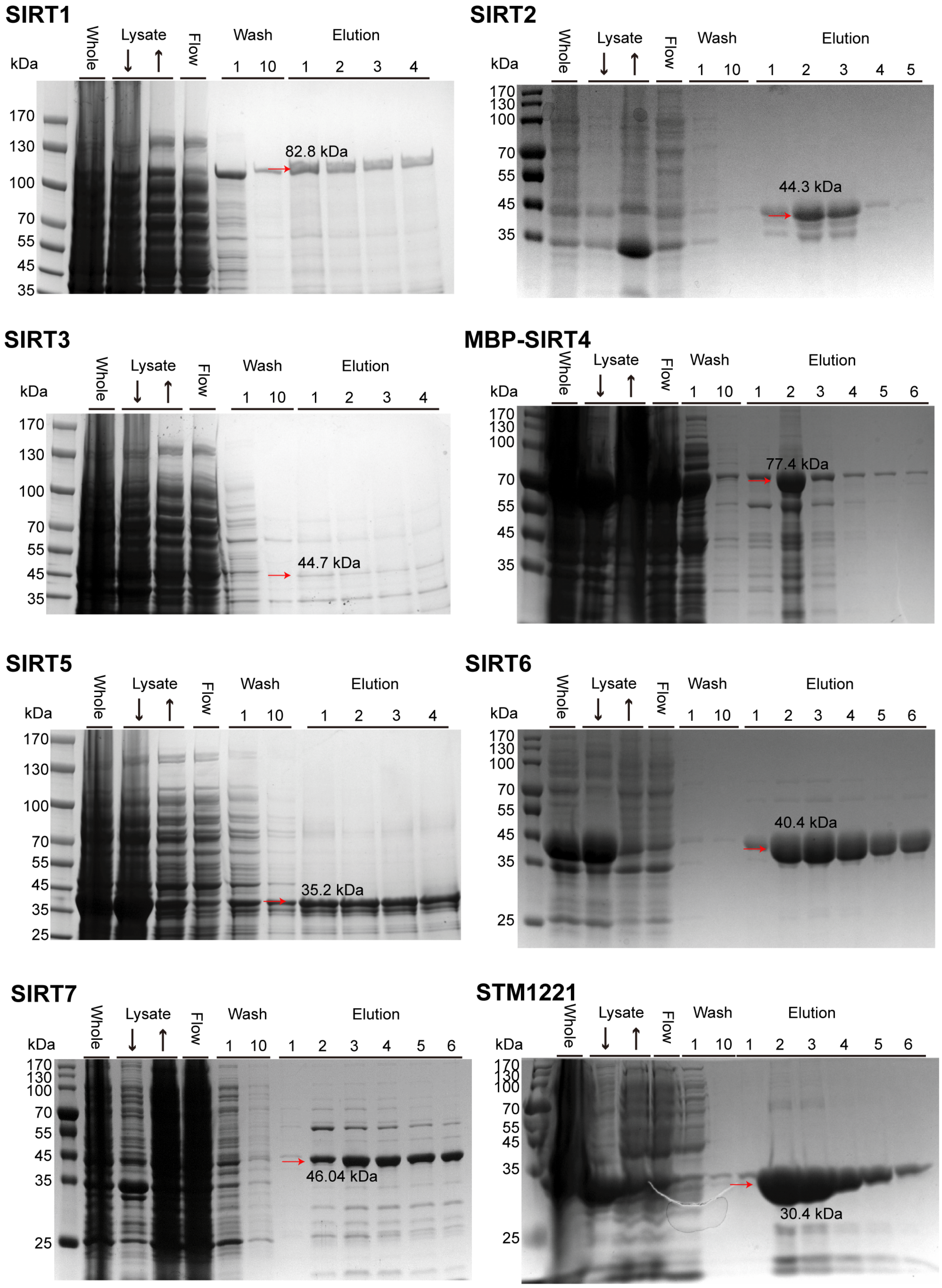
SIRT1
Whole
Lysate
Flow
Wash
Elution
kDa
1 10 1 2 3 4
170
130
100
70
55
45
35
82.8 kDa
SIRT2
Whole
Lysate
Flow
Wash
Elution
kDa
1 10 1 2 3 4 5
170
130
100
70
55
45
35
44.3 kDa
SIRT3
Whole
Lysate
Flow
Wash
Elution
kDa
1 10 1 2 3 4
170
130
100
70
55
45
35
44.7 kDa
MBP-SIRT4
Whole
Lysate
Flow
Wash
Elution
kDa
1 10 1 2 3 4 5 6
170
130
100
70
55
45
35
77.4 kDa
SIRT5
Whole
Lysate
Flow
Wash
Elution
kDa
1 10 1 2 3 4
170
130
100
70
55
45
35
25
35.2 kDa
SIRT6
Whole
Lysate
Flow
Wash
Elution
kDa
1 10 1 2 3 4 5 6
170
130
100
70
55
45
35
25
40.4 kDa
SIRT7
Whole
Lysate
Flow
Wash
Elution
kDa
1 10 1 2 3 4 5 6
170
130
100
70
55
45
35
25
46.04 kDa
STM1221
Whole
Lysate
Flow
Wash
Elution
kDa
1 10 1 2 3 4 5 6
170
130
100
70
55
45
35
25
30.4 kDa

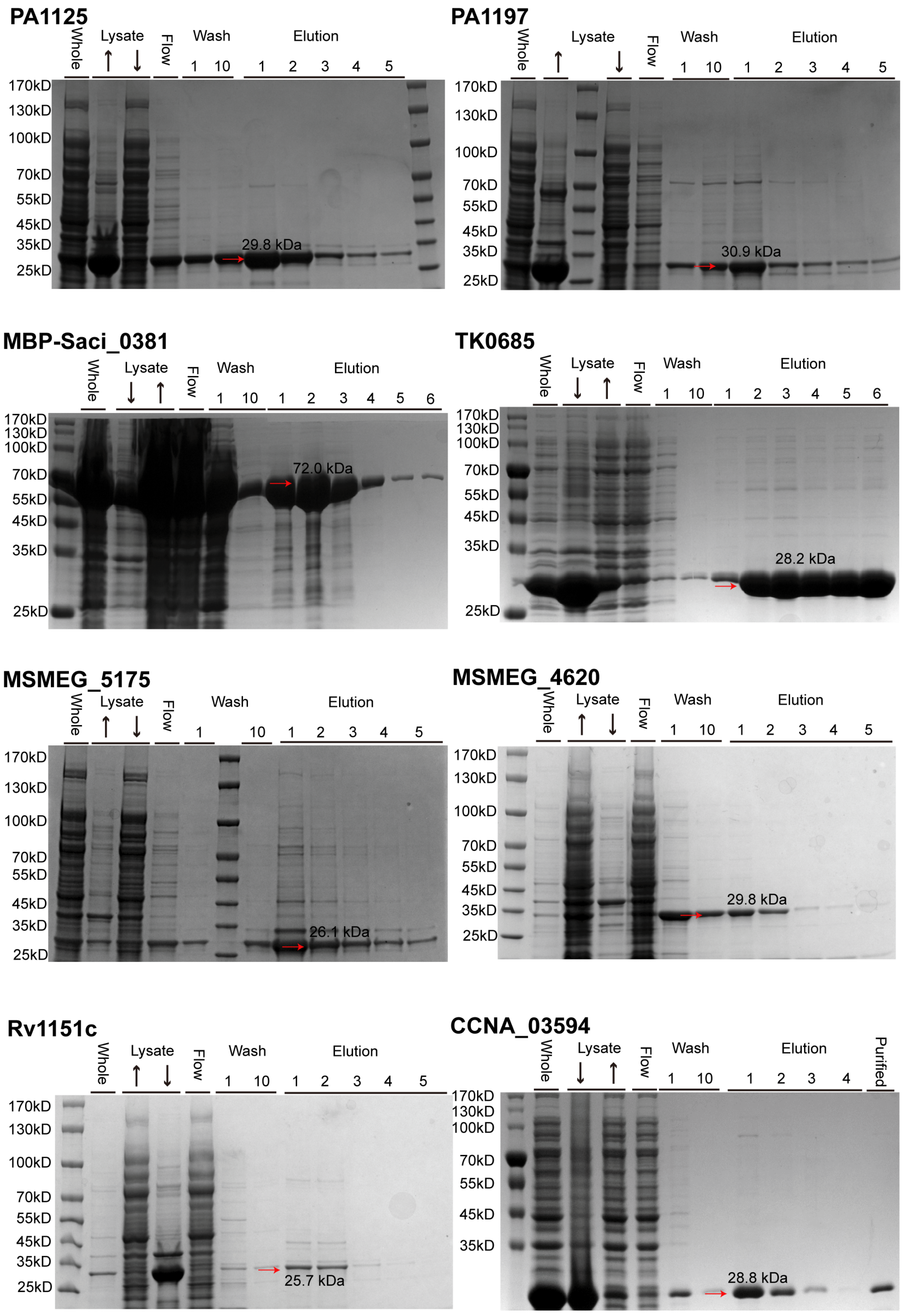

PA1125
Whole
Lysate
Flow
Wash
1 10
Elution
1 2 3 4 5
170kD
130kD
100kD
70kD
55kD
45kD
35kD
25kD
29.8 kDa
PA1197
Whole
Lysate
Flow
Wash
1 10
Elution
1 2 3 4 5
170kD
130kD
100kD
70kD
55kD
45kD
35kD
25kD
30.9 kDa
MBP-Saci_0381
Whole
Lysate
Flow
Wash
1 10
Elution
1 2 3 4 5 6
170kD
130kD
100kD
70kD
55kD
45kD
35kD
25kD
72.0 kDa
TK0685
Whole
Lysate
Flow
Wash
1 10
Elution
1 2 3 4 5 6
170kD
130kD
100kD
70kD
55kD
45kD
35kD
25kD
28.2 kDa
MSMEG_5175
Whole
Lysate
Flow
Wash
1 10
Elution
1 2 3 4 5
170kD
130kD
100kD
70kD
55kD
45kD
35kD
25kD
26.1 kDa
MSMEG_4620
Whole
Lysate
Flow
Wash
1 10
Elution
1 2 3 4 5
170kD
130kD
100kD
70kD
55kD
45kD
35kD
25kD
29.8 kDa
Rv1151c
Whole
Lysate
Flow
Wash
1 10
Elution
1 2 3 4 5
170kD
130kD
100kD
70kD
55kD
45kD
35kD
25kD
25.7 kDa
CCNA_03594
Whole
Lysate
Flow
Wash
1 10
Elution
1 2 3 4
Purified
170kD
130kD
100kD
70kD
55kD
45kD
35kD
28.8 kDa

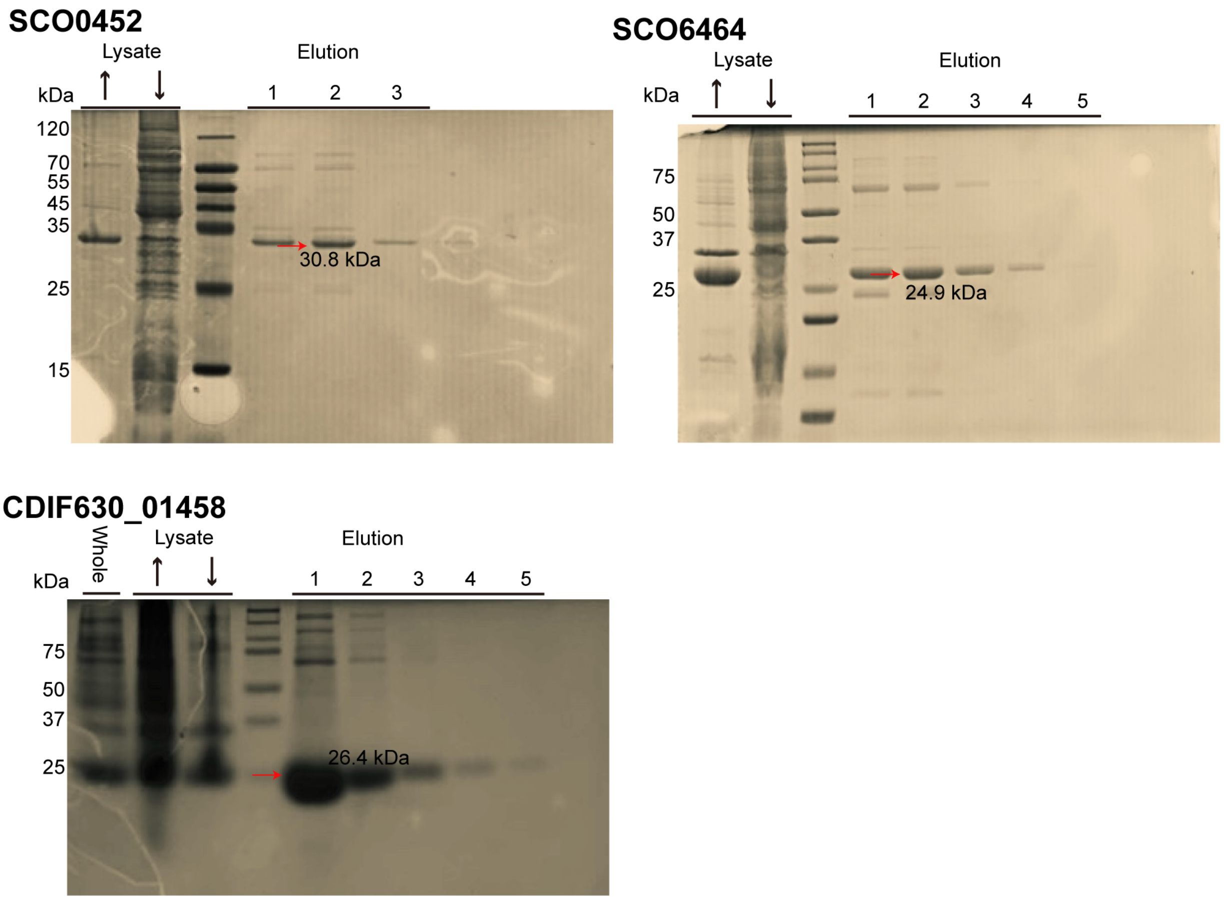

Supplementary Figure 7. SDS-PAGE analyses of the recombinant sirtuins used in this study. Protein samples during the purification process are shown on PAGE, including the whole cell lysate after expressing sirtuins, the supernatant (up arrow), the inclusion bodies (down arrow), the flow-through fraction, the wash-out fraction and the pure sirtuins eluted from the Ni-NTA resin by a 500 mM imidazole. The red arrows represent the protein bands corresponding to the molecular masses predicted for each sirtuin protein. All the proteins were collected and stored at -80 °C before use. Concentrations of purified proteins were determined using a Bradford protein assay kit.

SUPPLEMENTARY TABLES

Supplementary Table 1. Plasmids used in this study.

Plasmid	Plasmid information[a]	Source or reference
pYJ001	Kan^R; pET28b-YOL068C	This study
pYJ002	Kan^R; pET28b-SIRT1	This study
pYJ003	Kan^R; pET28b-SIRT3	This study
pYJ004	Kan^R; pET28b-SIRT2	This study
pYJ005	Kan^R; pET28b-YOR025W	This study
pYJ006	Kan^R; pET28b-YDR191W	This study
pYJ007	Kan^R; pET28b-SIRT7	This study
pYJ008	Kan^R; pET28b-4335342	This study
pYJ009	Kan^R; pET28b-CELE_C06A5.11	This study
pYJ010	Kan^R; pET28b-SIRT6	This study
pYJ011	Kan^R; pET28b-MBP-Saci_0381	This study
pYJ012	Kan^R; pET28b-CDIF630	This study
pYJ013	Kan^R; pET28b-PA1197	This study
pYJ014	Kan^R; pET28b-SIRT5	This study
pYJ015	Kan^R; pET28b-TK0685	This study
pYJ016	Kan^R; pET28b-PA1125	This study
pYJ017	Kan^R; pET28b-Rv1151c	This study
pYJ018	Kan^R; pET28b-MSMEG_5175	This study
pYJ019	Kan^R; pET28b-CCNA_03594	This study
pYJ020	Kan^R; pET28b-CobB	This study
pYJ021	Kan^R; pET28b-STM1221	This study
pYJ022	Kan^R; pET28b-SCO6464	This study
pYJ023	Kan^R; pET28b-AMED8708	This study
pYJ024	Kan^R; pET28b-AMED5937	This study
pYJ025	Kan^R; pET28b-SCO0452	This study
pYJ026	Kan^R; pET28b-AMED4209	This study
pYJ027	Kan^R; pET28b-MSMEG_4620	This study
pYJ028	Kan^R; pET28b-4351669	This study
pYJ029	Kan^R; pET28b-MBP-SIRT4	This study
pYJ030	Kan^R; pET28b-CELE_F46G10.3	This study
pYJ031	Kan^R; pET28b-CELE_F46G10.7	This study
pYJ032	Kan^R; pET28b-PA1125-Y64L-R67L	This study
pYJ033	Kan^R; pET28b-PA1197-L74Y-L77R	This study
pYJ034	Kan^R; pET28b-TK0685-F32A	This study
pYJ035	Amp^R; pET32-MBP	Xian'en Zhang lab

[a]Abbreviations: Kan, kanamycin; Amp, ampicillin; R, resistance.

Supplementary Table 2. Bacterial strains used in this study.

Strains	Description	Construction, source, or reference
G2466	*S. enterica* wild-type strain	This Lab
M145	*S. coelicolor* wild-type strain	This Lab
U32	*A. mediterranei* wild-type strain	This Lab
PAO1	*P. aeruginosa* wild-type strain	This Lab
mc(2)155	*M. smegmatis* wild-type strain	This Lab
MG1655	*E. coli* wild-type strain	This Lab
NA1000	*C. crescentus* wild-type strain	Lucy Shapiro lab
DH5α	Bacterial cloning strain	Novagen
BL21(DE3)	Bacterial expression strain	Novagen
BL21Δ*cobB*	Bacterial expression strain	This Lab
YJ001	BL21Δ*cobB*, pET28b-YOL068C	Transformation of pYJ001 into BL21Δ*cobB*
YJ002	BL21Δ*cobB*, pET28b-SIRT1	Transformation of pYJ002 into BL21Δ*cobB*
YJ003	BL21Δ*cobB*, pET28b-SIRT3	Transformation of pYJ003 into BL21Δ*cobB*
YJ004	BL21Δ*cobB*, pET28b-SIRT2	Transformation of pYJ004 into BL21Δ*cobB*
YJ005	BL21Δ*cobB*, pET28b-YOR025W	Transformation of pYJ005 into BL21Δ*cobB*
YJ006	BL21Δ*cobB*, pET28b-YDR191W	Transformation of pYJ006 into BL21Δ*cobB*
YJ007	BL21Δ*cobB*, pET28b-SIRT7	Transformation of pYJ007 into BL21Δ*cobB*
YJ008	BL21Δ*cobB*, pET28b-4335342	Transformation of pYJ008 into BL21Δ*cobB*
YJ009	BL21Δ*cobB*, pET28b-CELE_C06A5.11	Transformation of pYJ009 into BL21Δ*cobB*
YJ010	BL21Δ*cobB*, pET28b-SIRT6	Transformation of pYJ010 into BL21Δ*cobB*
YJ011	BL21Δ*cobB*, pET28b-MBP-Saci_0381	Transformation of pYJ011 into BL21Δ*cobB*
YJ012	BL21Δ*cobB*, pET28b-CDIF630	Transformation of pYJ012 into BL21Δ*cobB*
YJ013	BL21Δ*cobB*, pET28b-PA1197	Transformation of pYJ013 into BL21Δ*cobB*
YJ014	BL21Δ*cobB*, pET28b-SIRT5	Transformation of pYJ014 into BL21Δ*cobB*
YJ015	BL21Δ*cobB*, pET28b-TK0685	Transformation of pYJ015 into BL21Δ*cobB*
YJ016	BL21Δ*cobB*, pET28b-PA1125	Transformation of pYJ016 into BL21Δ*cobB*
YJ017	BL21Δ*cobB*, pET28b-Rv1151c	Transformation of pYJ017 into BL21Δ*cobB*
YJ018	BL21Δ*cobB*, pET28b-MSMEG_5175	Transformation of pYJ018 into BL21Δ*cobB*
YJ019	BL21Δ*cobB*, pET28b-CCNA_03594	Transformation of pYJ019 into BL21Δ*cobB*
YJ020	BL21Δ*cobB*, pET28b-CobB	Transformation of pYJ020 into BL21Δ*cobB*
YJ021	BL21Δ*cobB*, pET28b-STM1221	Transformation of pYJ021 into BL21Δ*cobB*
YJ022	BL21Δ*cobB*, pET28b-SCO6464	Transformation of pYJ022 into BL21Δ*cobB*
YJ023	BL21Δ*cobB*, pET28b-AMED8708	Transformation of pYJ023 into BL21Δ*cobB*
YJ024	BL21Δ*cobB*, pET28b-AMED5937	Transformation of pYJ024 into BL21Δ*cobB*
YJ025	BL21Δ*cobB*, pET28b-SCO0452	Transformation of pYJ025 into BL21Δ*cobB*
YJ026	BL21Δ*cobB*, pET28b-AMED4209	Transformation of pYJ026 into BL21Δ*cobB*
YJ027	BL21Δ*cobB*, pET28b-MSMEG_4620	Transformation of pYJ027 into BL21Δ*cobB*
YJ028	BL21Δ*cobB*, pET28b-4351669	Transformation of pYJ028 into BL21Δ*cobB*
YJ029	BL21Δ*cobB*, pET28b-MBP-SIRT4	Transformation of pYJ029 into BL21Δ*cobB*
YJ030	BL21Δ*cobB*, pET28b-CELE_F46G10.3	Transformation of pYJ030 into BL21Δ*cobB*

YJ031	BL21Δ*cobB*, pET28b-CELE_F46G10.7	Transformation of pYJ031 into BL21Δ*cobB*
YJ032	BL21Δ*cobB*, pET28b-PA1125-Y64L-R67L	Transformation of pYJ032 into BL21Δ*cobB*
YJ033	BL21Δ*cobB*, pET28b-PA1197-L74Y-L77R	Transformation of pYJ033 into BL21Δ*cobB*
YJ034	BL21Δ*cobB*, pET28b-TK0685-F32A	Transformation of pYJ034 into BL21Δ*cobB*

Supplementary Table 3. Oligonucleotides used in this study.

Primer	Sequence	Plasmids
NLP-001	taatacgactcactataggg	pYJ001-034
NLP-002	tgctagttattgctcagcgg	
NLP-003	gctagcgccctgaaaatacagg	
NLP-004	ggatccgcggccgctgagca	
NLP-005	cctgtattttcagggcgctagcatgaaaatcgaagaaggtaa	pYJ011
NLP-006	cccatttgctgtccaccagtcatgctagccatatggctgccgcgcggcaccaggctaccactgcca ccagtctgcgcgtctttcagggct	
NLP-007	gactggtggacagcaaatgggtcgcggatccatgctgccgtactctgttaac	
NLP-008	tgctcagcggccgcggatccttagctagaacggtgcagag	
NLP-009	ctctgcaccgttctagctaaggatccgcggccgctgagca	
NLP-010	ttaccttcttcgattttcatgctagcgccctgaaaatacagg	
NLP-011	cctgtattttcagggcgctagcatgaaaatcgaagaaggtaa	pYJ029
NLP-012	cccatttgctgtccaccagtcatgctagccatatggctgccgcgcggcaccaggctaccactgcca ccagtctgcgcgtctttcagggct	
NLP-013	gactggtggacagcaaatgggtcgcggatccatgaagatgagctttgcgtt	
NLP-014	tgctcagcggccgcggatcctcagcatgggtctatcaaag	
NLP-015	ctttgatagacccatgctgaggatccgcggccgctgagca	
NLP-016	ttaccttcttcgattttcatgctagcgccctgaaaatacagg	
NLP-017	cctgtattttcagggcgctagcatggacagccacagcccgat	pYJ013
NLP-018	tgctcagcggccgcggatcc ttatcgataaatgtgactta	
NLP-019	taagtcacatttatcgataaggatccgcggccgctgagca	
NLP-020	atcgggctgtggctgtccatgctagcgccctgaaaatacagg	
NLP-021	cctgtattttcagggcgctagcatgcgcgctgtcgtcgaact	pYJ016
NLP-022	tgctcagcggccgcggatcctcagccgggaaacgcccggc	
NLP-023	gccgggcgtttcccggctgaggatccgcggccgctgagca	
NLP-024	agttcgacgacagcgcgcatgctagcgccctgaaaatacagg	
NLP-025	cctgtattttcagggcgctagcgtgcaagttactgtgctcag	pYJ018
NLP-026	tgctcagcggccgcggatcctcaggccgagcggttgagca	
NLP-027	tgctcaaccgctcggcctgaggatccgcggccgctgagca	
NLP-028	ctgagcacagtaacttgcacgctagcgccctgaaaatacagg	
NLP-029	cctgtattttcagggcgctagcatgcgagggatcatgaaagt	pYJ019
NLP-030	tgctcagcggccgcggatccctagagcctcgccagcaacc	
NLP-031	ggttgctggcgaggctctagggatccgcggccgctgagca	
NLP-032	actttcatgatccctcgcatgctagcgccctgaaaatacagg	
NLP-033	cctgtattttcagggcgctagcatgctgtcgcgtcggggtca	pYJ020
NLP-034	tgctcagcggccgcggatcctcaggcaatgcttcccgctt	
NLP-035	aagcgggaagcattgcctgaggatccgcggccgctgagca	
NLP-036	tgaccccgacgcgacagcatgctagcgccctgaaaatacagg	
NLP-037	cctgtattttcagggcgctagc atgcagtcgcgtcggtttca	pYJ021
NLP-038	tgctcagcggccgcggatcc ctacagccctttcaggaatt	

NLP-039	aattcctgaaagggctgtagggatccgcggccgctgagca	
NLP-040	tgaaaccgacgcgactgcatgctagcgccctgaaaatacagg	
NLP-041	cctgtattttcagggcgctagcatgaccggcaagcctctcgt	pYJ022
NLP-042	tgctcagcggccgcggatcctcagcccagcccgcgcagca	
NLP-043	tgctgcgcgggctgggctgaggatccgcggccgctgagca	
NLP-044	acgagaggcttgccggtcatgctagcgccctgaaaatacagg	
NLP-045	cctgtattttcagggcgctagcgtgcgtgagctcgccgcgaa	pYJ023
NLP-046	tgctcagcggccgcggatcctcaggacgcgggattccgtg	
NLP-047	cacggaatcccgcgtcctgaggatccgcggccgctgagca	
NLP-048	ttcgcggcgagctcacgcacgctagcgccctgaaaatacagg	
NLP-049	cctgtattttcagggcgctagcatggacggcaagctcatcga	pYJ024
NLP-050	tgctcagcggccgcggatcctcaccggttcaccgcggcgc	
NLP-051	gcgccgcggtgaaccggtgaggatccgcggccgctgagca	
NLP-052	tcgatgagcttgccgtccatgctagcgccctgaaaatacagg	
NLP-053	cctgtattttcagggcgctagcatgcgcatgcgccccactct	pYJ025
NLP-054	tgctcagcggccgcggatcctcaggccgtcgccgcgtccc	
NLP-055	gggacgcggcgacggcctgaggatccgcggccgctgagca	
NLP-056	agagtggggcgcatgcgcatgctagcgccctgaaaatacagg	
NLP-057	cctgtattttcagggcgctagcgtgcggacccggcccaccct	pYJ026
NLP-058	tgctcagcggccgcggatcctcacgcggttcggccgcgat	
NLP-059	atcgcggccgaaccgcgtgaggatccgcggccgctgagca	
NLP-060	agggtgggccgggtccgcacgctagcgccctgaaaatacagg	
NLP-061	cctgtattttcagggcgctagcatggatgcccccgaactcgt	pYJ027
NLP-062	tgctcagcggccgcggatccctatccgggcgatgccgtgt	
NLP-063	acacggcatcgcccggatagggatccgcggccgctgagca	
NLP-064	acgagttcgggggcatccatgctagcgccctgaaaatacagg	
NLP-065	gctggctcgagtggctccgcctgaaggtgctgggtgtcc	pYJ032
NLP-066	gcggagccactcgagccagccccagaccagcgcgggatc	
NLP-067	gtactacgcggaacgcggcaaggcttgcctggcggcccg	pYJ033
NLP-068	tgccgcgttccgcgtagtacttccagcacagcgccggat	
NLP-069	tccgaccgcccgtggtcgtaacggtctgtggaaaacc	pYJ034
NLP-070	gaccacgggcggtcggaataccgctttcagcagaaat	

Supplementary Table 4. Peptides used in this study.

Peptide	Sequence	Modification
P000	ATKAARKSAPATG	None
P001	ATKAARK^{AC}SAPATG	Acetylation
P002	ATKAARK^{LAC}SAPATG	Lactylation
P003	ATKAARK^{CRO}SAPATG	Crotonylation
P004	ATKAARK^{BUT}SAPATG	Butyrylation
P005	ATKAARK^{MAL}SAPATG	Malonylation
P006	ATKAARK^{SUC}SAPATG	Succinylation
P007	ATKAARK^{GLU}SAPATG	Glutarylation
P008	ATKAARK^{NON}SAPATG	Nonanoylation
P009	ATKAARK^{PAL}SAPATG	Palmitoylation
P010	ARK^{AC}	Acetylation
P011	ARK^{LAC}	Lactylation
P012	ARK^{BUT}	Butyrylation
P013	ARK^{MAL}	Malonylation
P014	ARK^{SUC}	Succinylation
P015	ARK^{GLU}	Glutarylation
P016	ARK^{NON}	Nonanoylation
P017	ARK^{MYR}	Myristoylation
P018	ARK^{PAL}	Palmitoylation
P019	LGK^{AC}	Acetylation
P020	LGK^{LAC}	Lactylation
P021	LGK^{BUT}	Butyrylation
P022	LGK^{MAL}	Malonylation
P023	LGK^{SUC}	Succinylation
P024	LGK^{GLU}	Glutarylation
P025	LGK^{NON}	Nonanoylation
P026	LGK^{MYR}	Myristoylation
P027	LGK^{PAL}	Palmitoylation
P028	EPKK^{AC}	Acetylation

Supplementary Table 5. Genes synthesized in this study.

Sirtuin	Gene ID*	Sequence
CDIF630_01458	4914019	atggatgctaattctctgaaagacttaattgctaaccataataacattgttttcttcggtggcgcgggcgtgagcact<br>gaatctaatatcccggacttccgtagctctaccggcctgttctctcagaaactgaacaaacagttcaccgccgaa<br>cagctggttagccacaccttcttcgttcgttacccggaagagttcttcgaattttacaaagataaactgatctacccg<br>aacgcgaaaccgaacaacgcgcacatcgcgctggcgaaactggaagaaatgggtaaactgaaagcggttatc<br>acccagaacatcgatggcctgcaccagatggcgggtagcaaaaacgttctggaactgcacggcagcgttcac<br>cgtaactactgcaccaaatgcggtaaattctttgatctggagtctatgctgaacctgggtggtaacatcccgtactg<br>cgataactgcggctctatcgttaaaccggatgttgttctgtacgaagaagcgctggattctgatgttatcaccaaaa<br>ccatctctgcgatcagcaacgcggatctgctgatcatcggtggtaccagcctggcggtttacccggcggcgtctt<br>tcatcgattactacaaaggtgattacatcgcgctgatcaacaaagcgaacaccgtttatgataaaagcgcgagcc<br>tggttatcaacaaaccgatcggtgaagttctgtacgaagcggttctgcgtcagatctag
CELE_F46G10.7	181455	atggctcgtaaatatgttccgcacaccaccgaactgtgcgaaaactctctgaaaaaattcaaatctctggtgggta<br>ccgtggataagctgctgatcatcaccggcgcgggcatctctaccgaaagcggtatccccgattatcgttccaaa<br>gatgttggtctgtacaccaaaaccgcgctggaaccgatctactttcaggatttcatgaaatctaaaaaatgccgtc<br>agcgttactggtctcgttcttatctgaactggccgcgcttcgcacaggcgctgccgaacttcaaccattacgcgct<br>gtccaaatgggaagcagctaacaaattccactggctgatcacccagaacgttgacggcctgcacctgaaagca<br>ggttccaaaatgatcaccgaactgcacggtaacgcgctgcaggttaaatgtaccagctgcgaatacattgaaac<br>ccgtcagacctaccaggaccgtctgaactatgctaacccaggtttcaaagaacagttcgtttctccgggccagca<br>ggaactggatgcagacaccgctctgccgctgggttctgaacagggtttcaaaatcccggaatgcctgaactgcg<br>gcggtctgatgaaaaccgatgttaccctgttcggcgaaaacctgaacaccgataaaatcaaagtttgcggtaaaa<br>aagtgaacgaatgcaacggcgttctgaccctgggtacctccctggaagtgctgagcggttaccagatcgttaac<br>cacgcgcacatgcagaacaaaccgatcttcatcgttaacatcggtccgacccgtgcggatcagatggcgacca<br>tgaaactggattaccgtatttctgatgttctgaaagaaatgtag
CELE_F46G10.3	185876	atgatgaaatacggcatggcacagaaattcgttccggaagcggctgagctgtgtgagaatagcctgaaaaaatt<br>catttctctgatcggcaccgttgataaactgctggttatcagcggcgcgggtatcagcaccgaatctgtgccggg<br>tatcccggattaccgtagcaaagatgttggtctgtatgcgcgtatcgcgcacaaaccgatctatttccaggattac<br>atgcgttccaaccgttgccgtcagcgttactggtctcgtaacttcctggcgtggccgcgtttcggccaggctgca<br>ccgaacattaaccactacgcgctgtctaaatgggaagcgtctgatcgtttccagtggctgatcacccagaacgtt<br>gatggtctgcacctgaaagctggttctaaaatggtgactgaactgcacggttctgcgctgcaggtgaaatgcacc<br>acctgtgattatattgaatctcgtcagacctaccaggatcgtctggattatgctaatccgggttttaaagaagaaca<br>cgttgcgccgggtgaactggcgccggatggtgatatcattctgccgctgggtactgaaaaaggcttccagatcc<br>cggaatgcccgtcttgtggtggtctgatgaaaaccgatgttaccttcttcggtgaaaacgttaacatggataaagtt<br>aacttctgctacgaaaaagttaacgaatgcgatggtatcctgagcctgggtaccagcctggcggttctgtctggtt<br>tccgtttcatccaccacgcgaacatgaaaaagaaaccgatcttcatcgttaacatcggcccgacccgtgcggatc<br>acatggcgaccatgaaactggattacaaaattagcgatgttctgaaagagatgtag
CELE_C06A5.11	182284	atgactagcgtgtatgaatcactgctgagtgattaccctgataaaggtgtgatcggtaaaccggaaattcgtgata<br>ccgaaaccgaaattattgaaaaactgcgtaccctgtataaccactttgttcaggcgaaacagaccggtaaaccga<br>tcttcgttctgattggcgcgggtgtgagcaccggctctaaactgccggatttccgtggtaaacagggtgtttggac<br>cctgcaggctgaaggtaaacacgctgaaggtgttgacttccaggttgcgcgtccgggtgttagccacaaatcca<br>tcctggcgctgcacaaagcgggctatattaaaaccatcatcactcagaacgttgatggcctggatcgtaaagttg<br>gtatcccggttgaagatctgatcgaagttcacggtaacctgttcctggaagtttgccagtcttgtttttctgaatatgtt<br>cgtgaagaaatcgttatgtctgttggcctgtgcccgaccggtcgtaactgcgaaggtaacaaacgtaccggtcgt

		agctgccgtggcaaactgcgtgatgcgaccctggattgggataccgaaatctctctgaaccacctggatcgtatccgtaaagcgtggaaacagaccagccacctgctgtgcatcggtacctctctggaaatcatcccgatgggtagcctgccactggatgcgaaaagcaaaggtatcaaaaccaccaccatcaactaccaggaaaccgcgcacgaaaaaatcgttgaaaccgcgatccacgcagatgttaaactgatcctgtactctctgtgtaacgcgctgggcgttaacgttgatctgggtgatgatctgccggatgaagttccgatcccgctgaaaatcagctaa
TK0685	3235398	atgctgggtcacgcggctaaactgctggcgcgtgctcgttttgctatcgctttcactggtgcgggcatttctgctgaaagcggtattccgaccttccgtggtcgtaacggtctgtggaaaacctaccgtgctgaagaactggcaaccccggaagcgttcaaacgtgatccgcacctggtttgggaattttataaatggcgtatgcgtaaaatcctgaaagcggagccgaacccggcgcacaaagcgctggctgaactggaaaacatgggtgttctgaaagcggtgatcacccagaacgttgatgatctgcaccgtgaagcgggtagccgtaaggttgtggaactgcacggtaacatcttccgtgttcgctgcgttagctgcagctaccgcgaaaacctgaaagaatctggccgtgttttcgagttcgttcgtgaaaaagaactgccgaaatgcccgaaatgcggttctctgctgcgtccggacgttgtttggttcggtgaaccgctgccgcgtgaagcgctggaagaagcattcagcctggcggaacgtgcggacgttgtgctggtcgttggtaccagcggtgttgtttacccggctgcttacgttccgtacatcgttaaagaacgtggtggcaaagttatcgaagttaacgttgaacgttctggtatcaccccgatcgcagatgttttcatccgtggcaaagcgggcgaagttatgccggaactgctgcgtcgtgtgaaagacatcatggccgaacgtaaccattacggctaa
4351669	4351669	atggcggcgggggcgcacgcgtcgcgcgcttcggctccgatcattgcaggtctcacaggtgctcttcgagcagcatataaaggattttctcctcagttatgcaacttccatgcgtcagtaaataatggacttctccacagaaggaaaatacaactccattttatatgctcttttcgctccatacaagcaagatataaccattcgtcagctgttgcacctaaggactattgcgaaacttacatccagtttctaagagataaacagatagttcctgattcagatcctccaagtgctaaggatgtggacctactttaccggtttattgatcagagtaaaaaactcatggtgttaactggagcaggaatgagcactgaatcaggaattcctgattataggagccctaatggtgcttacagttctggttttaaaccacttactcatcaggagtttgttcgttctattcgagcccgaaggcggtactgggctaggagctatgctggatggagaaggttcaggagagcacaaccaaactcagcccattatgctcttgcatcactggagagaattggccgggtacactcaatggttactcaaaacgtggataggttgcatcatcgtgctgggagtaagccagttgaattgcatggaagtgtgtatgaggtagcatgtttagactgtggtacatccattgaccgagaatcattccaggagcaggtcaaggacctcaacccaaagtgggctctagctattgacagtttggaagtaggacaaccgggctcagacaaaagtttcggaatgcagcagcgacctgatggtgatattgagattgatgagaagttctgggagcaagattttgatattcctagttgcaaccaatgtggtggagtgctgaaacctgatgttgtgatgtttggtgataatgttcctgaagagagagctgaaagcactaaggaggctgcaagaaattgtgatgctcttctagtggttggttcagcactaatgacaatgtctgcttttaggctcgcaaggcttgcacatgaagcgaatgctccaattgcggcgatcaccattggtgagaccagggctgatagtattttatccttgaaaattaatgcaagatgtggggagatactgcccagaatacttcagatgggaagtctagctgtaccaaacgtaagttaa
4335342	4335342	atgtcacttggctatgccgagaagctatcctaccgagaggatgtgggaaatgtcggcatgcctgagatcttcgactctcctgaactcctccacaagaagatagaagagcttgcagtcatggtgcgagagagtaagcatttggtggtgtttactggagcaggcatatcaacttcatcaggcatacctgacttccgagggcccaaaggcgtgtggacactgcagcgttcaggaaaaggtgttcctggtgcatcactgccgtttcaccgagctgttccaaccttgactcatatggcactggttgaattggaaaaaacaggacgattaaagtttgtcataagccagaatgttgatagcttacatctccgctctggcctcccaagggagaagctggctgaattgcatggaaattctttcaaggagatctgcccaagctgtaaaaaggagtaccttcgtgattttgaaattgagacgataggactgaaggatactccaaggcgttgttctgataagaactgtggagctagattaaaagacacagttcttgattgggaggatgctttaccccctgaggagatggatgctgccaaggagcagtgtcaaaaagctgatcttgtcctatgtttaggaactagtctgcagattactcctgcttgtaatatgcctctgttgtcactgaagaatggggggagggtggctattgtaaatcttcaggcaactccgaaggataaaaaggcaagccttgtcattcatggacttgtggataaggtcattgctggggttatgtatatgatgaatctgcgtatccctccatatattcgtactgactttgttcaaattagtctccggaactcggtcaaaaaaaaatgcgtgagatggactctccgagtaacaagcattcatggcttgcgag

		cacctttgccatttcttcgatcagttgaggtttcctttcctgaaagacctgacatgaagcctgtagtactaaaggagc<br>aaccattttctttgcagagagaaacatcaatgaatagaccgtttgtcatgctattgacattcaactttagtgatggttg<br>cggctgttcgagctcttccattgaatggcctgttgattttctgaagcaaaaagacagctttgtcagggatagaagtc<br>tagtcctgcaagagttgcaacatgctgcagagcatcgatcacgtgcagggcagcatgcaattctggagaggga<br>gggtgtgccaagagctgaaacgtcaattcatgcacttgtgaccaacatcgtcaggtatgatactgaagatagcaa<br>ggcggcagttcccatggctacatggatgaacagcaacggcagcctttcgaaacggcatatggatgccattggtt<br>gcaacccagcatcgtcaaaaaagcagaagttggttgctactcgtcatcggcgaaagggcttgaaccctgctact<br>cagaaagtttga
Rv1151c	886026	atgcgagtggcggtgctcagcggcgcggggatctccgcggaaagcggtgtaccgacgttccgcgatgacaa<br>gaacggattgtgggcccgattcgacccttacgagctgtccagcacgcaaggctggctgcgcaaccccgagcg<br>ggtctggggatggtacctatggcgccattatctggtggccaacgtcgaacccaacgacgggcatcgcgcgatc<br>gccgcctggcaggaccatgctgaggtcagcgtcatcacccagaatgtcgacgatctgcacgagcgcgccggc<br>agcggtgcggtgcatcacctgcacggcagccttttcgaatttcgttgtgcacgttgtggtgtgccctacaccgac<br>gcccttccggagatgcccgagcctgcgatcgaagtggagccgccggtctgcgactgcggcggtctgatccgg<br>cccgacatcgtatggttcggtgagccgctgccagaggagccgtggcggagcgcggtcgaggcgacaggga<br>gcgccgacgtgatggtcgtggtggggacctcggcgatcgtctacccggcggccggtttacccgacctggcgc<br>tggcgcgcggcactgccgtgatcgaagtcaatcccgagcccacgccgttgtccggcagcgcgacgatcagca<br>tccgcgagtcggccagccaagcgttgccggggctgttggagcgcctgcccgccctgctgaaatag
YOL068C	854086	atgaacatattgctaatgcaacggatagttagttttattttggtggttagtcaagggcgatactttcatgttggagagc<br>tgaccatgacaatgttgaaaagacctcaagaagaagagtcagataataatgctacgaagaagctgaaaacgag<br>attaacgtacccttgtatcctcgggaaggataaggtgacgggcaaattcatctttcctgcaatcactaaagatgat<br>gttatgaatgcaagactgtttttgaaggacaacgatttgaaaactttttttagaatattttttgccagtggaagtcaactc<br>gatctacatttatttcatgataaaattgctgggattcgacgtcaaagataaagaactatttatggcgttaaactctaat<br>atcacttctaataaagaacgttctagtgcggaactatcgtctatacatgcaaaagctgaagatgaggacgagttga<br>ccgatcccttagagaaaaagcatgcagtaaaattaatcaaagatttacaaaaagccataaataaagtccttagtac<br>aaggcttcgattacccaacttcaatacgattgatcatttcaccgccactttgcgtaacgccaaaaaaattttggttct<br>gacgggcgctggagtttccacttctttaggtattcccgattttagatcatctgaggggttttattctaagatccgaca<br>ccttggattggaagaccctcaggatgttttcaacttggatatattcttacaggacccttctgtttttttacaatattgccc<br>atatggttttaccaccagagaacatgtattctccgttgcacagcttcattaaaatgctacaagacaagggcaaacta<br>ttaagaaactacacacagaatatagataacttagaatcgtatgcagggatagatcctgataaattagtgcaatgtc<br>acggttcattcgccactgcatcttgtgtgacttgccattggcaaatacctggtgagaaaatattcgaaaatattaga<br>aaccttgaactaccattatgtccctactgttatcaaaaaagaaaacaatattttcctatgagtaatggcaataatacg<br>gtgcaaacaaacataaatttcaactcacccattttaaagtcgtatggtgttttgaaaccagacatgacatttttttggc<br>gaggccctgccatcgcgatttcataagaccatccgcaaagatatactggaatgcgatctattgatttgtataggaa<br>caagtttgaaagtggctcctgtgtctgaaattgtcaatatggtaccttcacatgttccgcagattttgatcaataggg<br>atatggttacacatgcagagtttgacttaaacctgttgggattctgtgatgacgtggccagtctcgtagctaaaaaa<br>tgccattgggatataccgcataaaaaatggcaagatctaaagaagatagattacaactgtacggaaatagataaa<br>ggtacgtataagattaagaaacagccacgaaagaaacaacagtaa
YOR025W	854190	atgacttcagtatcgccctcgccacctgccagtcgatcgggctcaatgtgctccgacttaccgtcctctttgcaga<br>ctgagaaactggcacatattataggtcttgatgccgacgatgaagttctccggcgcgtaaccaagcagttgagca<br>gatctaggagaattgcttgtctgactggggcaggcatttcgtgcaacgcgggcattcctgactttcgctcttctgat<br>gggctctacgacctagtgaaaaaggattgttcacagtattggtctatcaagtccggcagggaaatgtttgatatttc<br>gctatttagagatgacttcaaaatatccatttttgctaaatttatggagaggctctattcaaatgttcaattggcaaag<br>ccgactaagacgcacaagttcattgcgcatctaaaagataggaacaaactgctgcgctgttacacgcaaaacat

		cgatgggctcgaagaaagcataggacttactttatcaaataggaaattaccgcttacctcatttagttcacattgga<br>aaaatctggatgtcgttcagttgcacggcgacctgaaaactctttcgtgtacaaagtgcttccagacttttccctgg<br>agcaggtactggtctcgttgtctaagaagaggtgagttaccattgtgtccggattgcgaagcacttatcaacaaga<br>gattgaatgaaggaaagcgaactcttggttctaatgtgggtattctaagacctaatatcgtcctgtatggtgaaaac<br>catccatcctgtgaaattattacgcaaggcctaaatcttgacataattaaaggcaatcctgatttttgatcatcatgg<br>gtacaagtttgaaagttgatggtgtgaaacaactggtaaaaaaattaagtaagaaaattcacgatcgtggcggcc<br>tgatcattctcgtaaacaagacacccattggcgaatcctcttggcacggcattatagactaccaaatccactcaga<br>ttgtgataattgggtcacatttcttgaatcccaaataccagatttcttcaagacgcaagatcaaattaagaagttaag<br>acagttaaaaagggaggcgagcgacttgagaaagcaaatgaaggcccaaaaagactcaatcggaaccccccc<br>caacaacccctctacgaactgcccaggggattgatattcaaggaaacaacgaattgaatacaaaaataaagtcg<br>ttaaacacagttaagagaaaaatactgtcaccagaaaactccagtgaggaagacgaagaggaaaacttggata<br>caagaaaacgcgctaagatacgaccaactttcggtgacaaccaagcctcataa
YDR191<br>W	851772	atgaagcaaaaatttgtactaccgatcaccccaccaagtacggcggaaaagaagcctcaaacagaaaaccgtt<br>gcaatgagaatttgaagcctagaagattgctaccgcaattgaaaaaaagtgtccgtaacaggaaacctagactgt<br>catataggcctgagttaaactctgtgtttgatctggatgcgtacgtggattcgacacacttgtccaaatcccaacgc<br>catcatatggaccgcgacgccggttttattagctatgctctgaattatagcaaaagaatggttgtagtcagcgggg<br>caggtatatctgttgccgctggtataccggattttagatccagtgaagggattttctctactgtgaacggcggctct<br>gggaaagatttgtttgactacaatcgtgtgtatggcgacgaatcaatgagtttgaaatttaatcagttaatggtgtca<br>ttgttcagattatccaagaattgccaacctacaaaatttcatgaaatgctcaatgagtttgctagggatggcaggct<br>attgagactgtacacgcagaatattgacggtttagatacacaattacctcatttatcaactaatgtgcctctggcgaa<br>gccaatacccagtacagtacaattacacggaagcataaaacacatggaatgcaataaatgtctgaatatcaaacc<br>ttttgaccctgaactgttcaaatgcgacgacaaatttgattctcggactgaaatcataccgtcatgtccacaatgtga<br>agaatatgaaacagttagaaaaatggcaggcttaagatctactggtgtgggcaagttgcgtccaagagtaattta<br>tacaatgaggtccaccctgaaggtgattttatcggtgaaattgccaataatgacttaaagaaaagaattgattgtttg<br>attattgttggaacgagtttgaagattcccggagtgaaaaatatttgcaggcagtttgcggccaaagtccatgcaa<br>acaggggtattgtgttatatttaaacaccagtatgccgcctaagaatgtgctagactctttgaaatttgtagacctag<br>ttgtactgggagattgtcagcatgttacctcattattaggcggcggcggcagccaccaccaccaccaccactaa
Saci_0381	3473325	atgctgccgtactctgttaacagcagcttcctgaactactctggtctgatgtacctggttgaagaagcgaaaaaag<br>ttgcggaaatgatcctgagcagcgttaacgcgatcgcgttcaccggtgcgggtatcagcaccgcgagcggtat<br>cccggatttccgtggtccgcagggtctgtggaaaaaatactctccggaactggcgagcattgaatacttccagaa<br>atacccggatgcgttctggcagttctacagcacccgtatgaaatctctgttcgaagcgaaaccgaaccgcgcgc<br>actacgcgctggcgcagctggaaaaaatgggtctgatcaaagcggttatcacccagaacgttgatggtctgcac<br>agcgtggcaggtagccgtaacgtgatcgaactgcacggcaacatgcgtaaatcttactgcaccagctgcctgc<br>gtagctacgatagcctggaagttctggcgcgtgttgaaaaaggtgaagttatcccgcgttgcgaatgcggtggta<br>tcctgaaaccggatgttgttctgttcggtgaaccggttcacggtatctacgaagcgatgcgtatcgcgaacgaaa<br>gcgatctggttctggcgatcggttcttccctgaccgtttacccggcgaaccagatcccgctgatcgttaaacgtaa<br>cggcggcggtctgatcatcctgaacggcgaagaaaccccgtacgatgaatacgcggatctggttatccgtgaa<br>cgtatcgaaatcttcctgccggaagttatcagccacatccagtctctgcaccgttctagctaa
SIRT1	23411	atggctgatgaagcggccctggcgctgcagccgggtggttctccgtccgcggcgggcgctgatcgtgaagcg<br>gcctcttctccggcgggtgaaccgctgcgtaaacgtccgcgtcgtgatggtccgggcctggaacgctctccgg<br>gtgaaccgggcggcgcggcgccggaacgtgaagttccggctgcagctcgtggttgtccgggcgcggcggc<br>agcggctctgtggcgtgaagctgaagctgaagcagcagcggcaggtggtgaacaggaagcgcaggcaacc<br>gcagctgcgggtgaaggtgacaacggtccgggcctgcagggcccgagccgtgaaccgccgctggcggata<br>acctgtatgacgaagatgacgatgatgaaggtgaagaagaagaagcagcggcggccgcgatcggctat

		cgtgacaacctgctgttcggcgatgaaatcatcaccaacggttttcatagctgtgaatctgatgaagaggatcgtg<br>cttcccacgcgtctagctccgattggaccccgcgtccgcgcatcggcccgtataccttcgtgcagcagcacctg<br>atgatcggtaccgacccgcgtaccattctgaaagatctgctgccggaaactatcccgccgcctgaactggatga<br>catgactctgtggcagatcgtgattaacatcctgagcgaaccgcctaaacgtaagaaacgcaaagacatcaaca<br>ccattgaagatgcggtaaaactgctgcaggaatgcaagaagatcatcgttctgactggcgcaggcgtaagcgtt<br>agctgtggtattccggatttccgctctcgtgacggtatttacgcacgcctggctgttgatttcccggacctgccgga<br>cccgcaggctatgttcgatatcgaatattttcgcaaagatccgcgcccgttctttaaattcgccaaagaaatttatcc<br>tggtcagttccagccgagcctgtgccataaattcatcgcactgagcgacaaagaaggtaaactgctgcgtaact<br>acacccagaacatcgataccctggaacaggttgccggtatccagcgcattatccagtgtcacggctccttcgca<br>accgcatcctgtctgatctgcaagtacaaagtggattgtgaagcagttcgtggtgatattttcaaccaggtggttcc<br>gcgttgcccgcgttgcccggcggacgaaccgctggccattatgaaaccggaaattgtgttcttcggtgaaaacc<br>tgccggaacaattccaccgcgcaatgaaatatgataaagatgaagttgacctgctgatcgtgattggtagctctct<br>gaaagttcgtccggtggcactgatcccgtctagcatcccgcacgaagttccgcaaatcctgatcaaccgtgaac<br>cgctgccgcacctgcactttgacgttgaactgctgggcgactgcgatgttatcattaacgaactgtgccaccgtct<br>gggtggcgaatacgctaaactgtgctgtaacccggttaaactgtctgagatcaccgaaaaaccgcctcgtaccc<br>agaaagaactggcgtatctgtcggaattgccgccgaccccgctgcacgtttccgaagattcttctagcccggaa<br>cgtaccagcccgccggattcctccgttatcgttaccctgttagatcaggcggcaaaaagcaacgatgacctgga<br>cgtttcagaaagcaagggctgtatggaagaaaaaccgcaagaagttcagaccagccgtaacgtagaaagcatt<br>gctgaacagatggaaaacccggacctgaaaaacgtgggctcttctaccggtgaaaagaacgaacgtaccagc<br>gtggccggtaccgtacgtaaatgctggccgaaccgtgttgcaaaagaacagatcagccgtcgtctggatggta<br>accagtacctgttcctgccgccgaaccgttacatctttcacggcgctgaagtgtactcggactccgaagatgatgt<br>tctgagctctagcagctgtggctctaactccgacagcggcacctgccagtctccatctctggaagaaccgatgg<br>aagatgaatccgaaattgaagaattttataacggcctggaagatgaaccggacgtgccggaacgcgccggtgg<br>tgcaggcttcggcactgatggtgatgatcaggaagctattaatgaagcgatctcagttaaacaggaagttaccga<br>tatgaattacccgtctaataaatcaggcggcggcggcagccaccaccaccaccaccactaa
SIRT2	22933	atggcggaaccggatccatctcacccgctggaaacccaggcgggtaaagttcaggaagcgcaggattctgatt<br>ctgattccgaaggcggtgctgcgggcggcgaagctgatatggatttcctgcgtaacctgttcagccagaccctg<br>tctctgggcagccagaaagaacgcctgctggatgaactgaccctggaaggcgttgcgcgctacatgcagagc<br>gaacgttgccgtcgtgtgatttgcctggttggcgctggtatctccacctctgcgggtatcccggacttccgtagcc<br>cgagcaccggcctgtacgataacctggaaaaataccatctgccgtacccggaagcgatcttcgaaatcagctac<br>ttcaaaaaacacccggaaccgttcttcgcgctggcgaaagaactgtatccgggtcagtttaaaccgaccatctgc<br>cactatttcatgcgtctgctgaaagacaaaggcctgctgctgcgttgctacacccagaacattgataccctggaa<br>cgtatcgcgggcctggaacaagaggacctggttgaagcgcacggtaccttctacaccagccactgcgtttctgc<br>gagctgccgtcacgaatacccgctgagctggatgaaagaaaaaatcttcagcgaagtgaccccgaaatgcga<br>agattgccagtctctggttaaaccggacattgttttcttcggtgaaagcctgccggcgcgtttcttcagctgcatgc<br>agtccgatttcctgaaagttgacctgctgctggttatgggcacctctctgcaggttcagccgttcgcgagcctgat<br>cagcaaagcgccgctgagcaccccgcgtctgctgatcaacaaagaaaaagcgggtcagagcgatccgttcct<br>gggtatgatcatgggcctgggcggtggtatggatttcgatagcaaaaaagcgtaccgtgatgttgcgtggctgg<br>gtgaatgcgatcagggctgcctggcgctggcggaactgctgggctggaaaaaagaactggaagatctggttc<br>gccgtgaacacgctagcatcgatgctcagagcggcgcgggtgttccgaacccgtctaccagcgcgagcccga<br>aaaaatccccgccgccggctaaagatgaagcgcgtaccaccgaacgtgaaaaaccgcagggcggcggcgg<br>cagccaccaccaccaccaccactaa
SIRT3	23410	atggcgttttggggctggcgtgcggcggcagcgctgcgtctgtggggccgtgttgttgaacgtgttgaagcagg<br>tggtggcgtaggcccgttccaggcgtgtggttgccgcctggttctgggcggccgtgatgacgttagcgcgggc

		ctgcgtggcagccacggtgcgcgcggtgaaccgctggatccggcgcgtccgctgcagcgtccgccgcgccc<br>ggaagttccgcgtgcattccgtcgccaaccgcgtgcggcggcaccgagctttttcttctccagcattaaaggcg<br>gccgccgtagcattagcttctctgttggcgcgagctctgtggttggcagcggcggcagctccgacaaaggcaa<br>actgagcctccaggatgtggctgaactgattcgtgcccgcgcttgccagcgcgttgttgttatggttggtgcgggt<br>atctccaccccgtccggtattccggacttccgtagcccaggctctggcctgtatagcaacctgcagcagtacgat<br>ctgccgtatccggaagcgatcttcgaactgccgtttttctttcacaacccgaaaccgttcttcaccctggcgaaag<br>aactgtatccgggcaactacaaaccgaacgtgacccactacttcctgcgtttgctgcacgataaaggcctgctgc<br>tgcgcctgtacacccagaacattgatggtctggaacgcgttagcggtatcccggccagcaaattggttgaagct<br>cacggtaccttcgcgtccgcaacttgcaccgtttgccagcgtccgtttccgggcgaagatatccgcgctgacgt<br>gatggcggatcgtgtgccgcgttgcccggtttgtaccggtgttgttaaaccggatatcgttttctttggtgagccgc<br>tgccgcagcgtttcctgctgcacgttgttgacttcccaatggctgacctgctgctgatcctgggcaccagcctgga<br>agttgaaccgttcgcgtccctgaccgaggcggttcgctcctctgtgccgcgcctgctgattaaccgtgatctggt<br>gggcccgctggcgtggcacccgcgtagccgtgatgtggcgcagctgggtgatgttgttcacggtgttgaaagc<br>ctggttgaactgctgggctggaccgaagaaatgcgtgacctggttcagcgtgaaaccggcaaattagatggtcc<br>tgataaaggcggcggcggcagccaccaccaccaccaccactaa
SIRT4	23409	atgaagatgagctttgcgttgactttcaggtcagcaaaaggccgttggatcgcaaaccccagccagccgtgctc<br>gaaagcctccattgggttatttgtgccagcaagtcctcctctggaccctgagaaggtcaaagagttacagcgctt<br>catcaccctttccaagagactccttgtgatgactggggcaggaatctccaccgaatcggggataccagactaca<br>ggtcagaaaaagtggggctttatgcccgcactgaccgcaggcccatccagcatggtgattttgtccggagtgcc<br>ccaatccgccagcggtactgggcgagaaacttcgtaggctggcctcaattctcctcccaccagcctaaccctgc<br>acactgggctttgagcacctgggagaaactcggaaagctgtactggttggtgacccaaaatgtggatgctttgca<br>caccaaggcggggagtcggcgcctgacagagctccacggatgcatggacagggtcctgtgcttggattgtgg<br>ggaacagactccccggggggtgctgcaagagcgtttccaagtcctgaaccccacctggagtgctgaggccca<br>tggcctggctcctgatggtgacgtctttctctcagaggagcaagtccggagctttcaggtcccaacctgcgttcaa<br>tgtggaggccatctgaaaccagatgtcgttttcttcggggacacagtgaaccctgacaaggttgattttgtgcaca<br>agcgtgtaaaagaagccgactccctcttggtggtgggatcatccttgcaggtatactctggttacaggtttatcctc<br>actgcctgggagaagaagctcccgattgcaatactgaacattgggcccacacggtcggatgacttggcgtgtct<br>gaaactgaattctcgttgtggagagttgctgcctttgatagacccatgctga
SIRT5	23408	atgcgtccgctgcagatcgttccgagccgtctgatctcacagctgtattgtggtttaaaaccaccggcttctaccc<br>gtaaccaaatttgcctgaaaatggcacgtccgtcctcctctatggcagatttccgtaaatttttcgctaaagcaaaa<br>cacatcgttattatttctggtgctggtgtgagcgcagagtctggtgttccgaccttccgtggcgcgggcggctact<br>ggcgtaaatggcaggcgcaggatctggcgaccccgctggcgttcgcgcacaacccgtcccgtgtttgggaatt<br>ctaccactaccgtcgtgaagttatgggctctaaagaaccgaacgctggccaccgtgcgatcgcggaatgcgaa<br>acccgtctgggtaaacagggtcgtcgcgttgttgttatcacccagaacattgatgaactgcaccgtaaagcgggt<br>actaaaaacctgctggaaatccacggcagcctgttcaaaacccgttgcaccagctgcggtgttgtggcggaaaa<br>ctacaaaagcccgatctgcccggctctgagcggtaaaggcgcgccggagccgggcacccaggatgcgtcca<br>tcccggttgaaaaactgccgcgttgcgaagaagcgggttgcggtggcctgctgcgtccgcacgtggtgtggttc<br>ggtgaaaacctggatccggcgatcctggaagaagtggatcgtgagctggcgcactgcgatctgtgcctggtgg<br>ttggcacctctagcgttgtttacccggcggcgatgttcgcgccgcaggtagcagcgcgtggcgttccggttgct<br>gaattcaacactgaaaccactcctgcaactaatcgttttcgttttcacttccagggtccgtgtggtaccaccctgcc<br>ggaagcactggcttgccatgaaaacgaaaccgttagcggcggcggcggcagccaccaccaccaccaccact<br>aa
SIRT6	51548	atgagcgttaactacgcggcgggtctgagcccgtacgctgataaaggtaaatgcggtctgccggaaatcttcga<br>tccgccggaagaactggaacgcaaagtttgggaactggcgcgtctggtttggcagtctagctctgttgtgttcca

		caccggcgccggcatcagcaccgcgtctggtatcccggatttccgtggcccgcacggtgtgtggacgatgga<br>agaacgcggcctggcgccgaaattcgataccaccttcgaaagcgcgcgcccgacccagacccacatggctct<br>ggtgcagctggaacgcgtgggcctgctgcgtttcctggttagccagaacgtggatggcctgcacgtgcgttctg<br>gtttcccgcgtgataaactggcggaactgcacggtaacatgttcgtggaagaatgcgccaaatgtaaaacccag<br>tatgtgcgtgataccgttgtgggcacgatgggtctgaaagcgaccggtcgtctgtgcaccgttgcgaaagcacg<br>cggcctgcgtgcatgccgcggcgaactgcgtgataccattctggattgggaagattctctgccggaccgtgatc<br>tggccctggctgatgaagcatctcgtaacgctgatctgtctattaccctgggtacctccctgcagatccgtccgtct<br>ggcaacctgccgctggcgaccaagcgtcgtggcggccgtctggttatcgttaacctgcagccgaccaaacac<br>gatcgtcacgctgacctgcgtatccacggctatgttgacgaagtgatgacccgtctgatgaaacacctgggcct<br>ggaaatcccggcgtgggatggcccgcgcgttctggaacgcgcgctgccgccgctgccgcgtccgccgaccc<br>cgaaactggaaccgaaagaagaatcgccgacccgtatcaacggtagcatcccggcgggtccgaaacaggaa<br>ccgtgcgcgcagcacaacggctctgaaccggctagcccgaaacgtgaacgtccgacctctccggctccgcac<br>cgcccgccgaaacgtgttaaagcgaaagcggttccgtctggcggcggcggcagccaccaccaccaccacca<br>ctaa
SIRT7	51547	atggcggcgggtggtctgagccgtagcgaacgtaaagctgctgaacgtgttcgtcgtctgcgtgaagaacagc<br>agcgtgaacgtctgcgtcaggttagccgtatcctgcgtaaagcggcggctgaacgttctgcggaagaaggtcg<br>tctgctggcagaaagcgcggatctggtgaccgaactgcagggtcgctcccgtcgtcgtgaaggtctgaaacgt<br>cgtcaggaagaagtttgtgatgatccggaagaactgcgtggtaaagttcgtgaactggcgagcgcggtgcgta<br>acgcgaaatacctggttgtgtataccggtgctggtatcagcactgcggcgagcatcccggattaccgcggccc<br>gaacggtgtttggaccctgctgcagaaaggtcgttctgtttccgcggcggacctgtctgaagcggaaccgaccc<br>tgacccacatgtctatcacccgtctgcacgaacagaaactggttcagcatgttgtgagccagaactgcgacggc<br>ctgcacctgcgtagtggcctgccgcgcaccgcgatttctgaactgcatggtaacatgtacatcgaagtttgcact<br>agctgcgttccgaaccgtgaatacgttcgcgttttcgacgtaaccgaacgtactgcgctgcaccgtcaccagact<br>ggtcgtacctgccataaatgtggcacccagctgcgtgataccatcgttcacttcggtgaacgtggtaccctgggt<br>cagccgctgaactgggaagcggcaactgaagctgcaagccgtgcggacaccatcctgtgcctgggttcctctc<br>tgaaagtgctgaaaaaatatccgcgtttgtggtgcatgaccaaaccgccgagccgtcgtccgaaactgtacatc<br>gttaacctgcagtggaccccgaaagatgattgggcggcactgaaactgcacggtaaatgcgatgatgttatgcg<br>tctgctgatggcggaactgggtctggaaatcccggcgtactctcgctggcaggatccgatcttcagcctggcga<br>ccccgctgcgtgcgggcgaagaaggttcccactctcgtaaatctctgtgccgttctcgtgaagaagcaccgccg<br>ggcgatcgcggtgcgccgctgtctagcgcgccgattctgggtggctggttcggccgtggttgcaccaaacgta<br>ccaaacgtaaaaaagttaccggcggcggcggcagccaccaccaccaccaccactaa

*, Gene ID in NCBI database.

Supplementary Table 6. The 101 sirtuin amino acid sequences used in this study.

Sirtuin	Amino acid sequence
Thermococcus TK0685*	MLGHAAKLLARARFAIAFTGAGISAESGIPTFRGRNGLWKTYRAEELATPEAFKRDPHLVWEFYKWRMRKILKAEPNPAHKALAELENMGVLKAVITQNVDDLHREAGSRKVVELHGNIFRVRCVSCSYRENLKESGRVFEFVREKELPKCPKCGSLLRPDVVWFGEPLPREALEEAFSLAERADVVLVVGTSGVVYPAAYVPYIVKERGGKVIEVNVERSGITPIADVFIRGKAGEVMPELLRRVKDIMAERNHYG
Sulfolobus Saci_0381*	MLPYSVNSSFLNYSGLMYLVEEAKKVAEMILSSVNAIAFTGAGISTASGIPDFRGPQGLWKKYSPELASIEYFQKYPDAFWQFYSTRMKSLFEAKPNRAHYALAQLEKMGLIKAVITQNVDGLHSVAGSRNVIELHGNMRKSYCTSCLRSYDSLEVLARVEKGEVIPRCECGGILKPDVVLFGEPVHGIYEAMRIANESDLVLAIGSSLTVYPANQIPLIVKRNGGGLIILNGEETPYDEYADLVIRERIEIFLPEVISHIQSLHRSS
Nitrosopumilus Nmar_1777	MKMFESIKDQVKNAKKIVFVTGAGISQESGIPTFRGKDGLWRNYDAMKLATIDAFYDDPKLVWEWYNERRHNIFSANPNQGHKAIAELEKFADVVSLTQNIDGLHQKAGSTKVLELHGSIVKIKCTVCDFSDEIMTDFTENPPLCKCGSILRPDVVWFGESLPQDVWQEAIIHANQCDLMIIVGTSLVVSPANTLPIYAKQNNAMLIEINPENTEMSSEMDLVIRNTSANALPEFVSLFKNL
Thermococcus onnurineus TON_0819	MIEEASKLLARSRFAIAFTGAGISAESGVPTFRGFNGLWKKHRPEELATPEAFRKDPHLVWSFYKWRMGLIMKARPNRAHYALAELEEMGILKAVITQNVDDLHREAGTKNLIELHGNIFRVRCTSCGYEENLKENGRLEEFLVQKDLPKCPNCDSLLRPDVVWFGEPLPRKALDEAFKLAEKADLVLVIGTSGVVYPAAYIPQIVRETGGKVIEVNPEESGITPIADVFLRCPAGEAMEKLMKRIKGLI
Haloarcula marismortui rrnAC0470	MADDRHGIDDIDGETLDAVAEALRTAETAVALTGAGVSTASGIPSFRGDDGIWERHDPADFHRRRLDADPAGFWADRLSLREAIYGDIDPEPNAAHEALAALEADGHLDAVLTQNIDGLHDAAGTDRVVELHGTHRRVVCDDCGHRRDAEVVFEQAAESSDLPPRCDCGGVYRPDVVLFGEPMPDVAMNEAQRLARDSDVFLAVGSSLSVQPASLLPKIAAEGDSTLVVVNYEETPRDASAAHVLRADVTQVLPAIVERL
Natrarchaeobaculum aegyptiacum B1756_12320	MDDLESLAAEIDRASTVVALTGAGISAPSGVPTFRGDDGVWEHFDEGQFTYGRFQRDPAGFWDDRGDLQRELFGEAYEPNAAHVALAELAAEGYLDAVVTQNTDGLHRDATARVRGATADGENDTTDESEGTDHEILELHGNARRVRCVDCGRRQDADPVFERAEDGDLPPRCDCGGTFKPDVVLFGEQLPGAVIQRARTLAGESDVFLAIGSSLVVQPAASLPRQAAADGTLAVINLEETPCDGDAAVVCRADVTEAVPRLRELVR
Natrarchaeobaculum aegyptiacum B1756_03690	MTGDDVVRAADLVAESDHLVAFTGAGVSTESGIPDFRSPGGVWDRFDPEDFTIQALTRDPVSYWETRVEMRRERDFDWDEVEPNPAHEAIARLEREGPLSAVITQNVDGLHQEAGNADESVLQLHGTHERAKCLDCGDRFPLETLEAQLEAESLPPECDDCGGLLKYATVSFGERLPQDVLERARREAAACDCFVVVGSSVTVEPAASMPRIAARNGASLVVVNLDETPVDGLADAVVRGKAGETLPSIVERAIA

Pyrobaculum aerophilum PAE2959	MAVDFTTDELDEVASLIARSSCNVALTGAGVSTASGIPDFRGPQGVWRRVDPEKFEISYFYNNPDEVWDLFVKYLLPAFNVKPNPAHYALAEMERLGKLCAVITQNVDRLHQAAGSKNVIELHGALEYAVCTNCGSKYALAEALKWRKSGAPRCPKCGGVIKPDVVFFGEPLPQDALREAFMLAEMAEVFMAIGTSLAVYPANQLPLVAKKRGAKLVIINADETYYDFFADYIIRGRAEEVLPKLLDRLRGMLF
Pyrobaculum aerophilum PAE3500	MNVADLLASSRHCVVFTGAGISAESGVPTFRGPGGLWERYKPEELATPEAFARDPALVWRWYKWRQEVIYNARPSPGHYAIAELEAMGVVRGVITQNVDGLHQRAGSRLVVELHGSIWRARCVKCGSVYILDKPVEEVPPLCRKCGGLLRPDVVWFGEPLPQEAWRAAVELASVSDVLLVVGTSGVVYPAAYIPRIAKEAGARVVEINVEPSAITPIADVFIQGRAGEVLPRLVEEVKRRLRTRQALTP
Saccharolobus solfataricus P2 SSO2478	MIYEKVAEELISSSYTIAFTGAGISTASGIPDFRGPQGLWKKYSPELASIEYFEKDPKNFWGFYSLRMRGLFEAQPNKAHYSLAELEKMGIIKVIITQNIDGLHQKAGSKNVIELHGTMRRSYCVLCLRTYDSLNVLSMIEKGNLPPRCDCGGIIRPDVVLFGEPVKNIYEALSIAYESDLVISIGSSLTVYPANLIPQTVKERGGKLIILNMEETPLDSIADYVVREPVEISLPKILENVRQKILS
Candidatus Nitrosocosmicus oleophilus NMY3_03230	MEGIEGDYLEDIIQIQKIIDKSENFVFFTGAGISRESGIPTFRDKDGLWKKYDPAKLASHSAFISNPELVWDFFYSRQRLVCQAECNDAHTAIGRFENTRPENSHVITQNIDRLHQRGGSQNVIELHGNIFGMVCLACGKRKQYDYFDFFNNFSEEKIPTCPGCDNILKPDVVLFEEPLPQDAWIQAIRLSSECDIMFVIGTSLNVSPANTLPYHAVKNHAVLVEINPNVTEMTSLMDFSIRASASKILTKILCVA
Archaeoglobus fulgidus DSM 4304 AF_0112	MEDEIRKAAEILAKSKHAVVFTGAGISAESGIPTFRGEDGLWRKYDPEEVASISGFKRNPRAFWEFSMEMKDKLFAEPNPAHYAIAELERMGIVKAVITQNIDMLHQRAGSRRVLELHGSMDKLDCLDCHETYDWSEFVEDFNKGEIPRCRKCGSYYVKPRVVLFGEPLPQRTLFEAIEEAKHCDAFMVVGSSLVVYPAAELPYIAKKAGAKMIIVNAEPTMADPIFDVKIIGKAGEVLPKIVEEVKRLRSEK
Archaeoglobus fulgidus DSM 4304 AF_1676	MDEKLLKTIAESKYLVALTGAGVSAESGIPTFRGKDGLWNRYRPEELANPQAFAKDPEKVWKWYAWRMEKVFNAQPNKAHQAFAELERLGVLKCLITQNVDDLHERAGSRNVIHLHGSLRVVRCTSCNNSFEVESAPKIPPLPKCDKCGSLLRPGVVWFGEMLPPDVLDRAMREVERADVIIVAGTSAVVQPAASLPLIVKQRGGAIIEINPDETPLTPIADYSLRGKAGEVMDELVRHVRKALS
Pyrococcus furiosus DSM 3638 PF1154	MPFQPSKYELTVNLVQGNYMLHVLSLKGEYKNISPKEILRYPSTNSLMLGEVSKILAKSSMAIAFTGAGISAESGIPTFRGKDGLWRKYRAEELATPEAFKRDPKLVWEFYKWRIKKILEAKPNPAHIALAELEKMGIIKAVITQNVDDLHREAGSKNVIELHGNIFRVKCTSCSYREYLKESDRIGWLLSQELPRCPKCGSLLRPDVVWFGEALPEKELTTAFSLAKKADVVLVVGTSGVVYPAAYIPYIVKESGGIVVEINIEPSAITPIADFFLRGKAGEVLPKLVEEIRRISK
Thermodesulfobacterium	MNLYKEIADLIKNSKYSLAFTGAGISVESGIPTFRGSQGLWSKYDP

TOPB45_0494	EEFAHIDSFIRNPAKVWKMIREMFAIIFEAKPNPAHEILAEMEKRGY LKAIITQNIDGLHQLAGSKNVIEYHGNCKWLLCLSCGKKEEVKRE LIEMLPYPKCKECEAPLKPDVVFFGEAIPFEAKTKAEREVQRCDLL LIIGTSGVVYPASQLPYMAKLNKATIIEINLEETPYTHSITDYFLKGK AGEILFKIFSELS
Bacteroides BT_2975	MKNLVVLTGAGMSAESGISTFRDAGGLWDKYPVEQVATPEGYQR DPALVINFYNARRKQLLEVKPNRGHELLAELEKNFNVTVITQNVD NLHERAGSSHIVHLHGELTKVCSSRDPYNPHYIKELKPEEYEVKM GDKAGDGTQLRPFIVWFGEAVPEIETAVRYVEKADIFVIIGTSLNVY PAAGLLHYVPRGAEVYLIDPKPVDTHTSRSIHVLRKGASEGVEEL KQLLIPAP
Escherichia b1120*	MLSRRGHRLSRFRKNKRRLRERLRQRIFFRDKVVPEAMEKPRVLV LTGAGISAESGIRTFRAADGLWEEHRVEDVATPEGFDRDPELVQAF YNARRRQLQQPEIQPNAAHLALAKLQDALGDRFLLVTQNIDNLHE RAGNTNVIHMHGELLKVRCSQSGQVLDWTGDVTPEDKCHCCQFP APLRPHVVWFGEMPLGMDEIYMALSMADIFIAIGTSGHVYPAAGF VHEAKLHGAHTVELNLEPSQVGNEFAEKYYGPASQVVPEFVEKLL KGLKAGSIA
Salmonella STM1221*	MQSRRFHRLSRFRKNKRLLRERLRQRIFFRDRVVPEMMENPRVLV LTGAGISAESGIRTFRAADGLWEEHRVEDVATPEGFARNPGLVQTF YNARRQQLQQPEIQPNAAHLALAKLEEALGDRFLLVTQNIDNLHE RAGNRNIIHMHGELLKVRCSQSGQILEWNGDVMPEDKCHCCQFP APLRPHVVWFGEMPLGMDEIYMALSMADIFIAIGTSGHVYPAAGF VHEAKLHGAHTVELNLEPSQVGSEFEEKHYGPASQVVPEFVDKFL KGL
Klebsiella YP_006636908.1	MKPEAMAKPRVVVLTGAGISAESGIKTFRAADGLWEEHRVEDVAT PEGFARDPALVQAFYNARRRQLQSPEIKPNAAHLALARLEDLLGD HFLLVTQNIDNLHERAGNRRVIHMHGELLKVRCSWSGQVLEWTG DVTAEDKCHCCQFPAALRPHVVWFGEMPLGMDEIYSALADADIFI AIGTSGHVYPAAGFVHEARLHGAHTVELNLEPSQVGSEFAEKHYG LASEVVPAFIDKLLQENAL
Vibrio AIL70671.1	MNFPYRNIVVLTGAGISAESGIQTFRAQDGLWENHRIEDVATPEGF ARDPDLVQDFYNQRRKKLQDPNIEPNAAHLALGRLEAELDGQVTI VTQNIDNLHERGGNKNIIHMHGELLKSRCSVSNQVIEETGDILTGD LCHCCQMPSQMRPHVVWFGEMPLRMGEIYSALETADLFISIGTSG VVYPAAGFVHDAKMHGAHTIEINLEPSAIESEFVEKRYGKASVEV PKLVEELLAHLESNVENA
Pseudomonas PA1125*	MRAVVELLAGARRLVIFTGAGVSAESGIPTFRDALGGLWARYDPA ALATPAAFADDPALVWGWYEWRRLKVLGVQPNPAHRAIAALSGR IANTRLVTQNVDDLHERAGSRDVLHLHGSLHAPRCATCAAAYRD ALPDSVEPEEGRRIEPPRCPACGGQVRPGVVWFGEALPEAALREA FAAACECDLLLSVGTSGVVQPAARIPGLALEHGASVVHVNPQPVR TRHPREHCLVGPAGEVLPELLRRAFPG
Pseudomonas PA1197*	MDSHSPIATVAQALRRAERILVITGAGLSADSGMPTYRGLGGLYNG

	RTEEGLPIEAALSGPMLRRDPALCWKYLAELGKACLAARPNAGHE AIAELQKHKPECWVLTQNIDGFHRQAGSPAERLIEIHGELAPLYCQ SCGAESGGLEEHLHGQLPPRCAACGGVLRPPVVLFEEMLPEEAID TLYRELRKGFDAVLVVGTTASFPYIVEPVLRTRQAGGFTAEVNPGV TDLSERVDVKMTGRALDIMPQVVSHIYR
Caulobacter CCNA_03594*	MRGIMKVFVLTGAGVSAESGLGTFRDKDGVWTKYDLNEVATPQG FARNPALVRDFYNARRANLAGARPNAAHDALAQLEAGLARRGGE LFLCTQNVDDLHEKAGCRRVIHMHGELAVTRCHHCEATWPDTGP LKPDTVCAACARDGGARPHVVWFGEIPLFMDQIEDALSAADLFVS IGTSGSVYPAAGFVAEARAMGIATCEINLEPSANAYVFDEKVYGPA TEVVPAWVERLLARL
Rhodobacter YP_003579259.1	MARIVILTGAGVSAESGIRTFRASDGLWEEHRIEDVATPEGFARDPA LVHRFYNQRRAAAAAALPNAAHLALARLEAAGAHQLLVVTQNV DDLHERAGTQNLIHMHGTLAGALCAACGHRWPAPAEMAPQDSCP RCLHPATRPDIVWFGEMPYRMEEIWAALRAADLFVSIGTSGNVYP AAGFVADARHGGVATLELNLEPSQGTRFFDEARHGPATQVVPAWV DEMLCT
Bacillus BSU09650	METFKSILHEAQRIVVLTGAGMSTESGIPDFRSAGGIWTEDASRME AMSLDYFLSYPRLFWPKFKELFQMKMSGSFEPNEGHLLLAELEKQ GKQVDIFTQNIDGLHKKAGSRHVYELHGSIQTAACPACGARYDLP HLLEREVPECTAAGNNGDICGTVLKTDVVLFGDAVMHFDTLYEKL DQADLLLVIGTSLEVAPARFVPEDASLIPGMKKVIINLEPTYCDSLF DMVIHQKIGEFARSLGMKK
Staphylococcus YP_007580196.1	MFILRNDLETLKHIIDSSNRITFFTGAGVSVASGVPDFRSMGGLFDE ISKDGLSPEYLLSRDYLEDDPEGFINFCHKRLLFVDTKPNIVHDWI AKLERNQQSLGVITQNIDGLHSDAGSQHVDELHGTLNRFYCNAC HKSYTKSDVIDRTLKHCDNCGGAIRPDIVLYGEMLDQPTIIRALNKI EHADTLVVLGSSLVVQPAAGLISHFKGDNLIIINKDRTPYDSDATLV IHDDMVSVVKSLMTE
Clostridium CA_C0284	MLLLDKINELKKIVAESSSIVFFGGAGVSTESNIPDFRSENGLYKTK NNFSYPPEVMLSHTFFKNHTEDFFEFYREKMIFKDAKPNAAHYSL AKIEEQGKLKAIVTQNIDGLHQLAGSKNVYELHGSIHRNYCMDCG KSFDLEYVIKSETTIPKCDKCGGIVKPDVVLYEEGLDDSIIQNSVKA ISEADTLIVGGTSLVVYPAAGLIRYFKGNKLILINKSATAYDNEADL VISDSIGKVLETVI
Clostridioides CDIF630_01458*	MDANSLKDLIANHNNIVFFGGAGVSTESNIPDFRSSTGLFSQKLNK QFTAEQLVSHTFFVRYPEEFFEFYKDKLIYPNAKPNNAHIALAKLE EMGKLKAVITQNIDGLHQMAGSKNVLELHGSVHRNYCTKCGKFF DLESMLNLGGNIPYCDNCGSIVKPDVVLYEEALDSDVITKTISAISN ADLLIIGGTSLAVYPAASFIDYYKGDYIALINKANTVYDKSASLVIN KPIGEVLYEAVLRQI
Mycoplasma MFE_08300	MVQNFNEKIDKFKNWIKESKHIVFFSGAGVSTASGIPDFRSADGLY SKKFKNMNPESILSRSFWRKNKKDFYEYYFSNIAFDNIKPNIIHETV ASWCNKNKCHVITQNIDNLDIKAGNKYVIELHGNINRNYCLLCGK

	FYDLAQLIHQKDKDGIPTCKCGGVINPDVVLYEDPLMEDSTNDAA<br>EAISNSDLLIIAGTSLSVYPAASYIHFYQGKRIVILNKDTSRYENSNN<br>ENILLFNENMKDVFENLANS
Bifidobacterium BL1528	MSVYDIDLFLRNKEDREYSWRWQKESPVWNAQPGTAHKALVKL<br>EQAGMLTLLATQNFDALHEKAGNSDNVIVNLHGTIGTSHCMKCH<br>QEYATADIMARLDEEPDPHCHRKLKYRGDMPCNGIIKTDVVYFGE<br>ALPDGAMEKSYSLATKADELWVIGSTLEVYPAASIVPVAAQAGVPI<br>TIMNMGHTQYDHLASRLIHEDIAVALPKLVDETIAENK
Mycolicibacterium Rv1151c*	MRVAVLSGAGISAESGVPTFRDDKNGLWARFDPYELSSTQGWLRN<br>PERVWGWYLWRHYLVANVEPNDGHRAIAAWQDHAEVSVITQNV<br>DDLHERAGSGAVHHLHGSLFEFRCARCGVPYTDALPEMPEPAIEV<br>EPPVCDCGGLIRPDIVWFGEPLPEEPWRSAVEATGSADVMVVVGT<br>SAIVYPAAGLPDLALARGTAVIEVNPEPTPLSGSATISIRESASQALP<br>GLLERLPALLK
Mycolicibacterium<br>MSMEG_4620*	MDAPELVALLQGRRIVALTGAGMSTDSGIPDYRGPDSPPSNPMTIQ<br>QFTSDPVFRQRYWARNHVGWRHMDETQPNAGHRALAAMEASG<br>VVAGVITQNVDLLHTKAGSREVINLHGTYAQVVCLNPDCGHTMS<br>RAALAVMLEEANPGFLARAESVGGIAVAPDADAMITDTASFVVVD<br>CPMCGGMLKPDIVYFGDSVPKTRVEQAYSLVDSADALLVAGSSLT<br>VFSGYRFVRHAAARGIPVGIVNRGPTRGDDLAAVKVHSGCSEMLT<br>LLAGELTRTYTASPG
Mycolicibacterium<br>MSMEG_5175*	MQVTVLSGAGISAESGVPTFRDAETGLWAQVDPYEISSTDGWQRN<br>PEKVWAWYLWRHYMMARVAPNEAHRTVAAWEDHLDVRVVTQNI<br>DDLHERAGSTNVYHLHGSLFEFRCDACGSAFEGNLPEMPEPVETI<br>DPPVCPCSGLIRPSVVWFGEPLPDAAWNRSVLAVSSADVVIVVGTS<br>SIVYPAAGLPEAALAAGKPVIEVNPERTPLSDSATVSLRETASEALP<br>TLLQRLPELLNRSA
Streptomyces SCO6464*	MTGKPLVAILSGAGVSTDSGIPDYRGPNGLWRRDPEAEKLVTYEY<br>YMGDPEIRRRSWLMRRDSAALHAEPNAAHRAVADLERRGVPVRV<br>LTQNVDGLHQLAGVSARKVLELHGTARDCVCTGCGARGPMADV<br>LARIEAGEDDPPCLDCGGVLKTATVMFGERLDPVVLGEAAAISKA<br>CQVFVAVGTSLQVEPAAGLARVAVEHGARLVVVNAEPTPYDELAD<br>EVIREPIGSALPALLRGLG
Streptomyces SCO0452*	MRMRPTLSWTPGADLPPGTTDLAPVADALRAGGVLVLSGAGISTE<br>SGIPDYRGEGGSLSRHTPMTYQDFTAHPEARRRYWARSHLGWRTF<br>GRARPNAGHRSVAAFGRHGLLTGVITQNVDGLHQAAGSEGVVEL<br>HGSLDRVVCLSCGVLSPRRELARRLEEANAGFSPVAAGINPDGDA<br>DLTDEQVGDFRVVPCAVCGGVLKPDVVFFGENVPPRRVEHCRELV<br>RGASSLLVLGSSLTVMSGLRFVRQAAEAGKPVLIVNRDATRGDRL<br>AVTRVALPLGPALTTVADRLGLRVGDAATA
Nocardia NFA_43380	MTQLAPSPLGTGVDRLIDLLAGLRVAVLTGAGLSTDSGIPDYRGPD<br>SPPRNPMTYQQFVGDPVFRRRYWARNHIGWRRMDAARPNPGHR<br>ALARLERLGVVGGVITQNVDLLHTKAGSRRVIDLHGTYARVRCLG<br>CGALMSRMTLADLLEAANPGFADAATATGIEVAPDADAVVADTEH

	FRMVDCAHCGGMLKPDIVYFGENVPKDRVAAAYDLVDSCDALLV<br>AGSSLTVMSGLRFVRRAARNGHPVVIVNRGPTRGDDLATLRLDA<br>GCSPTLAAFAAHCGDTPG
Nocardia NFA_44580	MTRQWRTRSGRIGVLTGAGISTDSGIPDFRGPRGVWTEDPIAELMS<br>TYDQYLSDPDLRRRSWLARRANPAWQAEPNAGHLALVDLERAGR<br>AVTIITQNVDRLHQRAGSSPQRVVEIHGNMFEVVCVGCDYETGMA<br>DVLARVEAGEPDPACPECGGILKAATIMFGQQLDQRTMTKAALTA<br>QTSDIFLAVGTSLQVEPAASMCALAVDAGADLVIVNAEPTPYDSIA<br>TEVVHEPIGTALPRLVKEILDA
Corynebacterium NCgl0078	MVELEQHGFLSGVVTQNVDGLHAEAGTKNLVALHGDLAHVMCL<br>NCGFGEDRHLFDERLEAANPGYVASIRLEPGAVNPDGDVFLDEEQ<br>VRRFTMIGCLRCGSLMLKPDVVYFGEPVPAARKKDLKKLLDASSS<br>LLIAGSSLAVMSGYRIVIEAQRQGKQVSVINGGPGRADSRVDILWR<br>TRVAPAFDDILDALDL
Corynebacterium NCgl0616	MSERQLEKSIEHAVELAREARNIEVFTGAGMSADSGLETYRDDKT<br>GLWSNVDPQAMASIDAWRKDPEPMWAWYRWRAGVAARAEPNA<br>GHQAISYWEGSDTVEHVHITTQNIDNLHERAGSSDVTHLHGSLFE<br>YRCSDCATPWEDDKNYPQEPIARLAPPQCEKCGGLIRPGVVWFGE<br>NLPVEEWDIAEQRIAEADLMIIVGTSGIVHPAAALPQLAQQRGVPI<br>VEISPTRTELSRIADFTWMSTAAQALPALMRGLSA
Amycolatopsis AMED5937*	MDGKLIDRIRGAWRVAALTGAGISTASGIPDYRGPEGVWTRTPSAV<br>NAFTLENFMADADVRREFWRTYAGHAAWRAEPNAAHRALAELD<br>GAGVAVRVLTQNVDGLHQRAGLAARKVLELHGTMHTTRCTGCA<br>AGFPTAEILEAGDDDPSCPRCGGILKLDIVLFGQRLDGDILGQARNI<br>AAASELFLAIGSSLQVEPAASLCTVAVGAGATLVVVNRDPTPYDDD<br>ADFVLRDDIEAVVPELCAAVNR
Amycolatopsis AMED4209*	VRTRPTLSWTSADAPLPRTSSLDELTSVVARGRVAVLSGAGLSTES<br>GIPDYRGESGSLRRHTPMTYDEFVTSAEGRQRYWARSHLGWRTIA<br>RADPNDGHRAVATLRDGGYVSGVITQNVDGLHQAAGTADAVELH<br>GSLDRVVCLDCRRTSPRAELDRRLRAANPGFIGAATRINPDGDVEL<br>PADVVRAFRPVPCAACAGVLKPDVVFFGENVPRPRVEQCYRLVD<br>DAEALLVLGSSLTVMSGLRFVRHAANAGKPVVIVNRGETRGDRY<br>ASVRVDRPLGPALTELVSRLREDRGRTA
Amycolatopsis AMED8708*	VRELAAKLLDGAGALLICAGAGMGVDSGLPDFRGSEGFWRAYPP<br>YARLGLRFEELADPRHFADDPELAWGFYGHRLELYRKTVPHEGFR<br>LLREFGSDLPGGVRVFTSNVDGQFQAAGFGFVAEAHGSIHHLQCL<br>AGCTADIWPAAGVTVAIAPETMRAVPPLPSCPRCGGLARPNILMFG<br>DFSWVPDRSQAQLDELTAWRRTARDLVVVELGAGQAVPTVRRYA<br>ELASAATGALIRINPREPEIRHGRGVSIAAGALETLTALTAARPRNPA<br>S
Leishmania LMJF_34_2140	MKACRCITILTGAGISAESGISTFRDSNGLWCNHHVEDVASPDAFIR<br>NPALVQLFYNERRRNLLLSSVKPNKAHTALAKLEEELSGKGKVFIV<br>TQNVDNLHERAGSKNVLHMHGELLKARCTATGNVFEWQKDIVG<br>DVDRCPDCGFLGTLRPHIVWFGEMPLCMDEIESILSTTDLFVAIGTS

	GNVYPAAGFVKRAQFYGATTLELNLQEGSNSTLFQESIYGKASSIV<br>PTWVDQVLKESLKK
Leishmania LMJF_26_0210	MTGSPRAPHQEHALGEPTVEGLARYIREKDVRRILVLVGAGASVA<br>AGIPDFRSSDTGIYAKLGKYNLDDPTDAFSLTLLREKPEIFYSIAREL<br>NLWPGHFQPTAVHHFIRLLQDEGRLLRCCTQNIDGLEKAAGVSPEL<br>LVEAHGSFAAAACIECHTPFSIEQNYLEAMSGTVSRCSTCGGIVKP<br>NVVFFGENLPDAFFDALHHDAPIAELVIIIGTSMQVHPFALLPCVVP<br>KSIPRVLMNRERVGGLLFRFPDDPLDTIHDDAVAKEGRSSSSQSRSP<br>SASARREEGGTEDGSSSPNEEVEDASTSSSSDGYGQYGDYYAHPD<br>VCRDVFFRGDCQENVLKLAECLGLREALAKRMRFSGAAPATARK<br>TSNET
Leishmania LMJF_23_1210	MRPAGTLASFLERCSARKRGRGCVVLTGAGCSTESGIPDYRGPNG<br>QYHRADFVLLTFQKFMRDDNEKRRYWARSMLGYSTMCGASCNA<br>AHMALQAFTKSGAVAHILTQNVDGLHHLATYGGVGDAEEEHYYK<br>YTTSDAPLKELHGNIHNVICTSCGFFMPRARLQRELRERNPGFYEQ<br>YGADVSRTRPDGDYSAPTEAVNAMHLVMCPRCNGFFKPHVVLFG<br>ENVPKPIVEATMSLVRDKASCLLCLGTSLQVYSAYRYVLQANQLG<br>IPVAIVNAGTTRGDAIADLKLDVESVGSVLAETAHEMLGVPASMFF<br>RRKTIQL
Saccharomyces YDL042C	MTIPHMKYAVSKTSENKVSNTVSPTQDKDAIRKQPDDIINNDEPSH<br>KKIKVAQPDSLRETNTTDPLGHTKAALGEVASMELKPTNDMDPLA<br>VSAASVVSMSNDVLKPETPKGPIIISKNPSNGIFYGPSFTKRESLNA<br>RMFLKYYGAHKFLDTYLPEDLNSLYIYYLIKLLGFEVKDQALIGTI<br>NSIVHINSQERVQDLGSAISVTNVEDPLAKKQTVRLIKDLQRAINK<br>VLCTRLRLSNFFTIDHFIQKLHTARKILVLTGAGVSTSLGIPDFRSSE<br>GFYSKIKHLGLDDPQDVFNYNIFMHDPSVFYNIANMVLPPEKIYSP<br>LHSFIKMLQMKGKLLRNYTQNIDNLESYAGISTDKLVQCHGSFATA<br>TCVTCHWNLPGERIFNKIRNLELPLCPYCYKKRREYFPEGYNNKV<br>GVAASQGSMSERPPYILNSYGVLKPDITFFGEALPNKFHKSIREDIL<br>ECDLLICIGTSLKVAPVSEIVNMVPSHVPQVLINRDPVKHAEFDLSL<br>LGYCDDIAAMVAQKCGWTIPHKKWNDLKNKNFKCQEKDKGVY<br>VVTSDEHPKTL
Saccharomyces YOL068C*	MNILLMQRIVSFILVVSQGRYFHVGELTMTMLKRPQEEESDNNAT<br>KKLKTRLTYPCILGKDKVTGKFIFPAITKDDVMNARLFLKDNDLK<br>TFLEYFLPVEVNSIYIYFMIKLLGFDVKDKELFMALNSNITSNKERS<br>SAELSSIHAKAEDEDELTDPLEKKHAVKLIKDLQKAINKVLSTRLR<br>LPNFNTIDHFTATLRNAKKILVLTGAGVSTSLGIPDFRSSEGFYSKIR<br>HLGLEDPQDVFNLDIFLQDPSVFYNIAHMVLPPENMYSPLHSFIKM<br>LQDKGKLLRNYTQNIDNLESYAGIDPDKLVQCHGSFATASCVTCH<br>WQIPGEKIFENIRNLELPLCPYCYQKRKQYFPMSNGNNTVQTNINF<br>NSPILKSYGVLKPDMTFFGEALPSRFHKTIRKDILECDLLICIGTSLK<br>VAPVSEIVNMVPSHVPQILINRDMVTHAEFDLNLLGFCDDVASLVA<br>KKCHWDIPHKKWQDLKKIDYNCTEIDKGTYKIKKQPRKKQQ
Saccharomyces YPL015C	MSVSTASTEMSVRKIAAHMKSNPNAKVIFMVGAGISTSCGIPDFRS

	PGTGLYHNLARLKLPYPEAVFDVDFFQSDPLPFYTLAKELYPGNFR<br>PSKFHYLLKLFQDKDVLKRVYTQNIDTLERQAGVKDDLIIEAHGSF<br>AHCHCIGCGKVYPPQVFKSKLAEHPIKDFVKCDVCGELVKPAIVFF<br>GEDLPDSFSETWLNDSEWLREKITTSGKHPQQPLVIVVGTSLAVYP<br>FASLPEEIPRKVKRVLCNLETVGDFKANKRPTDLIVHQYSDEFAEQ<br>LVEELGWQEDFEKILTAQGGMGDNSKEQLLEIVHDLENLSLDQSE<br>HESADKKDKKLQRLNGHDSDEDGASNSSSSQKAAKE
Saccharomyces YOR025W*	MTSVSPSPPASRSGSMCSDLPSSLQTEKLAHIIGLDADDEVLRRVT<br>KQLSRSRRIACLTGAGISCNAGIPDFRSSDGLYDLVKKDCSQYWSI<br>KSGREMFDISLFRDDFKISIFAKFMERLYSNVQLAKPTKTHKFIAHL<br>KDRNKLLRCYTQNIDGLEESIGLTLSNRKLPLTSFSSHWKNLDVVQ<br>LHGDLKTLSCTKCFQTFPWSRYWSRCLRRGELPLCPDCEALINKR<br>LNEGKRTLGSNVGILRPNIVLYGENHPSCEIITQGLNLDIIKGNPDFL<br>IIMGTSLKVDGVKQLVKKLSKKIHDRGGLIILVNKTPIGESSWHGII<br>DYQIHSDCDNWVTFLESQIPDFFKTQDQIKKLRQLKREASDLRKQ<br>MKAQKDSIGTPPTTPLRTAQGIDIQGNNELNTKIKSLNTVKRKILSP<br>ENSSEEDEEENLDTRKRAKIRPTFGDNQAS
Saccharomyces YDR191W*	MKQKFVLPITPPSTAEKKPQTENRCNENLKPRRLLPQLKKSVRNR<br>KPRLSYRPELNSVFDLDAYVDSTHLSKSQRHHMDRDAGFISYALN<br>YSKRMVVVSGAGISVAAGIPDFRSSEGIFSTVNGGSGKDLFDYNRV<br>YGDESMSLKFNQLMVSLFRLSKNCQPTKFHEMLNEFARDGRLLR<br>LYTQNIDGLDTQLPHLSTNVPLAKPIPSTVQLHGSIKHMECNKCLN<br>IKPFDPELFKCDDKFDSRTEIIPSCPQCEEYETVRKMAGLRSTGVGK<br>LRPRVILYNEVHPEGDFIGEIANNDLKKRIDCLIIVGTSLKIPGVKNI<br>CRQFAAKVHANRGIVLYLNTSMPPKNVLDSLKFVDLVVLGDCQH<br>VTSLL
Zea 103642296	MAVAPAAHAYRASASVEVFMQPTYTCNGTQMIAGLTGALSVAFR<br>GNNALLYRRENPLSLRCSFRSLKARNNHSSTVAPKDYCETYIQFLR<br>DKQIVPDSDPPSPKDVDLFYQFIDKSKRLMVVTGAGMSTESGIPDY<br>RSPNGAYSTGFKPLSHQEFVRSIRAQRRYWARSYAGWRRFRRAQP<br>NAAHYALASLERIGRVHSMVTQNVDRLHHRAGSNPLELHGSVYE<br>VICLECGTSISRESFQEEVKNLNLKWAQAIDSLEVGQPGSGKSFGM<br>QQRPDGDVEIDEKFWEQDFEIPSCHQCGGVLKPDVVMFGDNVPQ<br>ERAESAKEAARTCDALLVVGSALMTMSAFRLASSQTGLNYVSSMI<br>NPNXRLAHEANAPIAAVSIGETRADSILSLKINARCGEILPRILQMG<br>SLVVPDVN
Zea 542568	MSLGYAEKLSYREDVGTVGMPEIFETPELVQNKIEELAAMVQKSK<br>HLVVFTGAGISTSSGIPDFRGPMGVWTLQRAGKGIPNASLPFHHAV<br>PSLTHMALVELERAGFLKFVISQNVDSLHLRSGFPREKLAELHGNS<br>FKEICPCCKTEYLRDFEIETIGLKDTPRRCSDKNCGARLKDTVLDW<br>DDALPPEEMNLATEHCRSADLVLCLGTSLQITPACNMPLMSIKNG<br>GRVAIVNLQATPKDKKASLVIHGLVDKVIAGVMSKLSLRIPPYIRTD<br>FVQLTLRHSLKKKCVRWTLRVTSIHGLRAPLPFLQSVKVSFPERPD<br>LKSVVLKEQPFSLQRETSMNKPFFMLLTLNFSDGCSCLSSSIGWPV

	DFQKRKDSFVRDRALVLRELYSAAQRESCIGQQEILERENLPRAET SIHGIVTNIVRYDTEDEKLAPPKNDLMNHSRSNPAKRHVEGTDCH SSLPKKLKYFLKDKKLNC
Oryza 4351669*	MAAGAHASRASAPIIAGLTGALRAAYKGFSPQLCNFHASVNNGLL HRRKIQLHFICSFRSIQARYNHSSAVAPKDYCETYIQFLRDKQIVPD SDPPSAKDVDLLYRFIDQSKKLMVLTGAGMSTESGIPDYRSPNGAY SSGFKPLTHQEFVRSIRARRRYWARSYAGWRRFRRAQPNSAHYAL ASLERIGRVHSMVTQNVDRLHHRAGSKPVELHGSVYEVACLDCG TSIDRESFQEQVKDLNPKWALAIDSLEVGQPGSDKSFGMQQRPDG DIEIDEKFWEQDFDIPSCNQCGGVLKPDVVMFGDNVPEERAESTK EAARNCDALLVVGSALMTMSAFRLARLAHEANAPIAAITIGETRA DSILSLKINARCGEILPRILQMGSLAVPNVS
Oryza 4335342*	MSLGYAEKLSYREDVGNVGMPEIFDSPELLHKKIEELAVMVRESK HLVVFTGAGISTSSGIPDFRGPKGVWTLQRSGKGVPGASLPFHRAV PTLTHMALVELEKTGRLKFVISQNVDSLHLRSGLPREKLAELHGNS FKEICPSCKKEYLRDFEIETIGLKDTPRRCSDKNCGARLKDTVLDW EDALPPEEMDAAKEQCQKADLVLCLGTSLQITPACNMPLLSLKNG GRVAIVNLQATPKDKKASLVIHGLVDKVIAGVMYMMNLRIPPYIRT DFVQISLRNSVKKKCVRWTLRVTSIHGLRAPLPFLRSVEVSFPERP DMKPVVLKEQPFSLQRETSMNRPFVMLLTFNFSDGCGCSSSSIEWP VDFLKQKDSFVRDRSLVLQELQHAAEHRSRAGQHAILEREGVPRA ETSIHALVTNIVRYDTEDSKAAVPMATWMNSNGSLSKRHMDAIGC NPASSKKQKLVATRHRRKGLNPATQKV
Arabidopsis AT5G55760	MSLGYAEKLSFIEDVGQVGMAEFFDPSHLLQCKIEELAKLIQKSKH LVVFTGAGISTSCGIPDFRGPKGIWTLQREGKDLPKASLPFHRAMP SMTHMALVELERAGILKFVISQNVDGLHLRSGIPREKLSELHGDSF MEMCPSCGAEYLRDFEVETIGLKETSRKCSVEKCGAKLKDTVLD WEDALPPKEIDPAEKHCKKADLVLCLGTSLQITPACNLPLKCLKGG GKIVIVNLQKTPKDKKANVVIHGLVDKVVAGVMESLNMKIPPYVR IDLFQIILTQSISGDQRFINWTLRVASVHGLTSQLPFIKSIEVSFSDNH NYKDAVLDKQPFLMKRRTARNETFDIFFKVNYSDGCDCVSTQLSL PFEFKISTEEHVEIIDKEAVLQSLREKAVEESSCGQSGVVERRVVSE PRSEAVVYATVTSLRTYHSQQSLLANGDLKWKLEGSGTSRKRSRT GKRKSKALAEETKA
Arabidopsis AT5G09230	MLSMNMRRVFGGVSTDLFPSRSMYRPLQSGGNLVMLFKGCRRFV RTTCRVSIPGGSLGNESKAPPRFLRDRKIVPDADPPNMEDIHKLYRL FEQSSRLTILTGAGVSTECGIPDYRSPNGAYSSGFKPITHQEFTRSSR ARRRYWARSYAGWRRFTAAQPGPAHTALASLEKAGRINFMITQNV DRLHHRAGSDPLELHGTVYTVMCLECGFSFPRDLFQDQLKAINPK WAEAIESIDHGDPGSEKSFGMKQRPDGDIEIDEKFWEEGFHIPVCE KCKGVLKPDVIFFGDNIPKERATQAMEVAKQSDAFLVLGSSLMTM SAFRLCRAAHEAGAMTAIVNIGETRADDIVPLKINARVGEILHRVL DVGSLSVPAL
Caenorhabditis	MSRDSGNDSEVAVTHGEVQEITEENPEIGSMHITQETDISDAPETNT

CELE_R11A8.4	DSSRQRTESTTSVSSESWQNNDEMMSNLRRAQRLLDDGATPLQII<br>QQIFPDFNASRIATMSENAHFAILSDLLERAPVRQKLTNYNSLADA<br>VELFKTKKHILVLTGAGVSVSCGIPDFRSKDGIYARLRSEFPDLPDP<br>TAMFDIRYFRENPAPFYNFAREIFPGQFVPSVSHRFIKELETSGRLLR<br>NYTQNIDTLEHQTGIKRVVECHGSFSKCTCTRCGQKYDGNEIREE<br>VLAMRVAHCKRCEGVIKPNIVFFGEDLGREFHQHVTEDKHKVDLI<br>VVIGSSLKVRPVALIPHCVDKNVPQILINRESLPHYNADIELLGNCD<br>DIIRDICFSLGGSFTELITSYDSIMEQQGKTKSQKPSQNKRQLISQED<br>FLNICMKEKRNDDSSDEPTLKKPRMSVADDSMDSEKNNFQEIQKH<br>KSEDDDDTRNSDDILKKIKHPRLLSITEMLHDNKCVAISAHQTVFP<br>GAECSFDLETLKLVRDVHHETHCESSCGSSCSSNADSEANQLSRA<br>QSLDDFVLSDEDRKNTIHLDLQRADSCDGDFQYELSETIDPETFSH<br>LCEEMRI
Caenorhabditis<br>CELE_F46G10.3*	MARKYVPHTTELCENSLKKFKSLVGTVDKLLIITGAGISTESGIPDY<br>RSKDVGLYTKTALEPIYFQDFMKSKKCRQRYWSRSYLNWPRFAQ<br>ALPNFNHYALSKWEAANKFHWLITQNVDGLHLKAGSKMITELHG<br>NALQVKCTSCEYIETRQTYQDRLNYANPGFKEQFVSPGQQELDAD<br>TALPLGSEQGFKIPECLNCGGLMKTDVTLFGENLNTDKIKVCGKK<br>VNECNGVLTLGTSLEVLSGYQIVNHAHMQNKPIFIVNIGPTRADQ<br>MATMKLDYRISDVLKEM
Caenorhabditis<br>CELE_F46G10.7*	MMKYGMAQKFVPEAAELCENSLKKFISLIGTVDKLLVISGAGISTE<br>SVPGIPDYRSKDVGLYARIAHKPIYFQDYMRSNRCRQRYWSRNFL<br>AWPRFGQAAPNINHYALSKWEASDRFQWLITQNVDGLHLKAGSK<br>MVTELHGSALQVKCTTCDYIESRQTYQDRLDYANPGFKEEHVAPG<br>ELAPDGDIILPLGTEKGFQIPECPSCGGLMKTDVTFFGENVNMDKV<br>NFCYEKVNECDGILSLGTSLAVLSGFRFIHHANMKKKPIFIVNIGPT<br>RADHMATMKLDYKISDVLKEM
Caenorhabditis<br>CELE_C06A5.11*	MTSVYESLLSDYPDKGVIGKPEIRDTETEIIEKLRTLYNHFVQAKQT<br>GKPIFVLIGAGVSTGSKLPDFRGKQGVWTLQAEGKHAEGVDFQV<br>ARPGVSHKSILALHKAGYIKTIITQNVDGLDRKVGIPVEDLIEVHG<br>NLFLEVCQSCFSEYVREEIVMSVGLCPTGRNCEGNKRTGRSCRGK<br>LRDATLDWDTEISLNHLDRIRKAWKQTSHLLCIGTSLEIIPMGSLPL<br>DAKSKGIKTTTINYQETAHEKIVETAIHADVKLILYSLCNALGVNV<br>DLGDDLPDEVPIPLKIS
Drosophila Dmel CG5216	MMENYEEIRLGHIRSKDLGNQVPDTTQFYPPTKFDFGAEILASTST<br>EAEAEAEATATTTEPATSELAGKANGEIKTKTLAAREEQEIGANLE<br>HKTKNPTKSMGEDEDDEEEEEEDDEEEEEDDEEGITGTSNEDEDS<br>SSNCSSSVEPDWKLRWLQREFYTGRVPRQVIASIMPHFATGLAGDT<br>DDSVLWDYLAHLLNEPKRRNKLASVNTFDDVISLVKKSQKIIVLT<br>GAGVSVSCGIPDFRSTNGIYARLAHDFPDLPDPQAMFDINYFKRDP<br>RPFYKFAREIYPGEFQPSPCHRFIKMLETKGKLLRNYTQNIDTLERV<br>AGIQRVIECHGSFSTASCTKCRFKCNADALRADIFAQRIPVCPQCQP<br>NKEQSVDASVAVTEEELRQLVENGIMKPDIVFFGEGLPDEYHTVM<br>ATDKDVCDLLIVIGSSLKVRPVAHIPSSIPATVPQILINREQLHHLKF

	DVELLGDSDVIINQICHRLSDNDDCWRQLCCDESVLTESKELMPPE HSNHHLHHHLLHHRHCSSESERQSQLDTDTQSIKSNSSADYILGSA GTCSDSGFESSTFSCGKRSTAAEAAAIERIKTDILVELNETTALSCD RLGLEGPQTTVESYRHLSIDSSKDSGIEQCDNEATPSYVRPSNLVQE TKTVAPSLTPIPQQRGKRQTAAERLQPGTFYSHTNNYSYVFPGAQV FWDNDYSDDDDEEEERSHNRHSDLFGNVGHNYKDDDEDACDLN AVPLSPLLPPSLEAHIVTDIVNGSNEPLPNSSPGQKRTACIIEQQPTP AIETEIPPLKKRRPSEENKQQTQIERSEESPPPGQLAAV
Drosophila Dmel CG5085	MDKVRRFFANTLHLGGSSDAKEEVKVEKVIPDLSFDGFAEHWRV HGFRKIVTMVGAGISTSAGIPDFRSPGSGLYSNLKKYELPHPTAIFD LDYFEKNPAPFFALAKELYPGSFIPTPAHYFIRLLNDKGLLQRHYTQ NIDTLDRLTGLPEDKIIEAHGSFHTNHCIKCRKEYDMDWMKAEIFA DRLPKCQKCQGVVKPDIVFFGENLPKRFYSSPEEDFQDCDLLIIMG TSLEVQPFASLVWRPGPRCIRLLINRDAVGQASCVLFMDPNTRSLL FDKPNNTRDVAFLGDCDAGVMALAKALGWDQELQQLITSERKKL SGSQNSEELQQGKEKPQSDPDKMTSGDRDKKDASL
Drosophila Dmel CG3187	MRVGQLLRFRSTSLRSSTARQEYVPHHKPVVEDDIKRLEDFLLSKP NVLVLTGAGISTESGIPDYRSEGVGLYARSNHKPVQHMEFVKSSAV RKRYWARNFVGWPKFSATQPNATHHALARFEREERVQAVVTQNV DRLHTKAGSRNVVEVHGSGYVVKCLSCEYRIDRHEFQSILASLNP AFKDAPDMIRPDGDVEIPLEYIENFRIPECTQCGGDLKPEIVFFGDS VPRPRVDQIAGMVYNSDGLLVLGSSLLVFSGYRVVLQTKDLKLPV GIVNIGETRADHLADIKISAKCGDVIPKLFDFRNSKSVS
Drosophila Dmel CG6284	MSCNYADGLSAYDNKGILGAPESFDSDEVVAEKCQELAELIKKSG HVVLHTGAGISTSAGIPDFRGPKGVWTLEEKGEKPDFNVSFDEAR PTKTHMAIIALIESGYVQYVISQNIDGLHLKSGLDRKYLSELHGNIY IEQCKKCRRQFVSPSAVETVGQKSLQRACKSSMDSKGRSCRSGILY DNVLDWEHDLPENDLEMGVMHSTVADLNIALGTTLQIVPSGDLPL KNLKCGGKFVICNLQPTKHDKKANLIISSYVDVVLSKVCKLLGVEI PEYSEASDPTKQSKPMEWTIPTSNVNTFHRQYKKYVKDSKIESKA KKTKYT
Drosophila Dmel CG11305	MEKDLGEEKDQDQEQNTEMEPKQEMDVAQSYITRAKMNPAKKD NEKRRRKDAMRRVSMILRKCDSMRTTEDRQFLEKHPDMVKTTKK RKERVEIYKERVVEREDAPHVIEAKVEQLANIISQAKHLVCYTGAG ISTAALIPDYRGSQGIWTLLQKGQDIGEHDLSSANPTYTHMALYEL HRRRLLHHVVSQNCDGLHLRSGLPRNSLSEIHGNMYVEVCKNCR PNSVYWRQFDTTEMTARYCHKTHRLCHRCSEPLYDTIVHFGERGN VKWPLNWAGATANAQRADVILCLGSSLKVLKKYTWLWQMDRPA RQRAKICVVNLQWTPKDAIASIKINGKCDQVMAQLMHLLHIPVPV YTKEKDPIFAHASLLMPEELHTLTQPLLKNADEEEAFTTTTEETQD STISSESCSFNYSDLPIGKGPRIRTPIKNGRRVKTNLELRQKFKTLNG QDEEIKVEHVKTNGEVKTEKDLITLESSIKIETEVKLEKLECSDTNF QQELKLLELLPKLEPLSLKEETEETPSNGFPELPKLVAIQKTHAECL SAVPTESRLKPLQLPPLVPIGAPLSTPFVEPKLVLPPASQSSSIQIKSE

	GDGDSSTENDNEEEEESELAQMDLLRQNNDEELLRQLPTWYDAK YAYSGLHSILIPPPADLNIWNSQVVPNFAMNRSAASCFFCFDRYAEL ECQFYRRWNLSQRKHKKRARSGRFVVCECCPTSDDDDDYDENIS LAHIAAAETAKRRQQLSTSFPRKLARTQAGWYGKGYKKGRKRR
Pygoscelis 103918614	YICAIWLKCNPSLLCCLADNFLFSDEIIANGFHSCDSDEDDRASHA SSSDWTPRPRIGPYTFVQQHLMLGTDPRTILKDLLPETIPPPELDDM TLWQIVINILSEPPKRKKRKDINTIDDAVKLLQECKKIMVLTGAGVS VSCGIPDFRSRDGIYARLAVDFPDLPDPQAMFDIEYFRKDPRPFFKF AKEIYPGQFQPSLCHKFIALMDKEGKLLRNYTQNIDTLEQVAGIQR IIQCHGSFATASCLICKYKVDCEVVRGDIFNQVVPRCPRCPPDELLA IMKPDIVFFGENLPEQFHRAMKYDKNEVDLLIVIGSSLKVRPVALIP SSIPHEVPQILINREPLPHLHFDVELLGDCDVIINELCQRLGSEYTKL CYNSVKLSEITEKPPRTHKELEIHSAELPPTPLNISEDSSSPERMTPP DTSVVSSEHPAECKLENCDPASETKGTCTEEKLQDTQTSSENPENP TNELMNSETMKENGSNNGENKEKNEILKKCWVNRSAKEQISKRL DGTQYLFLPPNRYIFHGAEVYSDSEDDIVSSSSCGSSSESGSCRSQS LDVEDESEIEEFYNGIEDEDAPEREEEPGFGEDGVEQDESAADESA YANEAAGTDHPSNKL
Pygoscelis 103925062	MGRWGDDGGKQKLTLQDVAELIRKKECRRVVVMAGAGISTPSGI PDFRSPGSGLYSNLEQYNIPYPEAIFELMYFFVNPKPFFTLAKELYP GNYRPNYAHYFLRLLHDKGLLLRLYTQNIDGLERVAGIPPNRLVEA HGTFATATCTVCRRKFPGEDFRGDVMADKVPHCPVCTGVVKPDIV FFGEELPQRFFLHVTDFPTADLLFVIGTSLEVEPFASLAGAVRSSVP RVLINRDLVGPFAWQQRYNDIAQLGDVIGGVEKLVELLDWNEEM QTLMQKEKAKLDAKDK
Pygoscelis 103922636	MFSAMKLSGVCRAIRFHHFRCHSLSRTSPNLAFVPACLPPDPVEVE ELQRFVSNSKRLFVMTGAGISTESGIPDYRSEGVGLYARTDRRPIQ HAEFVRSASARQRYWARNFVGWPQFSSHQPNTAHLVLRDWEKLG KLHWLVTQNVDALHTKAGSQRMTELHGCTHRVFCLVCGDQILRS ELQEHFEALNPTWKAEAFGVAPDGDVFLTDEQVCNFQVPACRKC GGILKPDVTFFGDTVSREKVNFVHQRLAESDSMLVAGSSMQVYSG YRFALAAREKQLPIAILNIGPTRLDHFASLKLNSRCGELLPLIVAX
Pygoscelis 103919047	MSLFQFTARRLVSQAYCGLKAAASSKKQKVRLEMARPSSDMADF REVFAKAKHIAIITGAGVSAESGVPTFRGAGGFWRKWQAQELATP EAFARNPSRVWEFYHYRREVMLSKHPNPAHIAIAECERRLSKQGR SVVVITQNIDELHRKAGTKHLLEIHGSLFKTRCTNCGNVAANYKSP ICPALAGKGAPDPETEDATIPVEDLPQCEEDGCNGLLRPHVVWFGE TLDPDILTEVEKELEICDLCLVVGTSSVVYPAAMFAPQVSARGVPV AEFNMEATPATNRFSKKRGIVLSWWN
Pygoscelis 103916494	MKSCARRQKAGWAHGPIQPRRPRVPHLRGFPCSSPSLGPVPSAELV AVLVTAGVEHRWGGTGFTPVLPLPGGVLEPSHFSGYQLPIFDPPEE LERKVRELADLIRSSSNVVFHTGAGISTASGIPDFRGPNGVWTMEE KGLSPKFDTTFENARPSKTHMALLGLQRVGILKFLVSQNVDGLHV RSGFPRYCLSRPVSGSLTLSPAAATWLQYVRDAVVGSMGLKPTGR

	LCSVTKARGLRACRGKLRDTILDWEDSLPDRDLTLADEACRKAD LSVTLGTSLQIKPSGNLPLITKKRGGKLVIVNLQATKHDRQADLRI HAYVDDVMTKLMKHLGLEVPEWTGPVVVESAELTKPEQLFKFDP GARGLLKEEPLSRHNGTGGLCPDLGTTLVERRDSLRQEGPSPDTG PTTVKKMKGEPLLT
Pygoscelis 103917462	MTGNPAAVVNLLSLYSAARNVRVLPCLLGGSRAVAGAKPWLKGT RVVCDEPEELKRKATELAAAVRNAKHLVIYTGAGISTAASIPDYRG PNGIWTLLQKGRSIRATDLSEAEPTLTHMSIACLHKHNLVQHVVSQ NCDGLHLRSGLPRTAISELHGNMYIEVCTSCTPNREYVRVFDVTER TALHRHHTGRTCHKCGAQLRDTIVHFGEKGTLRQPLNWEAATEA ASKADVILCLGSSLKVLKKYPRLWCMSKPPTRRPKLYIVNLQWTP KDALAARKLHGRCDEVMRLLMAELGLEI
Danio 436878	MIVRQLWCSRGSTSHLCAAVRLNWRSPKMTRPSSDLTAFREHFAK AKHIAIITGAGVSAESGVPTFRGPGGFWRKWQAQDLATPEAFSRD PSLVWEFYHYRREVMRSKMPNPAHLAIAECEARLGQQGRSVVIIT QNIDELHHRAGSKHVYEIHGSLFKTRCMSCGEVKANHKSPICPAL DGKGAPDPNTKEARIPVELLPRCERKSCNGLLRPHVVWFGETLDS DILTAVERELEKCDLCLVVGTSSIVYPAAMFAPQVASRGVPVAEFN MECTPATQRFKYHFEGPCGSTLPPALERHESEAV
Danio 797132	MADGENKRAESAEPDEPLPKKPRLLELSGDSEHSATAGADTLDEK PARMDESQQALSINNNNNTRPTEPGQPADPEPEISELTDEGVHPNG FTSPDLLRDDDDCSSRASSSDWTPQPQIGSYRFIQQHIMRGTDPRAI LKDLLPETVLPPDLDDMTLWQIIINISEPPKRKKRKDINTLEDVVRL LNERKKILVLTGAGVSVSCGIPDFRSRDGIYARLAVDFPDLPDPQA MFDIDYFRRDPRPFFKFAKEIYPGQFQPSPCHRFISMLDKKGRLLR NYTQNIDTLEQVAGIQKIIQCHGSFATASCLICKHKVDCEAIREDIFN QVVPHCPRCPSDVPYAIMKPDIVFFGENLPEFFHRAMKQDKDEVD LLIVIGSSLKVRPVALIPSSIPHDVPQVLINREPLPHLNFDVELLGDC DVIVNELCHRLNGDFQQLCYNSSRLSEITEKPAAPEHTENTSADHS HADAEHIENTSADHSHADAEHIENTSADRDDAKHTENTPTDHAD AEHTKNTSADHANAEHTENTSAGHVNAEHIEHMSKDHANPKDD QSSLSVNEEELASPAAETHALDSTEISAHTERSKEADAVNTDDAAC VKDEENTDRLRVEMRRRCWRSRICQSPISKRLGASQYLFQAPNRY VFHGAEVYSSSEDESSSSCGSESDGSFQHEDSEVEENGAAMTDKE TDTETVQDSEHRRLQTHCTQHTQ
Danio 558775	MLYLNTFLPSVCRRCFAENLLWRRGLTTTQNLSRTKLVHQKTLSHF PHAQKGAAFLSQFIYCPAAFIKCGGTRGLFGGGRDNVHQQTLEDI AEKIRERKFKRIVVMAGAGISTPSGIPDFRSPGSGLYDNLQQYNLP YAEAIFEINYFHHNPNPFFALAKELYPGNYQPNLTHYFIRMLHDKE QLLRMYTQNIDGLERMAGIPPKMLVEAHGTFATATCTVCRRDYKG EELRDDIMAGTVPKCPTCKGIIKPDIVFFGEELPQHFFTYLTDFPIAD LLIVMGTSLEVEPFASLAGAVRGSVPRLLINRDLVGPFASGSQRHT DVAELGDVVNGVKKLVELLGWKQELEDLMNVGRDK
Danio 791628	MLLSCRYLPPPVAVGRCASTIQAGVRQFVPASGSFDSSALEQLQAFI

	SQASRLFVISGAGLSTESGIPDYRSEGVGLYARTNRRPMQHSEFVR SEKSRQRYWARNYVGWPQFSSHQPNSAHLALRDWEEKGKLHWL VTQNVDALHLKAGQQRLTELHGSTHRVVCLDCGELTPRAELQKR FTALNPGWEATACAVAPDGDVFLEEEQVLNFRVPACNACGGVLKP EVTFFGDVVNRNTVHFVHNKLAESDAVLVAGSSLQVFSGYRFLLA ASERKLPIAIVNIGATRADHLTDIRVSARCGEVLPAIKLS
Danio 415161	MSVNYAAGLSPYADKGICGLPETFDSPEELKTKVETLAQWIRESQ YMVVHSGAGISTSTGIPDFRGPNGVWTMEERGETPHFNTTFEDAR PSLTHMALLQMQRTGHLKYLISQNVDGLHVRSGFPRDRLSELHGN MFVEECEKCGKQYVRDTVVGVMGLKPTGRYCDVMRSRGLRSCR GKLISSILDWEDSLPDRDLNRADEASRRADLALTLGTSLQIKPSGD LPLLTKRTGGKLVIVNLQPTKHDKHAHLRIYGYVDDVMGQLMKL LGLDVPEWAGPTLCEDSGGDLDILPYGAWKKEVKIELKIEESNHT VSKKRKRKEQHAEEGYKNGVKVEEEMKEEGKESDSHVHTHT
Danio 322309	MSEEVSKRVEEEADTPGLEGQSDDSSDEGDASGDTEMDFLRSLFS RTLGLSPGDKVLDELTLDSVARYILSGKCKNIICMVGAGISTSAGIP DFRSPGTGLYANLQKYNLPYPEAIFQIDYFKKHPEPFFALARELYPG QFKPTVYHYFIKMLKDKGLLRRCYSQNIDTLERVAGLEGEDLIEAH GTFHTSHCVSFLCRKEYSMDWMKNQIFSEEIPKCDSCGSLVKPDIV FFGESLPSRFFTSMKADFPQCDLLIIMGTSLQVQPFASLVSRVSNRC PRLLINMEKTGQSEFGMGLFSFGGGMDFDSDKAYRDVAHLSTCD DGCMTLAELLGWKKELEEMVKREHALIDSKDAKKTDKEASQSSK SAVAEAEKTDKTE
Danio 100536119	MMLAMCVCVCVLVFCRVSYSAQSVLLFSVNDARNVCVCVCVCV CALIPLSSPDPGVPDALIPVRDLPRCEQKACDGLLRPHVVWFGETL DSHILTKVEKELETCDLCLVVGTAAVVYPAAMFGPQVASRGVPVA EFNTRPTVNTPRYRFHFSGRCADTLPVALAPHESED
Danio 557125	MSKARLSRDRRAASVGVSRVTRSSMMSPQDCERSRAPDPGLLDE LSLMSVSEQQASATRKGSSKPALSSPSGRSVSRGALETIGRLMKLG RVRNIVVVAGAGISTASGIPDFRTPGTGLYANLAKYDIPYPEAVFNI DYFSDNPHPFFSLAKELYPGHHRPNYVHYFIRMLHQKGLLLRMYT QNIDGLEKLCGIPDDKLVEAHGSFATAACHLCYTPYPAEEAKQAIM NGSVPICTFCAGAVKPNVVFFGEDLPEKYFQHAEDFPKADLLMIM GTSLKVGLIEPFASLINTVKSTVPRLLLNRDAVGPFERRPLRRADY MELGDLSESVRKLAEILGWHTEIQTLMNSHENGLYSYISSSGENSG DSETDSMH
Danio 796135	MDVRINSGVSARAERKEQEKAKIIQREKQRQTMKTISKILQKCESE WTEEERSMLQAHQDTVQELSRRQNRRHLLKRKQEEVFDDAENLK TKVKQLAEAVQRAKHLVIYTGAGISTAASIPDYRGPNGVWTQLQK GRSVSTSDLSQAEPTLTHMSIWMLHKMKMVQHVVSQNCDGLHL RSGLPRHALSELHGNMFIEVCDSCSPPREFIRLFDVTERTALHRHGT GRSCPHCRAELRDTIVHFGERGTLEQPLNWKGAAEAAQRADLILC LGSSLKVLKKYSCLWCMNRPASKRPKLYIVNLQWTPKDNLATLKI HGKCDAVMALLMEELALAVPVYSRLQDPIFSMAKPLSPQEQKSHS

	RKEIAPPSALEEVSQSAPPQGEGPAVQGGWFGRGYSKGRRKKKSS
Homo sapiens SIRT1*	MADEAALALQPGGSPSAAGADREAASSPAGEPLRKRPRRDGPGLE RSPGEPGGAAPEREVPAAARGCPGAAAAALWREAEAEAAAAGGE QEAQATAAAGEGDNGPGLQGPSREPPLADNLYDEDDDDEGEEEEE AAAAAIGYRDNLLFGDEIITNGFHSCESDEEDRASHASSSDWTPRP RIGPYTFVQQHLMIGTDPRTILKDLLPETIPPPELDDMTLWQIVINIL SEPPKRKKRKDINTIEDAVKLLQECKKIIVLTGAGVSVSCGIPDFRS RDGIYARLAVDFPDLPDPQAMFDIEYFRKDPRPFFKFAKEIYPGQF QPSLCHKFIALSDKEGKLLRNYTQNIDTLEQVAGIQRIIQCHGSFAT ASCLICKYKVDCEAVRGDIFNQVVPRCPRCPADEPLAIMKPEIVFF GENLPEQFHRAMKYDKDEVDLLIVIGSSLKVRPVALIPSSIPHEVPQ ILINREPLPHLHFDVELLGDCDVIINELCHRLGGEYAKLCCNPVKLS EITEKPPRTQKELAYLSELPPTPLHVSEDSSSPERTSPPDSSVIVTLLD QAAKSNDDLDVSESKGCMEEKPQEVQTSRNVESIAEQMENPDLK NVGSSTGEKNERTSVAGTVRKCWPNRVAKEQISRRLDGNQYLFLP PNRYIFHGAEVYSDSEDDVLSSSSCGSNSDSGTCQSPSLEEPMEDE SEIEEFYNGLEDEPDVPERAGGAGFGTDGDDQEAINEAISVKQEVT DMNYPSNKS
Homo sapiens SIRT2*	MAEPDPSHPLETQAGKVQEAQDSDSDSEGGAAGGEADMDFLRNL FSQTLSLGSQKERLLDELTLEGVARYMQSERCRRVICLVGAGISTSA GIPDFRSPSTGLYDNLEKYHLPYPEAIFEISYFKKHPEPFFALAKELY PGQFKPTICHYFMRLLKDKGLLLRCYTQNIDTLERIAGLEQEDLVE AHGTFYTSHCVSASCRHEYPLSWMKEKIFSEVTPKCEDCQSLVKP DIVFFGESLPARFFSCMQSDFLKVDLLLVMGTSLQVQPFASLISKAP LSTPRLLINKEKAGQSDPFLGMIMGLGGGMDFDSKKAYRDVAWL GECDQGCLALAELLGWKKELEDLVRREHASIDAQSGAGVPNPSTS ASPKKSPPPAKDEARTTEREKPQ
Homo sapiens SIRT3*	MAFWGWRAAAALRLWGRVVERVEAGGGVGPFQACGCRLVLGG RDDVSAGLRGSHGARGEPLDPARPLQRPPRPEVPRAFRRQPRAAA PSFFFSSIKGGRRSISFSVGASSVVGSGGSSDKGKLSLQDVAELIRA RACQRVVVMVGAGISTPSGIPDFRSPGSGLYSNLQQYDLPYPEAIF ELPFFFHNPKPFFTLAKELYPGNYKPNVTHYFLRLLHDKGLLLRLY TQNIDGLERVSGIPASKLVEAHGTFASATCTVCQRPFPGEDIRADV MADRVPRCPVCTGVVKPDIVFFGEPLPQRFLLHVVDFPMADLLLIL GTSLEVEPFASLTEAVRSSVPRLLINRDLVGPLAWHPRSRDVAQLG DVVHGVESLVELLGWTEEMRDLVQRETGKLDGPDK
Homo sapiens SIRT4*	MKMSFALTFRSAKGRWIANPSQPCSKASIGLFVPASPPLDPEKVKE LQRFITLSKRLLVMTGAGISTESGIPDYRSEKVGLYARTDRRPIQHG DFVRSAPIRQRYWARNFVGWPQFSSHQPNPAHWALSTWEKLGKL YWLVTQNVDALHTKAGSRRLTELHGCMDRVLCLDCGEQTPRGVL QERFQVLNPTWSAEAHGLAPDGDVFLSEEQVRSFQVPTCVQCGG HLKPDVVFFGDTVNPDKVDFVHKRVKEADSLLVVGSSLQVYSGY RFILTAWEKKLPIAILNIGPTRSDDLACLKLNSRCGELLPLIDPC
Homo sapiens SIRT5*	MRPLQIVPSRLISQLYCGLKPPASTRNQICLKMARPSSSMADFRKFF

	AKAKHIVIISGAGVSAESGVPTFRGAGGYWRKWQAQDLATPLAFA<br>HNPSRVWEFYHYRREVMGSKEPNAGHRAIAECETRLGKQGRRVV<br>VITQNIDELHRKAGTKNLLEIHGSLFKTRCTSCGVVAENYKSPICPA<br>LSGKGCEEAGCGGLLRPHVVWFGENLDPAILEEVDRELAHCDLCL<br>VVGTSSVVYPAAMFAPQVAARGVPVAEFNTETTPATNRFRFHFQG<br>PCGTTLPEALACHENETVS
Homo sapiens SIRT6*	MSVNYAAGLSPYADKGKCGLPEIFDPPEELERKVWELARLVWQSS<br>SVVFHTGAGISTASGIPDFRGPHGVWTMEERGLAPKFDTTFESARP<br>TQTHMALVQLERVGLLRFLVSQNVDGLHVRSGFPRDKLAELHGN<br>MFVEECAKCKTQYVRDTVVGTMGLKATGRLCTVAKARGLRACR<br>GELRDTILDWEDSLPDRDLALADEASRNADLSITLGTSLQIRPSGN<br>LPLATKRRGGRLVIVNLQPTKHDRHADLRIHGYVDEVMTRLMKH<br>LGLEIPAWDGPRVLERALPPLPRPPTPKLEPKEESPTRINGSIPAGPK<br>QEPCAQHNGSEPASPKRERPTSPAPHRPPKRVKAKAVPS
Homo sapiens SIRT7*	MAAGGLSRSERKAAERVRRLREEQQRERLRQVSRILRKAAAERSA<br>EEGRLLAESADLVTELQGRSRRREGLKRRQEEVCDDPEELRGKVR<br>ELASAVRNAKYLVVYTGAGISTAASIPDYRGPNGVWTLLQKGRSV<br>SAADLSEAEPTLTHMSITRLHEQKLVQHVVSQNCDGLHLRSGLPR<br>TAISELHGNMYIEVCTSCVPNREYVRVFDVTERTALHRHQTGRTC<br>HKCGTQLRDTIVHFGERGTLGQPLNWEAATEAASRADTILCLGSS<br>LKVLKKYPRLWCMTKPPSRRPKLYIVNLQWTPKDDWAALKLHGK<br>CDDVMRLLMAELGLEIPAYSRWQDPIFSLATPLRAGEEGSHSRKSL<br>CRSREEAPPGDRGAPLSSAPILGGWFGRGCTKRTKRKKVT
Mouse SIRT1	MADEVALALQAAGSPSAAAAMEAASQPADEPLRKRPRRDGPGLG<br>RSPGEPSAAVAPAAAGCEAASAAAPAALWREAAGAAASAEREAPA<br>TAVAGDGDNGSGLRREPRAADDFDDDEGEEEDEAAAAAAAAAIG<br>YRDNLLLTDGLLTNGFHSCESDDDDRTSHASSSDWTPRPRIGPYTF<br>VQQHLMIGTDPRTILKDLLPETIPPPELDDMTLWQIVINILSEPPKRK<br>KRKDINTIEDAVKLLQECKKIIVLTGAGVSVSCGIPDFRSRDGIYAR<br>LAVDFPDLPDPQAMFDIEYFRKDPRPFFKFAKEIYPGQFQPSLCHKF<br>IALSDKEGKLLRNYTQNIDTLEQVAGIQRILQCHGSFATASCLICKY<br>KVDCEAVRGDIFNQVVPRCPRCPADEPLAIMKPEIVFFGENLPEQF<br>HRAMKYDKDEVDLLIVIGSSLKVRPVALIPSSIPHEVPQILINREPLP<br>HLHFDVELLGDCDVIINELCHRLGGEYAKLCCNPVKLSEITEKPPR<br>PQKELVHLSELPPTPLHISEDSSSPERTVPQDSSVIATLVDQATNNNV<br>NDLEVSESSCVEEKPQEVQTSRNVENINVENPDFKAVGSSTADKNE<br>RTSVAETVRKCWPNRLAKEQISKRLEGNQYLFVPPNRYIFHGAEV<br>YSDSEDDVLSSSSCGSNSDSGTCQSPSLEEPLEDESEIEEFYNGLED<br>DTERPECAGGSGFGADGGDQEVVNEAIATRQELTDVNYPSDKS
Mouse SIRT2	MAEPDPSDPLETQAGKVQEAQDSDSDTEGGATGGEAEMDFLRNL<br>FTQTLGLGSQKERLLDELTLEGVTRYMQSERCRKVICLVGAGISTS<br>AGIPDFRSPSTGLYANLEKYHLPYPEAIFEISYFKKHPEPFFALAKEL<br>YPGQFKPTICHYFIRLLKEKGLLLRCYTQNIDTLERVAGLEPQDLVE<br>AHGTFYTSHCVNTSCRKEYTMGWMKEKIFSEATPRCEQCQSVVK

	PDIVFFGENLPSRFFSCMQSDFSKVDLLIIMGTSLQVQPFASLISKAP LATPRLLINKEKTGQTDPFLGMMMGLGGGMDFDSKKAYRDVAW LGDCDQGCLALADLLGWKKELEDLVRREHANIDAQSGSQAPNPS TTISPGKSPPPAKEAARTKEKEEQQ
Mouse SIRT3	MALDPLGAVVLQSIMALSGRLALAALRLWGPGGGRRPISLCVGAS GGFGGGGSSEKKFSLQDVAELLRTRACSRVVVMVGAGISTPSGIPD FRSPGSGLYSNLQQYDIPYPEAIFELGFFFHNPKPFFMLAKELYPGH YRPNVTHYFLRLLHDKELLLRLYTQNIDGLERASGIPASKLVEAHG TFVTATCTVCRRSFPGEDIWADVMADRVPRCPVCTGVVKPDIVFF GEQLPARFLLHMADFALADLLLILGTSLEVEPFASLSEAVQKSVPR LLINRDLVGPFVLSPRRKDVVQLGDVVHGVERLVDLLGWTQELL DLMQRERGKLDGQDR
Mouse SIRT4	MSGLTFRPTKGRWITHLSRPRSCGPSGLFVPPSPPLDPEKIKELQRFI SLSKKLLVMTGAGISTESGIPDYRSEKVGLYARTDRRPIQHIDFVRS APVRQRYWARNFVGWPQFSSHQPNPAHWALSNWERLGKLHWLV TQNVDALHSKAGSQRLTELHGCMHRVLCLNCGEQTARRVLQERF QALNPSWSAEAQGVAPDGDVFLTEEQVRSFQVPCCDRCGGPLKPD VVFFGDTVNPDKVDFVHRRVKEADSLLVVGSSLQVYSGYRFILTA REQKLPIAILNIGPTRSDDLACLKLDSRCGELLPLIDPRRQHSDVQR LEMNFPLSSAAQDP
Mouse SIRT5	MRPLLIAPGRFISQLCCRRKPPASPQSKICLTMARPSSNMADFRKCF ANAKHIAIISGAGVSAESGVPTFRGAGGYWRKWQAQDLATPQAF ARNPSQVWEFYHYRREVMRSKEPNPGHLAIAQCEARLRDQGRRV VVITQNIDELHRKAGTKNLLEIHGTLFKTRCTSCGTVAENYRSPICP ALAGKGAPEPETQDARIPVDKLPRCEEAGCGGLLRPHVVWFGEN LDPAILEEVDRELALCDLCLVVGTSSVVYPAAMFAPQVASRGVPVA EFNMETTPATDRFRFHFPGPCGKTLPEALAPHETERTS
Mouse SIRT6	MSVNYAAGLSPYADKGKCGLPEIFDPPEELERKVWELARLMWQS SSVVFHTGAGISTASGIPDFRGPHGVWTMEERGLAPKFDTTFENAR PSKTHMALVQLERMGFLSFLVSQNVDGLHVRSGFPRDKLAELHG NMFVEECPKCKTQYVRDTVVGTMGLKATGRLCTVAKTRGLRAC RGELRDTILDWEDSLPDRDLMLADEASRTADLSVTLGTSLQIRPSG NLPLATKRRGGRLVIVNLQPTKHDRQADLRIHGYVDEVMCRLMK HLGLEIPAWDGPCVLDKALPPLPRPVALKAEPPVHLNGAVHVSYK SKPNSPILHRPPKRVKTEAAPS
Mouse SIRT7	MAAGGGLSRSERKAAERVRRLREEQQRERLRQVSRILRKAAAERS AEEGRLLAESEDLVTELQGRSRRREGLKRRQEEVCDDPEELRRKV RELAGAVRSARHLVVYTGAGISTAASIPDYRGPNGVWTLLQKGRP VSAADLSEAEPTLTHMSITRLHEQKLVQHVVSQNCDGLHLRSGLP RTAISELHGNMYIEVCTSCIPNREYVRVFDVTERTALHRHLTGRTC HKCGTQLRDTIVHFGERGTLGQPLNWEAATEAASKADTILCLGSS LKVLKKYPRLWCMTKPPSRRPKLYIVNLQWTPKDDWAALKLHGK CDDVMQLLMNELGLEIPVYNRWQDPIFSLATPLRAGEEGSHSRKS LCRSREEAPPGDQSDPLASAPPILGGWFGRGCAKRAKRKKVA

Bovine 613629	MADEAALALQPGGSPSVVAAEREAPSPPAGEPLRKRPRRDGPGVG RSSGEPGGTAPERELPAAAGSCPAAAAALWREAQAAAAAAAEEE DNGPGLQGLSREAPPADDFYDDDDEGEEEEEAAAAIGYRDNLLFG DEIITNGFHSCESDEDDRASHASSSDWTPRPRIGPYTFVQQHLMIG TDPRTILKDLLPETIPPPELDDMTLWQIVINILSEPPKRKKRKDINTIE DAVKLLQECKKIIVLTGAGVSVSCGIPDFRSRDGIYARLAIDFPDLP DPQAMFDIEYFRKDPRPFFKFAKEIYPGQFQPSLCHKFIALSDKEG KLLRNYTQNIDTLEQVAGIQKIIQCHGSFATASCLICKYKVDCEAVR GDIFNQVVPRCPRCPADEPLAIMKPEIVFFGENLPEQFHRAMKYDK DEVDLLIVIGSSLKVRPVALIPSSIPHEVPQILINREPLPHLHFDVELL GDCDVIINELCHRLGGEYAKLCCNPVKLSEITEKPPRIQKELAHLSE LPPTPLNISEGSSSPERTSPPDSSVIVTLLDQETKSNVDDPDVSESKD HVTEKSQEVQTSTRSIESVNEQLESPDLKNAVSNSGEKNERTSVAE TVRKCWPARLAKEQISKRLDDNQYLFLPPNRYIFHGAEVYSDSED DVLSSSSCGSNSDSGTCQSPSLEEPMEDESENEEFYNGLEDDADVN ERAGGTVFEADGGDQEAINEAISVKQEATCINYPSNKS
Bovine 504463	MAPGSCSSLTYLGPLLFPVPSRDRAVGDSPEGPRPAPMADPDPSDP EETQAGKVQEAQDSDSDTEAGATGGEAEMDFLRNFFSQTLGLGT QKERLLDELTLEGVSRYMQSERCRRVICLVGAGISTSAGIPDFRSPN TGLYANLEKYRLPYPEAIFEISYFKKHPEPFFALAKELYPGQFKPTIC HYFIRLLKEKGLLLRCYTQNIDTLERVAGLEPEDLVEAHGTFYTSH CISSGCRQEYSLSWMKEKIFSEVTPKCEKCQSVVKPDIVFFGENLP ARFFSCMQSDFLKVDLLIIMGTSLQVQPFASLIGKAPLSTPRLLINK EKTGQTDPFLGMMMALGGGMDFDSKKAYRDVAWLGDCDQGCL ALADLLGWKKELEDLVRKEHASIDAQSGSGASNPATSASPRNSPPP PTKEEPRTTEGEKPQ
Bovine 614027	MALFPRSAVPALRFWGLRGARAWTRWPRIPGGGWSISCFAGASSD TGGGDHSQKKFLLQDIAELIKTRACQKVVVMVGAGISTPSGIPDFR SPGVGYYSILQQYKLPYPEAIFELSFFFHDPKPFFTFAKKLYPGNYR PNATHYFLRLLHEKGLLLRLYTQNIDGLERASGIPDSKLVEAHGSL ASATCTVCRRPYPGEDFWADVMADRVPRCPVCSGVTKPDIVFFGE PLPARFLLHLADFPMADLLLILGTSLEVEPFASLSDAVRSSVPRLLIN RDLVGSLARNPRGRDVAQLGDVVHGVKRLVELLGWTDDIQDLIQ RETGKFDGWDRL
Bovine 519328	MRMSFGLTFKRTAKVHWRANFSQQCSLRSTGLFVPPSPPLDPEKV KELQRFITLSKRLLVMTGAGISTESGIPDYRSEKVGLYARTDRRPIQ HGDFVRSAPVRQRYWARNFVGWPQFSSRQPNPAHWALSNWERL GKLHWLVTQNVDALHTKAGSQRLTELHGCMHRVLCLDCGEQTPR GVLQERFQVLNPTWSAEAHGLAPDGDVFLTEEEVQSFQVPSCSRC GGPLKPDVVFFGDTVKPDKVDFVHKRVKEADSLLVVGSSLQVYS GYRFILTAREKKLPIVILNIGPTRSDDLASLKLDSRCGELLPLIDPR
Bovine 507347	MPPLWIIRNRLFSQLYCGLKSPVSTQTKICLTMARPSSNMADFRKC FAKAKHIVVISGAGISAESGVPTFRGAGGYWRKWKAQDLATPQAF ARNPSQVWEFYHYRREVVQSTEPNAGHLAIAECQARLHRQGRQV

	VVITQNIDELHRKAGTKNLLEIHGSLFKTRCTSCGVVAENYKSPICP<br>ALSGKGAPDPQTQDAGIPVEKLPRCEEAGCGGLLRPHVVWFGEN<br>LDPAILEEVDKELALCDLCLVVGTSSVVYPAAMFAPQVSARGVPV<br>AEFNMETTPATERFRFHFQGPCGTTLPEALAPHETETVS
Bovine 535416	MSVNYAAGLSPYADKGKCGLPEVFDPPEELEQKVWELAQLIWQS<br>SSVVFHTGAGISTASGIPDFRGPHGVWTMEERGLAPTFDTTFENAQ<br>PTKTHMALVQLERVGLLHFLVSQNVDGLHVRSGFPRDKLAELHG<br>NMFIEECVKCKMQYVRDTVVGSMGLKPTGRLCTVAKSRGLRACR<br>GELRDTILDWEDSLPDRDLTLADEASRNADLSITLGTSLQIRPSGNL<br>PLATKRRGGRLVIVNLQPTKHDRHADLRIHGYVDEVMTRLMKHL<br>GLEIPAWDGPHMVERALPPLPRPPAPKLEPKEEASPQLNSPVPANP<br>KQEPTAEPCTQHNGSGPTSPKRERPDSPSPHRPPKRVKTEVVPS
Bovine 505662	MAAGGLSRSERKAAERVRRLREEQQRERLRQVSRILRKAATERSA<br>EEGRLLAESEDLVTELQGRSRRREGLKRRQEEVCDDPEELQRKVR<br>ELASAVRNAKYLVVYTGAGISTAASIPDYRGPNGVWTLLQKGRSV<br>SAADLSEAEPTLTHMSITRLHEQKLVQHVVSQNCDGLHLRSGLPR<br>SAMSELHGNMYIEVCTACTPNREYVRVFDVTERTALHRHQTGRTC<br>HKCGGQLRDTIVHFGERGTLGQPLNWEAATEAASKADTILCLGSS<br>LKVLKKYPHLWCMTKPPSRRPKLYIVNLQWTPKDDWAALKLHGK<br>CDDVMQLLMDELGLEIPRYSRWQDPIFSLATPLRAGEEGSHSRKSL<br>CRSREEPGPGDRGAPLSSAPILGGWFGRGCTKRTKRKKVT

*, the 31 representative sirtuins that were heterologously expressed in *E. coli* Δ*cobB* cells. All the sequences were downloaded from KEGG.
